# Supplementary material for: BNIP3L/NIX-mediated mitophagy protects against glucocorticoid-induced synapse defects
Source: Nat Commun. 2021 Jan 20;12:487. doi: 10.1038/s41467-020-20679-y (PMC7817668; doi:10.1038/s41467-020-20679-y)
Supplement: Supplementary file 1 — Supplementary Information [file 41467_2020_20679_MOESM1_ESM.pdf]

Supplementary Fig 1

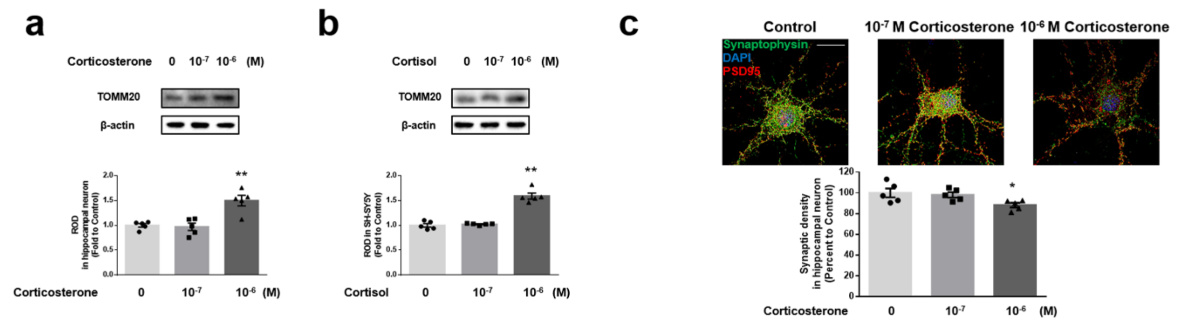

Fig 1. The dose-dependent effect of corticosterone and cortisol on mitophagy and synaptic density. (a – b) Hippocampal neurons and SH-SY5Y cells were treated with corticosterone and cortisol (0 – 1 μM) for 24 h, respectively. TOMM20 level was detected by western blot. Loading control is β-actin. (**c**) Hippocampal neurons were treated with corticosterone (0 – 1 μM) for 48 h. Hippocampal neurons were immunostained with synaptophysin (green), PSD95 (red), and DAPI (blue). Pearson's correlation coefficient was quantified for detecting synaptic density. Scale bars, 20 μm (magnification, × 1,000). *n*=5. All blots and immunofluorescence images are representative. *n*=5 from independent experiments with two technical replicates each. Quantitative data are presented as a mean ± S.E.M. The representative images were acquired by SRRF imaging system. Two-sided one-way ANOVA was conducted. *, ** indicates $p<0.05$, $p<0.01$ versus control, respectively. Data are provided as a Source data file.

Supplementary Fig 2

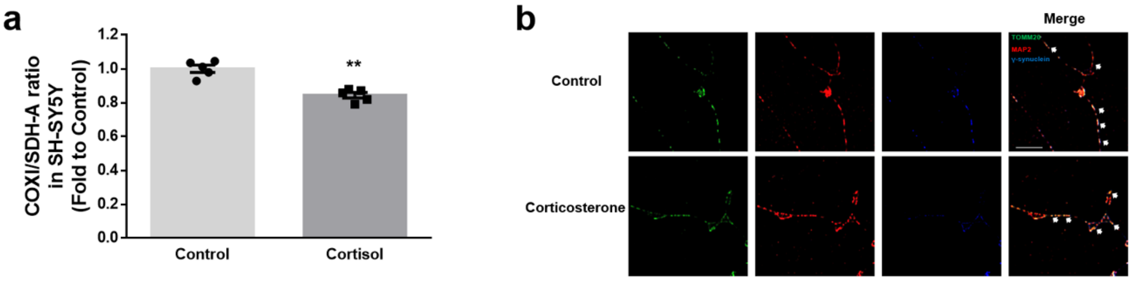

Fig 2. The effect of cortisol and corticosterone on mitochondrial biogenesis and transcellular mitophagy, respectively. (**a**) SH-SY5Y cells were treated with cortisol for 24 h. Subunit I of complex IV/70 kDa subunit of Complex II (COXI/SDH-A) ratio was measured to assess mitochondrial biogenesis. n=5 from independent experiments with two technical replicates each. Quantitative data are presented as a mean ± S.E.M. Two-sided unpaired student's t-test was conducted. ** indicates $p<0.01$ versus control. (**b**) Hippocampal neurons were treated with corticosterone for 24 h. Fixed coverslips were then immnunostained with TOMM20 (green), MAP2 (red), and γ-synuclein (blue). Scale bars, 200 μm (magnification, × 100). n=3 from independent experiments with two technical replicates each. The representative images were acquired by SRRF imaging system. Data are provided as a Source data file.

Supplementary Fig 3

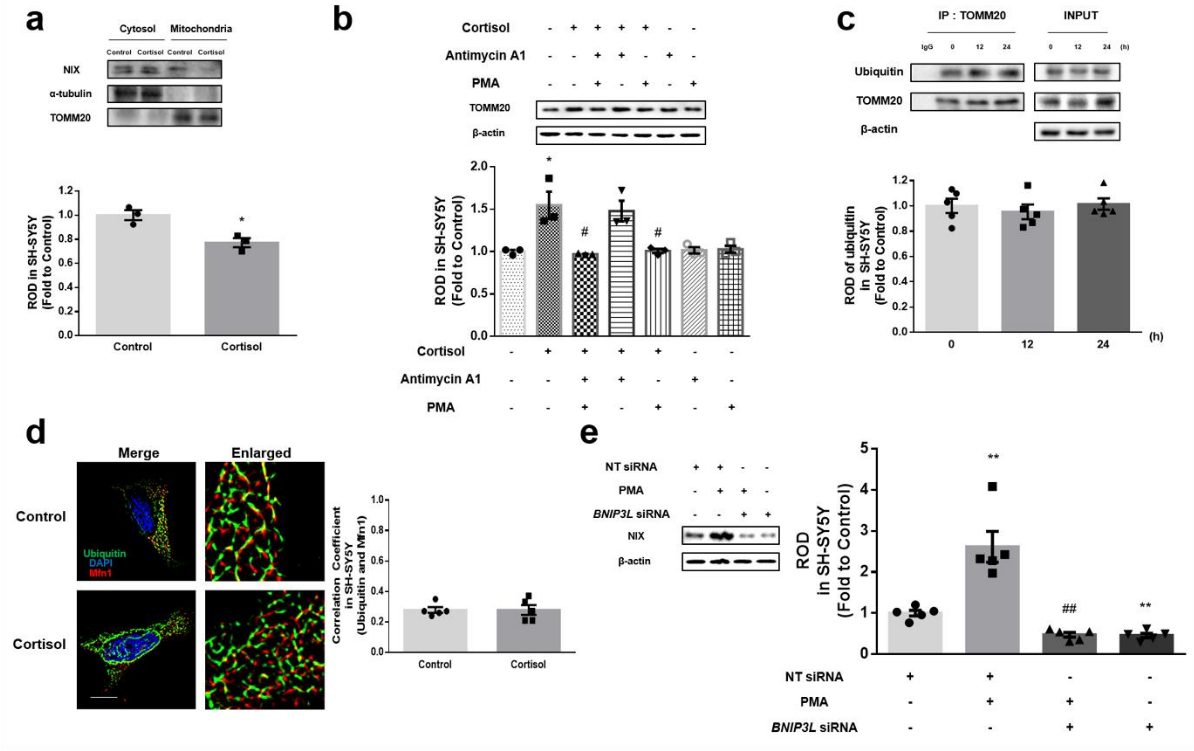

Fig 3. Decreases in mitochondrial NIX expression by cortisol inhibits mitophagy independently from the PINK1-parkin pathway. (**a**) SH-SY5Y cells were treated with cortisol for 24 h. NIX expressions in subcellular fraction samples were detected by western blotting. The α-tubulin and TOMM20 were used as cytosolic and mitochondrial loading control, respectively. The level of NIX in mitochondrial parts was quantified. n=3. Two-sided unpaired student's t-test was conducted. * indicates $p<0.05$ versus control. (**b**) SH-SY5Y cells were pre-treated with phorbol 12-myristate 13-acetate (PMA, 10 nM) or antimycin A (10 µM) for 30 min and incubated with cortisol for 24 h. TOMM20 levels were detected by western blot. Loading control is β-actin. n=3. * indicates $p<0.05$versus control and # indicates $p<0.05$ versus cortisol in SH-SY5Y, respectively. Two-sided two-way ANOVA was conducted. (**c**) SH-SY5Y cells were treated with cortisol for various time (0 – 24 h). TOMM20 was co-immunoprecipitated with ubiquitin. The level of ubiquitin in immunoprecipiated samples was quantified. n=5. Two-sided one-way ANOVA was conducted. The p value of 12 h and 24 h treatment of cortisol versus control is 0.5765 and 0.846, respectively. (**d**) SH-SY5Y cells were incubated with cortisol for 24 h. Co-localization of ubiquitin (green) and Mfn1 (red) was visualized with SRRF imaging system. DAPI was used for nuclear counterstaining (blue). Scale bars represent 20 µm (magnification, × 1,000). n=5. Correlation coefficient analysis using Pearson's coefficient value was done. Two-sided unpaired student's t-test was conducted. The p value versus

control is 0.99. (**e**) NT or *BNIP3L* siRNA was transfected to SH-SY5Y cells for 24 h prior to PMA (10 nM) for 24 h. The expressions of NIX were detected with western blot where β-actin was used as loading control. n=5. Two-sided two-way ANOVA was conducted. ** indicates $p<0.01$ versus control. $^{\#\#}$ indicates $p<0.01$ versus PMA. All blots and immunofluorescence images are representative. n=3 or 5 from independent experiments with two technical replicates each. Quantitative data are presented as a mean ± S.E.M. The representative images were acquired by SRRF imaging system. Data are provided as a Source data file.

Supplementary Fig 4

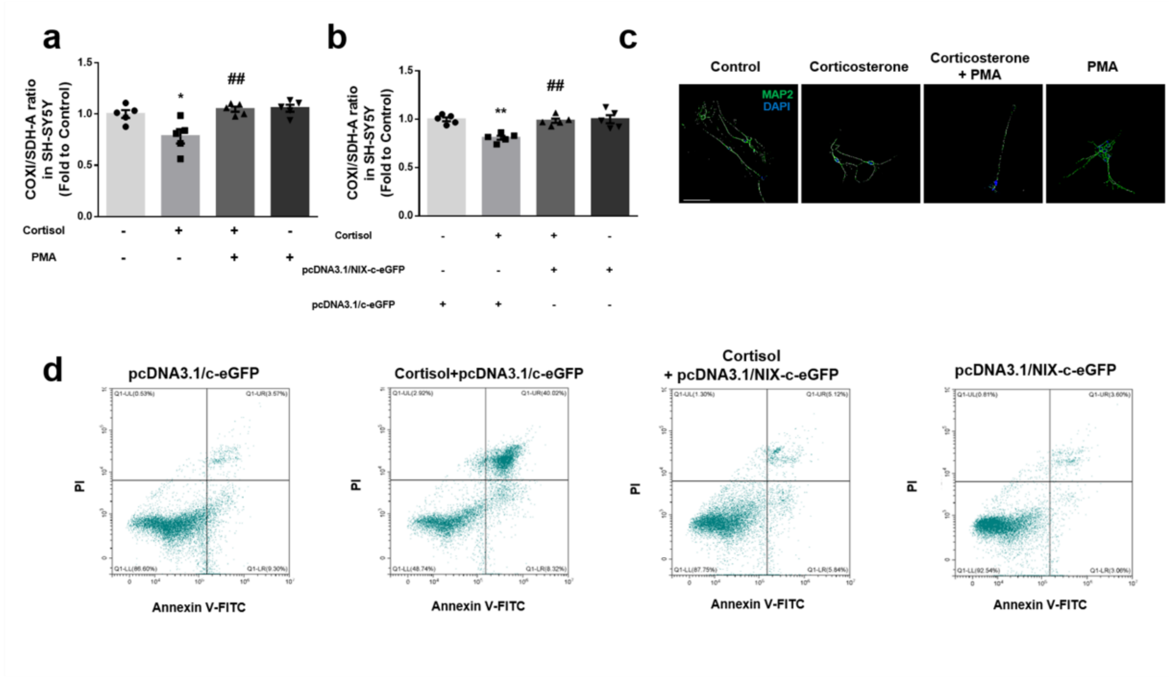

Fig 4. Glucocorticoid decreases mitochondrial biogenesis, synaptic function, and cell viability via inhibiting NIX expression. (**a**) SH-SY5Y cells were pretreated with phorbol 12-myristate 13-acetate (PMA, 10 nM) for 30 min prior to cortisol for 24 h. Subunit I of complex IV/70 kDa subunit of Complex II (COXI/SDH-A) ratio was measured to assess mitochondrial biogenesis. *n*=5. Two-sided two-way ANOVA was conducted. (**b**) SH-SY5Y cells were transfected with pcDNA3.1/c-eGFP or pcDNA3.1/NIX-c-eGFP vector for 24 h prior to cortisol treatment for 24 h. COXI/SDH-A ratio was measured to assess mitochondrial biogenesis. *n*=5. Two-sided two-way ANOVA was conducted. (**c**) Hippocampal neurons were treated with corticosterone for 48 h prior to PMA pretreatment (10 nM) for 30 min. Hippocampal neurons immnunostained with MAP2 (green) and DAPI (blue) for measuring lengths of dendrites are visualized using SRRF imaging system. Scale bars, 100 µm (magnification, × 200). *n*=5. (**d**) SH-SY5Y cells were transfected with pcDNA3.1/c-eGFP or pcDNA3.1/NIX-c-eGFP vector for 24 h prior to cortisol treatment for 72 h. The percentages of apoptotic cells were analyzed by Annexin V/PI analysis, measured by flowcytometer. Annexin V-FITC only positive cells undergo early apoptosis whereas PI positive only cells undergo necrosis. Both Annexin V-FITC and PI positive cells undergo late apoptosis Annexin V positive cells were considered as apoptotic cells. *n*=5. *n*=5 from independent experiments with two technical replicates each. Quantitative data are presented as a mean ± S.E.M. $^{*, **}$ indicates *p<0.05, p<0.01* versus control, respectively. $^{\#\#}$ indicates *p<0.01* versus cortisol, respectively. Data are provided as a Source data file.

Supplementary Fig 5

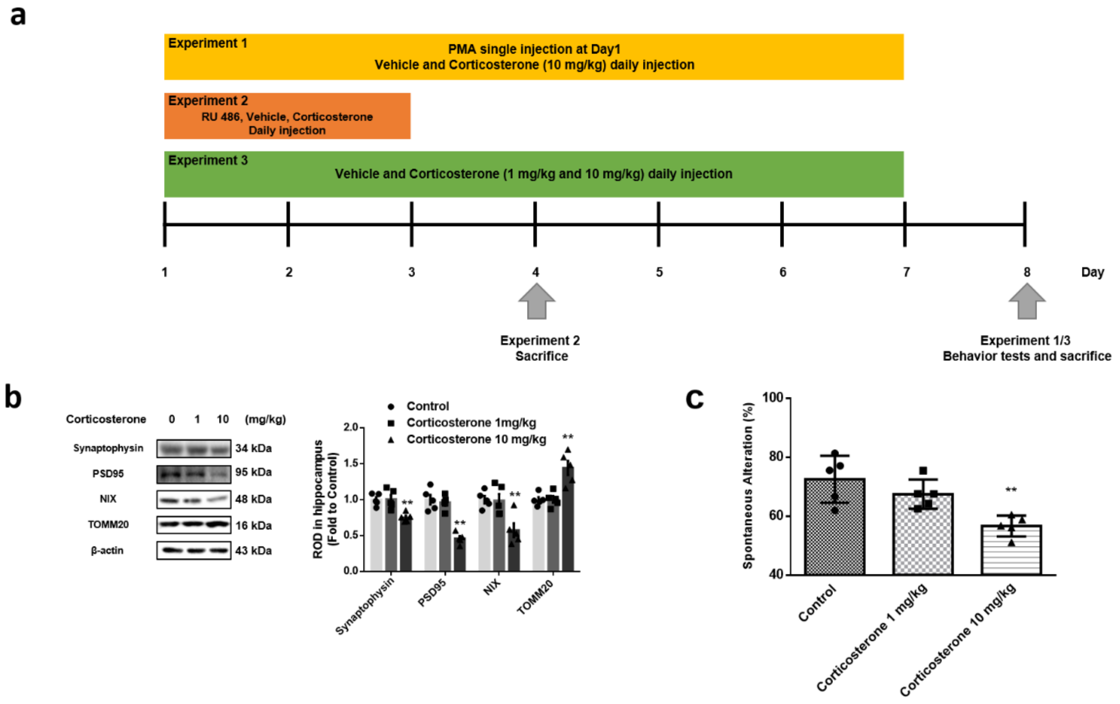

Fig 5. Stress-induced levels of corticosterone impairs mitophagy and spatial memory *in vivo*. (**a**) Brief illustration of drug injection schedule (**b-c**) Mice were exposed to vehicle and corticosterone (1 mg/kg and 10 mg/kg) for 7 days. (**b**) Expression of synaptophysin, PSD95, NIX, and TOMM20 were detected via western blot. Loading control is β-actin. *n*=5 from each animal with two technical replicates. (**c**) The mice were subjected to Y-maze test to evaluate spatial memory function. *n*=5. All blots and immunofluorescence images are representative. Quantitative data are presented as a mean ± S.E.M. Two-sided one-way ANOVA was conducted. ** indicates $p<0.01$ versus control and ## indicates $p<0.01$ versus corticosterone. Data are provided as a Source data file.

Supplementary Table

Figure	*p value*
**Fig 1a**	MAP2 and MTR: <0.0001<br>Tau and MTR: 0.0026
**Fig 1b**	Mitochondrial intensity: <0.0001 Pearson's correlation coefficient: <0.0001
**Fig 1c**	Perinuclear region: <0.0001<br>Distal region: 0.0125
**Fig 1d**	<0.0001
**Fig 1e**	<0.0001
**Fig 1f**	Synaptophysin: <0.0001<br>PSD95: 0.0004
**Fig 1g**	<0.0001
**Fig 1i**	<0.0001
**Fig 2a**	CCCP+Antimycin A1: <0.0001<br>Corticosterone: <0.0001
**Fig 2b**	CCCP+Antimycin A1: <0.0001<br>Cortisol: 0.0048
**Fig 2c**	<0.0001
**Fig 2d**	0.0026
**Fig 2e**	<0.0001
**Fig 2f**	<0.0001
**Fig 2g**	<0.0001
**Fig 2h**	<0.0001
**Fig 2i**	Cortisol: TOMM20 (0.0004), LC3II/I (0.0059)<br>Bafilomycin: TOMM20 (0.002), LC3II/I (0.0058)
**Fig 3a**	NIX: 0.0016
**Fig 3b**	NIX: <0.0001
**Fig 3c**	NIX 24 h: 0.001
**Fig 3d**	0.0072
**Fig 3e**	0.0003
**Fig 3f**	Versus control: 0.0002<br>Versus cortisol: <0.0001
**Fig 3g**	Versus control: <0.0001
**Fig 3h**	Versus control: 0.0002
**Fig 3i**	Versus control: <0.0001<br>Versus corticosterone: <0.0001
**Fig 3j**	Versus control: 0.0067<br>Versus cortisol: 0.0021
**Fig 3k**	Versus control: 0.0002<br>Versus corticosterone: 0.0009
**Fig 3l**	Versus control: <0.0001<br>Versus cortisol: <0.0001
**Fig 3m**	Versus control: 0.0001<br>Versus cortisol: 0.0017

Figure	*p value*
**Fig 4a**	Versus control: 0.0176<br>Versus corticosterone: 0.0134
**Fig 4b**	Versus control: 0.0038<br>Versus cortisol: 0.003
**Fig 4c**	Versus control: 0.0153<br>Versus corticosterone: 0.0003
**Fig 4d**	Versus control: <0.0001<br>Versus cortisol: 0.0003
**Fig 4f**	Basal respiration:<br>versus control (0.0005)<br>versus corticosterone (<0.0001)<br>Maximal respiration:<br>versus control (0.0003)<br>versus corticosterone (<0.0001)<br>ATP production:<br>versus control (0.0007)<br>versus corticosterone (<0.0001)<br>Proton leak:<br>versus control (0.0003)<br>versus corticosterone (<0.0001)
**Fig 4h**	Basal respiration:<br>versus control (0.0018)<br>versus cortisol (0.0098<br>Maximal respiration:<br>versus control (0.0004)<br>versus cortisol (<0.0001)<br>ATP production:<br>versus control (0.0004)<br>versus cortisol (0.0005)<br>Proton leak:<br>versus control (<0.0001)<br>versus cortisol (0.0003)
**Fig 4i**	Synaptophysin:<br>versus control (<0.0001)<br>versus corticosterone (0.0008)<br>PSD95:<br>versus control (0.0003)<br>versus corticosterone (<0.0001)
**Fig 4j**	Versus control: <0.0001<br>Versus corticosterone: 0.0001
**Fig 5a**	Versus control: <0.0001<br>Versus corticosterone: <0.0001
**Fig 5b**	Versus control: 0.0011<br>Versus cortisol: 0.004
**Fig 5c**	Versus control: 0.0128<br>Versus corticosterone: 0.0048
**Fig 5d**	Versus control: 0.0035<br>Versus cortisol: 0.0103

Figure	*p value*
**Fig 6a**	Versus control: 0.0001<br>Versus corticosterone: 0.002
**Fig 6b**	Versus control: <0.0001<br>Versus cortisol: 0.0002
**Fig 6e**	0.0045
**Fig 7a**	Versus control: 0.0008<br>Versus corticosterone: 0.0011
**Fig 7b**	Versus control: 0.001<br>Versus cortisol: 0.0134
**Fig 7c**	Versus control: 0.001<br>Versus corticosterone: 0.0054
**Fig 7d**	Versus control: <0.0001<br>Versus cortisol: <0.0001
**Fig 7e**	Versus control: 0.0001<br>Versus cortisol: 0.0004
**Fig 7f**	Versus control: 0.0005<br>Versus cortisol: 0.0454
**Fig 7g**	Versus control: 0.0002<br>Versus cortisol: 0.0004
**Fig 8a**	Versus control: 0.0008<br>Versus corticosterone: 0.0037
**Fig 8b**	NIX: 0.0014
**Fig 8c**	Versus control: 0.001<br>Versus corticosterone: 0.0054
**Fig 8d**	Versus control: 0.001<br>Versus corticosterone: 0.0054
**Fig 8e**	Versus control: 0.001<br>Versus corticosterone: 0.0054
**Fig 8g**	Versus control: 0.001<br>Versus corticosterone: 0.0054
**Supplementary figure**	***p* value**
**Fig 1a**	1 μM corticosterone (0.002)
**Fig 1b**	1 μM cortisol (<0.0001)
**Fig 1c**	1 μM corticosterone (0.0391)
**Fig 2a**	0.0004
**Fig 3a**	0.0154
**Fig 3b**	Versus control: 0.0277<br>Versus cortisol:<br>Cortisol+Antimycin A1+PMA (0.023)<br>Cortisol+PMA (0.0295)
**Fig 3e**	Versus control: PMA (0.0028)<br>BNI3L siRNA (0.0003)<br>Versus PMA: 0.0005
**Fig 4a**	Versus control: 0.0259<br>Versus cortisol: 0.0072
**Fig 4b**	Versus control: 0.0002<br>Versus cortisol: 0.0004
**Fig 5b**	10 mg corticosterone:<br>Synaptophysin (0.0007)<br>PSD95 (<0.0001)<br>NIX (0.0043)<br>TOMM20 (0.0031)
**Fig 5c**	10 mg corticosterone (0.0036)

Table. The exact *p* value for each figure.
