# Supplementary material for: BNIP3L/NIX-mediated mitophagy protects against glucocorticoid-induced synapse defects
Source: Nat Commun. 2021 Jan 20;12:487. doi: 10.1038/s41467-020-20679-y (PMC7817668; doi:10.1038/s41467-020-20679-y)
Supplement: Supplementary file 3 — Reporting Summary [file 41467_2020_20679_MOESM3_ESM.pdf]

nature research

Corresponding author(s): Ho Jae Han
Last updated by author(s): Dec 11, 2020

Reporting Summary

Nature Research wishes to improve the reproducibility of the work that we publish. This form provides structure for consistency and transparency in reporting. For further information on Nature Research policies, see our Editorial Policies and the Editorial Policy Checklist.

Statistics

For all statistical analyses, confirm that the following items are present in the figure legend, table legend, main text, or Methods section.

n/a	Confirmed	
☐	☒	The exact sample size (n) for each experimental group/condition, given as a discrete number and unit of measurement
☐	☒	A statement on whether measurements were taken from distinct samples or whether the same sample was measured repeatedly
☐	☒	The statistical test(s) used AND whether they are one- or two-sided<br>*Only common tests should be described solely by name; describe more complex techniques in the Methods section.*
☒	☐	A description of all covariates tested
☒	☐	A description of any assumptions or corrections, such as tests of normality and adjustment for multiple comparisons
☐	☒	A full description of the statistical parameters including central tendency (e.g. means) or other basic estimates (e.g. regression coefficient) AND variation (e.g. standard deviation) or associated estimates of uncertainty (e.g. confidence intervals)
☐	☒	For null hypothesis testing, the test statistic (e.g. F, t, r) with confidence intervals, effect sizes, degrees of freedom and P value noted<br>*Give P values as exact values whenever suitable.*
☒	☐	For Bayesian analysis, information on the choice of priors and Markov chain Monte Carlo settings
☒	☐	For hierarchical and complex designs, identification of the appropriate level for tests and full reporting of outcomes
☐	☒	Estimates of effect sizes (e.g. Cohen's d, Pearson's r), indicating how they were calculated

Our web collection on statistics for biologists contains articles on many of the points above.

Software and code

Policy information about availability of computer code

Data collection	CytExpert software version 2.3 provided from BECKMAN COULTER (Fullerton, CA, USA) was used for data collection during flow cytometry analysis.<br>SMART 3.0 video tracking system provided from Panlab/Harvard apparatus (Holliston, MA, USA) was used for data collection during forced swim test. OCR data were collected by XF24 Extracellular Flux Analyzer (Agilent Technologies, Santa Clara, CA, USA).
Data analysis	Graph design and statistical analysis were performed with Graph pad prism Version 6.0<br>Immunocytochemistry and immunohistochemistry images were analyzed using Fiji version 1.53.<br>FM4-64 kinetics were analyzed using Image J version 1.53.<br>PCR array data were analyzed by GeneGlobe Data analysis center on Qiagen's website.<br>Results from forced swim test were analyzed by SMART 3.0 video tracking software.

For manuscripts utilizing custom algorithms or software that are central to the research but not yet described in published literature, software must be made available to editors and reviewers. We strongly encourage code deposition in a community repository (e.g. GitHub). See the Nature Research guidelines for submitting code & software for further information.

Data

Policy information about availability of data

All manuscripts must include a data availability statement. This statement should provide the following information, where applicable:

- Accession codes, unique identifiers, or web links for publicly available datasets
- A list of figures that have associated raw data
- A description of any restrictions on data availability

The authors declare that all the data supporting the findings of this study are available within this article, its supplementary information files, or are available from the corresponding authors upon reasonable request. Source data are provided with this paper.

Field-specific reporting

Please select the one below that is the best fit for your research. If you are not sure, read the appropriate sections before making your selection.

☒ Life sciences ☐ Behavioural & social sciences ☐ Ecological, evolutionary & environmental sciences

For a reference copy of the document with all sections, see nature.com/documents/nr-reporting-summary-flat.pdf

Life sciences study design

All studies must disclose on these points even when the disclosure is negative.

Sample size	Applying size of samples (minimum of n = 3) can be acceptable if very low p values are observed rather than the large size of N including interfering results. Therefore, we set the minimum of n = 3 (western blotting, immunocytochemistry, PCR array) and n = 5 (behavior test) independent experiments to gain statistical powers according to the previous published article of Brain. We performed two technical replicates each.
Data exclusions	No data were excluded.
Replication	The sample size 'n' shown in the figure legends represents the number of biological independent replicates and statistical analyses were performed using these independent values. Two technical replications were performed throughout the study.
Randomization	Male ICR mice aged 7 weeks were used and randomly housed 6 per cage under standard environmental conditions. Allocation of samples throughout in vitro experiments were randomly done to minimize the effects of subjective bias.
Blinding	Investigators were blinded to group allocation during data collection and analysis of in vivo and in vitro experiments.

Reporting for specific materials, systems and methods

We require information from authors about some types of materials, experimental systems and methods used in many studies. Here, indicate whether each material, system or method listed is relevant to your study. If you are not sure if a list item applies to your research, read the appropriate section before selecting a response.

Materials & experimental systems

n/a	Involved in the study	
☐	☒	Antibodies
☐	☒	Eukaryotic cell lines
☒	☐	Palaeontology and archaeology
☐	☒	Animals and other organisms
☒	☐	Human research participants
☒	☐	Clinical data
☒	☐	Dual use research of concern

Methods

n/a	Involved in the study	
☒	☐	ChIP-seq
☐	☒	Flow cytometry
☒	☐	MRI-based neuroimaging

Antibodies

Antibodies used	The antibodies of BNIP3 (#sc-56167), Lamin A/C (#sc-20681), and β-actin (#sc-47778) were obtained from Santa Cruz Biotechnology (Paso Robles, CA, USA). The antibodies of TOMM20 (#ab56783), synaptophysin (#ab32127), γ-synuclein (#ab55424), MAP2 (#ab11267), LAMP1 (#ab24170), and parkin (#ab77924) were purchased from Abcam (Cambridge, MA, USA). The GR antibody (#12041S) was obtained from Cell Signaling Technology, Inc. (Danvers, MA, USA). The antibodies of LC3 (#NB100-2220), PGC1α (#NBP1-04676), NIX (#NBP1-88558), and PINK1 (#BC100-494) were purchased from Novus Biologicals (Littleton, CO, USA). The antibodies of synaptotagmin-1 (#PA5-27935), Tau5 (#AHB0042), ubiquitin (#701339), Alexa Fluor secondary antibodies (#A48255, #A32723, #A32731, #A32727, #A32732) , and HRP-conjugated secondary antibodies (#G-21040, #G-21234) were obtained from

Thermo Fisher (Rockford, IL, USA). Mfn1 antibody (#66776-1-lg) was acquired from Proteintech (Rosemont, IL, USA).), The antibodies of PSD95 (#MAB1596) and α-tubulin (#T6074) were purchased from Sigma Chemical Company (St. Louis, MO, USA).

Validation

All antibodies used in this study are commercially available and antibody validation was performed by the individual manufacturer; validation information can be found on the manufacturers' web pages via the links provided below.
Individual statements:
Abcam: https://www.abcam.com/primary-antibodies/improving-reproducibility-with-better-antibodies
Cell signaling: https://www.cellsignal.com/contents/our-approach/cst-antibody-validation-principles/ourapproach-validation-principless
Novus: https://www.novusbio.com/5-pillars-vadliation
Thermo Fisher: https://www.thermofisher.com/us/en/home/life-science/antibodies/invitrogen-antibody-validation.html

List of primary antibodies:

1. Primary antibodies from Santa Cruz Biotechnology

BNIP3 (#sc-56167)
Application: Western blot; Species: Mouse; Monoclonal ANa40; Manufacturer's validation information: https://datasheets.scbt.com/sc-56167.pdf
Lamin A/C (#sc-20681)
Application: Western blot; Species: Rabbit; Polyclonal H-100; Manufacturer's validation information: https://www.scbt.com/ko/p/lamin-a-c-antibody-h-110
β-actin (#sc-47778)
Application: Western blot; Species: Mouse; Monoclonal C4; Manufacturer's validation information: https://datasheets.scbt.com/sc-47778.pdf

2. Primary antibodies from Abcam
TOMM20 (#ab56783)
Application: Western blot, Immunostaining, Immunoprecipitation; Species: Mouse; Monoclonal; Manufacturer's validation information: https://www.abcam.com/tomm20-antibody-mitochondrial-marker-ab56783.html
Synaptophysin (#ab32127)
Application: Western blot, Immunostaining; Species:; Rabbit; Monoclonal YE269; Manufacturer's validation information: https://www.abcam.com/synaptophysin-antibody-ye269-ab32127.html
γ-synuclein (#ab55424)
Application: Immunostaining; Species:Rabbit; Polyclonal; Manufacturer's validation information: https://www.abcam.com/gamma-synucleinsncg-antibody-ab55424.html
MAP2 (#ab11267)
Application: Immunostaining; Species: Mouse; Monoclonal HM-2; Manufacturer's validation information: https://www.abcam.com/map2-antibody-hm-2-ab11267.html
LAMP1 (#ab24170)
Application: Immunostaining; Species: Rabbit; Polyclonal; Manufacturer's validation information: https://www.abcam.com/lamp1-antibody-lysosome-marker-ab24170.html
Parkin (#ab77924)
Application: Western blot;Species: Mouse; Monoclonal PRK8; Manufacturer's validation information: https://www.abcam.com/parkin-antibody-prk8-ab77924.html

3. Primary antibody from Cell signaling

GR antibody (#12041S)
Application: Immunostaining, ChIP assay; Species: Rabbit; Monoclonal D6H2L; Manufacturer's validation information: https://www.cellsignal.com/products/primary-antibodies/glucocorticoid-receptor-d6h2l-xp-rabbit-mab/12041

4. Primary antibodies from Novus

LC3 (#NB100-2220),
Application: Western blot; Species: Rabbit; Polyclonal; Manufacturer's validation information: https://www.novusbio.com/products/lc3b-antibody_nb100-2220
PGC1α (#NBP1-04676)
Application: Western blot, Immunostaining; Species: Rabbit; Polyclonal ; Manufacturer's validation information: https://www.novusbio.com/products/pgc1-alpha-antibody_nbp1-04676
NIX (#NBP1-88558)
Application: Western blot, Immunostaining, Immunoprecipitation; Species: Rabbit; Polyclonal; Manufacturer's validation information: https://www.novusbio.com/products/bnip3l-antibody_nbp1-88558
PINK1 (#BC100-494)
Application: Western blot; Species: Rabbit; Polyclonal; Manufacturer's validation information: https://www.novusbio.com/products/pink1-antibody_bc100-494

5. Primary antibodies from Thermo Fisher

Synaptotagmin-1 (#PA5-27935)
Application: Immunostaining; Species: Rabbit; Polyclonal; Manufacturer's validation information: https://www.thermofisher.com/order/genome-database/DataSheetPdfproducttype=antibody&productsubtype=antibody_primary&productId=PA5-27935&version=125
Tau5 (#AHB0042)
Application: Immunostaining; Species: Mouse; Monoclonal TAU-5; Manufacturer's validation information: https://www.thermofisher.com/order/genome-database/dataSheetPdf?

producttype=antibody&productsubtype=antibody_primary&productId=AHB0042&version=125
Ubiquitin (#701339)
Application: Western blot, Immunostaining; Species: Rabbit; Monoclonal 10H4L21; Manufacturer's validation information: https://www.thermofisher.com/order/genome-database/dataSheetPdf?producttype=antibody&productsubtype=antibody_primary&productId=701339&version=125

6. Primary antibody from Proteintech

Mfn1 antibody (#66776-1-lg)
Application: Immunostaining; Species:; Mouse; Monoclonal 3F11C11; Manufacturer's validation information: https://www.ptglab.com/products/MFN1-Antibody-66776-1-lg.htm#protocols

7. Primary antibodies from Sigma Chemical Company

PSD95 (#MAB1596):
Application: Western blot, Immunostaining; Species:; Mouse; Monoclonal 6G6-1C9; Manufacturer's validation information: https://www.sigmaaldrich.com/catalog/product/mm/mab1596?lang=ko&region=KR&gclid=Cj0KCQiA5bz-BRD-ARIsABjT4nhPFD3FHDBzV-qlhxwCPHv1zqzkTpLSUZFbqnFx0FqAlDy1medOPacaAuOoEALw_wcB
α-tubulin (#T6074)
Application: Western blot; Species: Mouse; Monoclonal B-5-1-2; Manufacturer's validation information: https://www.sigmaaldrich.com/catalog/product/sigma/t6074?lang=ko&region=KR

Eukaryotic cell lines

Policy information about cell lines

Cell line source(s)	The human neuroblastoma cell line SH-SY5Y were acquired by Korean Cell Line Bank (Seoul, Korea).
Authentication	We did not independently authenticate the SH-SY5Y cell line.
Mycoplasma contamination	The cells were not tested for Mycoplasma contamination.
Commonly misidentified lines (See ICLAC register)	No commonly misidentified cell lines were used in the study

Animals and other organisms

Policy information about studies involving animals; ARRIVE guidelines recommended for reporting animal research

Laboratory animals	Male ICR mice aged 7 weeks were used for in vivo experiments. Pregnant female ICR mice aged 8-9 weeks were used for in vitro experiments (primary hippocampal neuron culture). Hippocampal neurons from embryonic day 18 of mouse embryos were used.
Wild animals	This study does not contain wild animals.
Field-collected samples	This study does not contain field collected samples.
Ethics oversight	The experiments were designed in compliance with the ARRIVE guidelines. Male ICR mice aged 7 weeks were used, in compliance and approval with the Institutional Animal Care and Use Committee of Seoul National University (SNU-190917-6). Hippocampal neurons from E18 mouse embryos were used in compliance and approval with the Institutional Animal Care and Use Committee of Seoul National University (SNU-190523-1-1).

Note that full information on the approval of the study protocol must also be provided in the manuscript.

Flow Cytometry

Plots

Confirm that:

☒ The axis labels state the marker and fluorochrome used (e.g. CD4-FITC).

☒ The axis scales are clearly visible. Include numbers along axes only for bottom left plot of group (a 'group' is an analysis of identical markers).

☒ All plots are contour plots with outliers or pseudocolor plots.

☒ A numerical value for number of cells or percentage (with statistics) is provided.

Methodology

Sample preparation	SH-SY5Y cell lines for detecting apoptosis were used. We used Annexin V-FITC/PI staining using Annexin V-FITC apoptosis detectin kit (#BD 556547, BD Bioscience, Franklin Lakes, NJ, USA). The sample preparation were performed following the manufacturer's instructions. After treatment, SH-SY5Y cells were suspended in binding buffer. Then Annexin V-FITC and PI were added to the samples and incubated for 15 min at room temperature. Apoptosis of the samples was detected with CytoFlex flow cytometry (Quanta SC; Beckman Coulter).

Instrument	Flow cytometry data were collected using CytoFlex provided from Quanta SC; Beckman Coulter.
Software	CytExpert software 2.3 provided from BECKMAN COULTER (Fullerton, CA, USA) was used for data collection during flow cytometry analysis
Cell population abundance	At least 10,000 events were modeled for each condition.
Gating strategy	SH-SY5Y cells were used. Events were plotted as Annexin V/PI. Annexin V-FITC positive cells undergo apoptosis. PI positive cells/Annexin V negative cells undergo necrosis. Both Annexin V and PI positive cells undergo late apoptosis.

☒ Tick this box to confirm that a figure exemplifying the gating strategy is provided in the Supplementary Information.
