# Supplementary figures and images for: BNIP3L/NIX-mediated mitophagy protects against glucocorticoid-induced synapse defects
Source: Nat Commun. 2021 Jan 20;12:487. doi: 10.1038/s41467-020-20679-y (PMC7817668; doi:10.1038/s41467-020-20679-y)

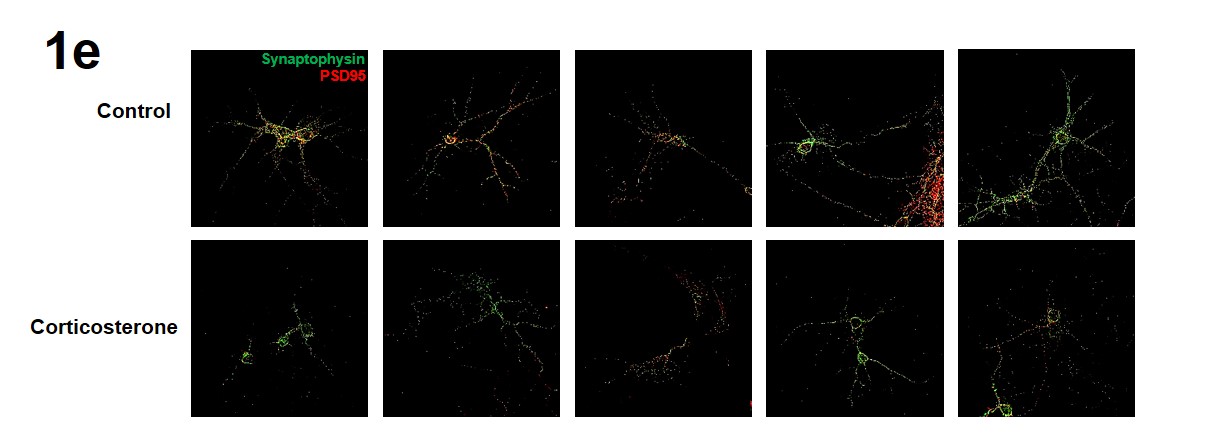

Supplement: Supplementary file 4 — Source Data [file 41467_2020_20679_MOESM4_ESM.zip › Fig 1/Fig 1e.jpg]

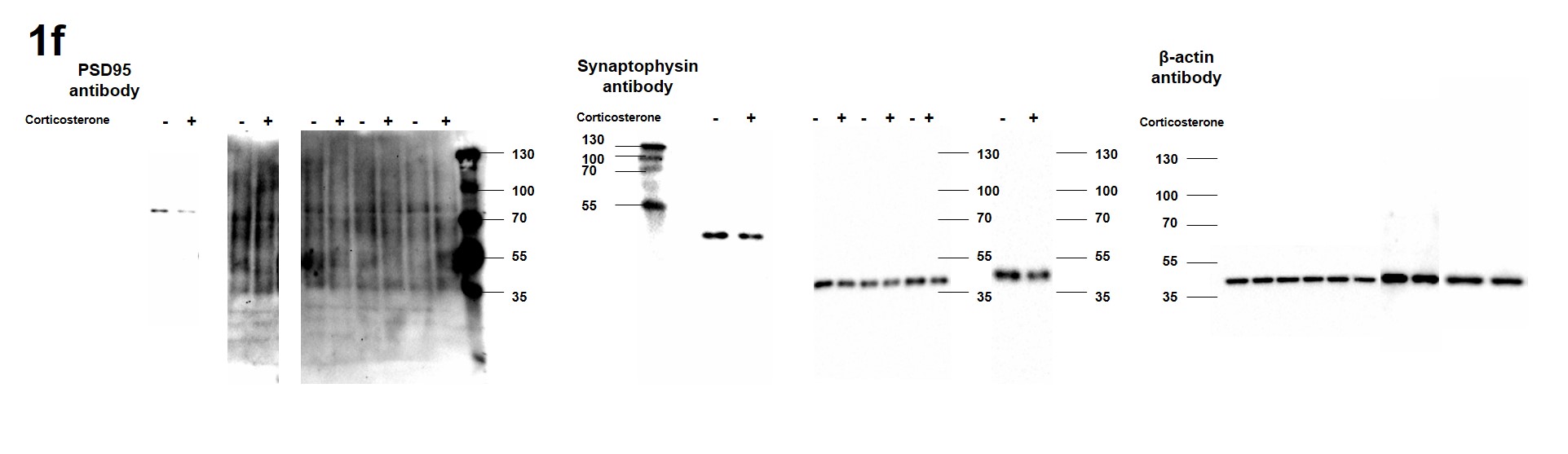

Supplement: Supplementary file 4 — Source Data [file 41467_2020_20679_MOESM4_ESM.zip › Fig 1/Fig 1f.jpg]

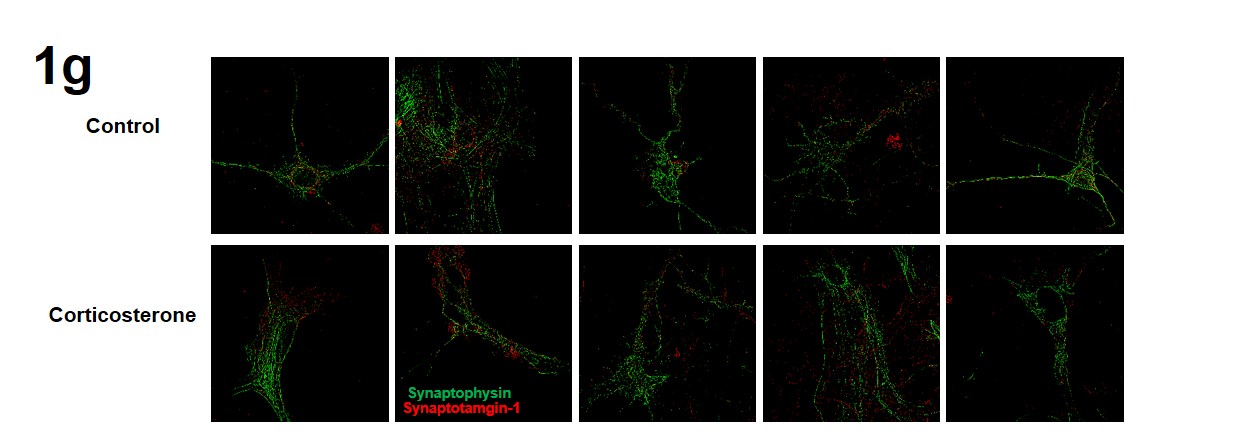

Supplement: Supplementary file 4 — Source Data [file 41467_2020_20679_MOESM4_ESM.zip › Fig 1/Fig 1g.jpg]

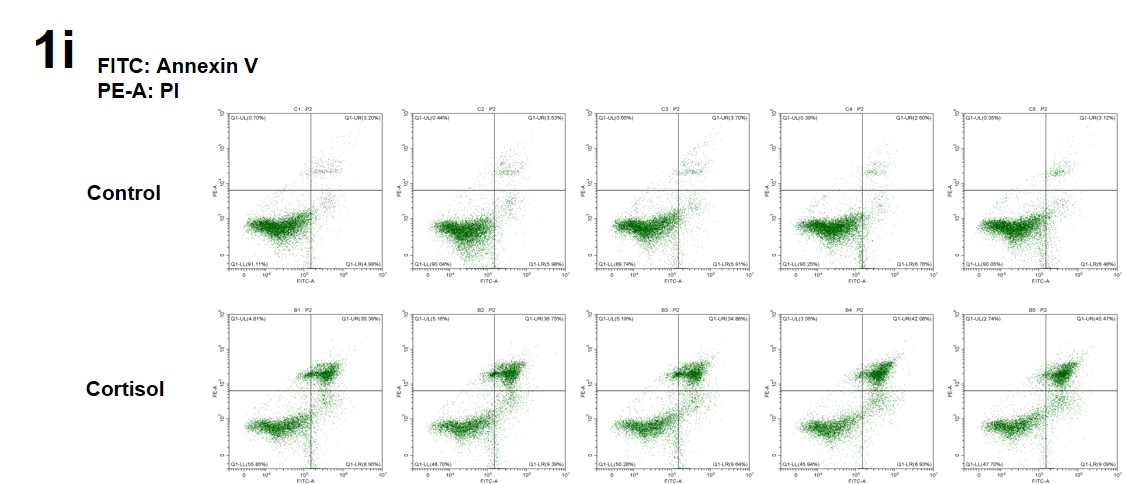

Supplement: Supplementary file 4 — Source Data [file 41467_2020_20679_MOESM4_ESM.zip › Fig 1/Fig 1i.jpg]

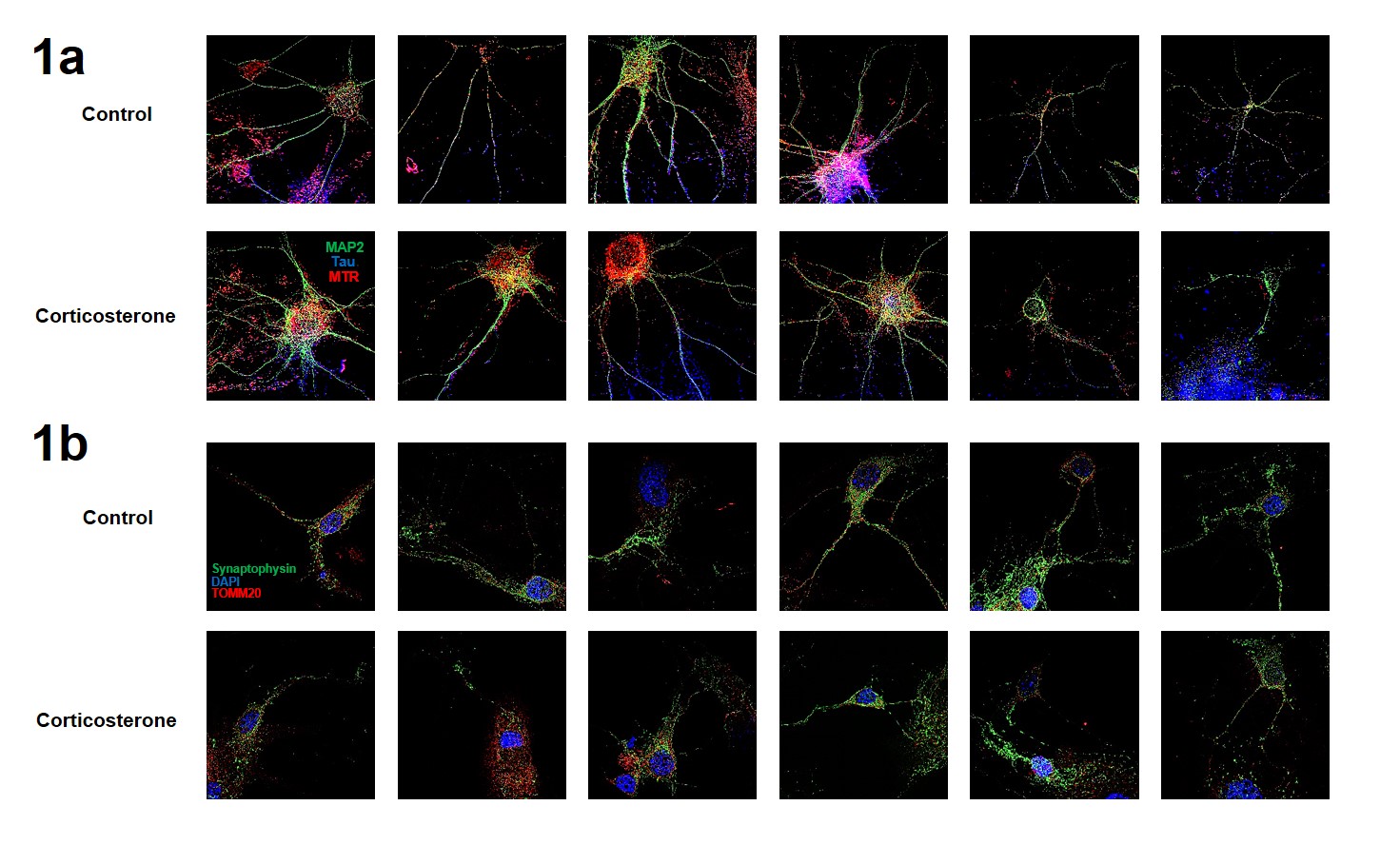

Supplement: Supplementary file 4 — Source Data [file 41467_2020_20679_MOESM4_ESM.zip › Fig 1/Figs 1a-1b.jpg]

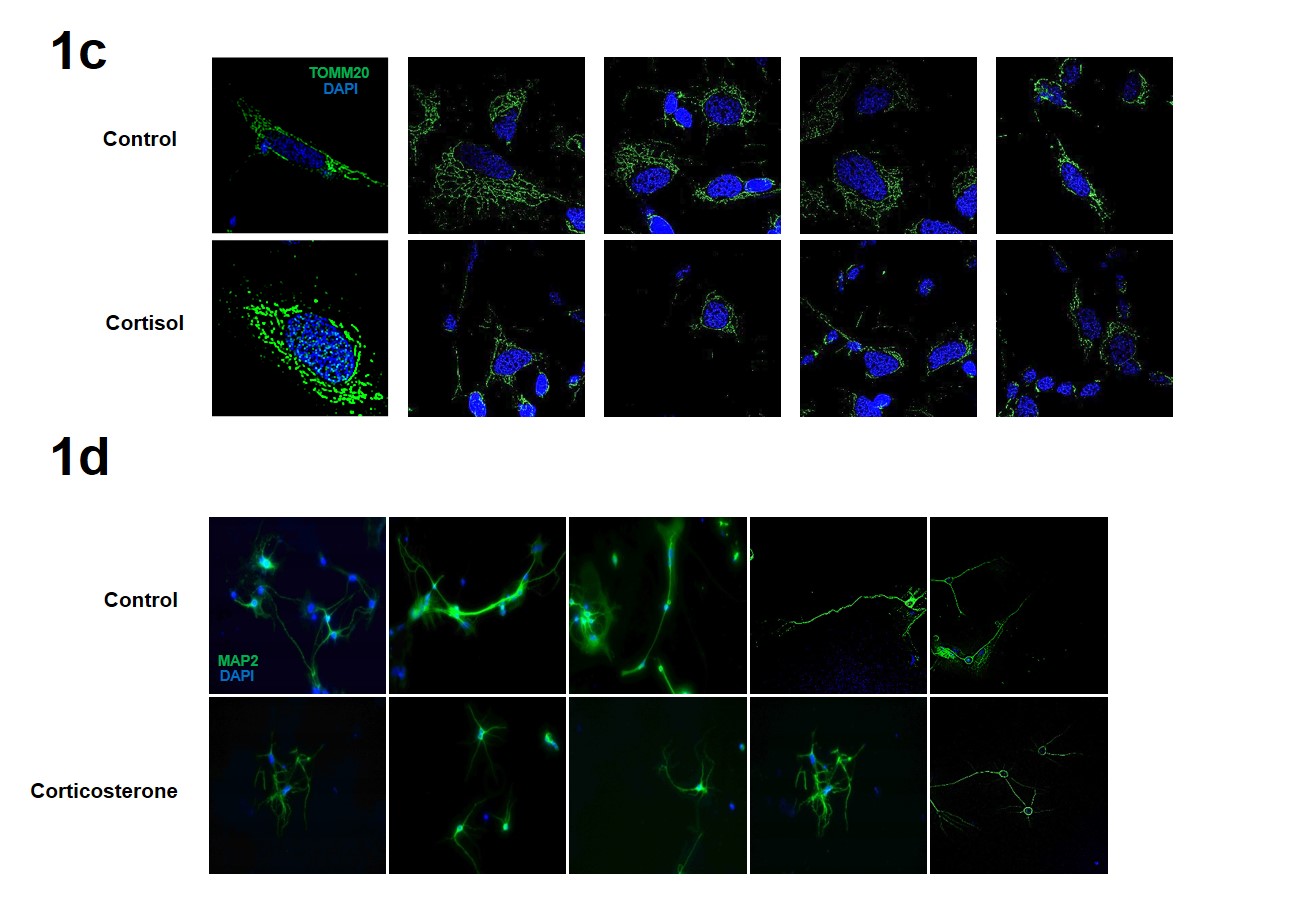

Supplement: Supplementary file 4 — Source Data [file 41467_2020_20679_MOESM4_ESM.zip › Fig 1/Figs 1c-1d.jpg]

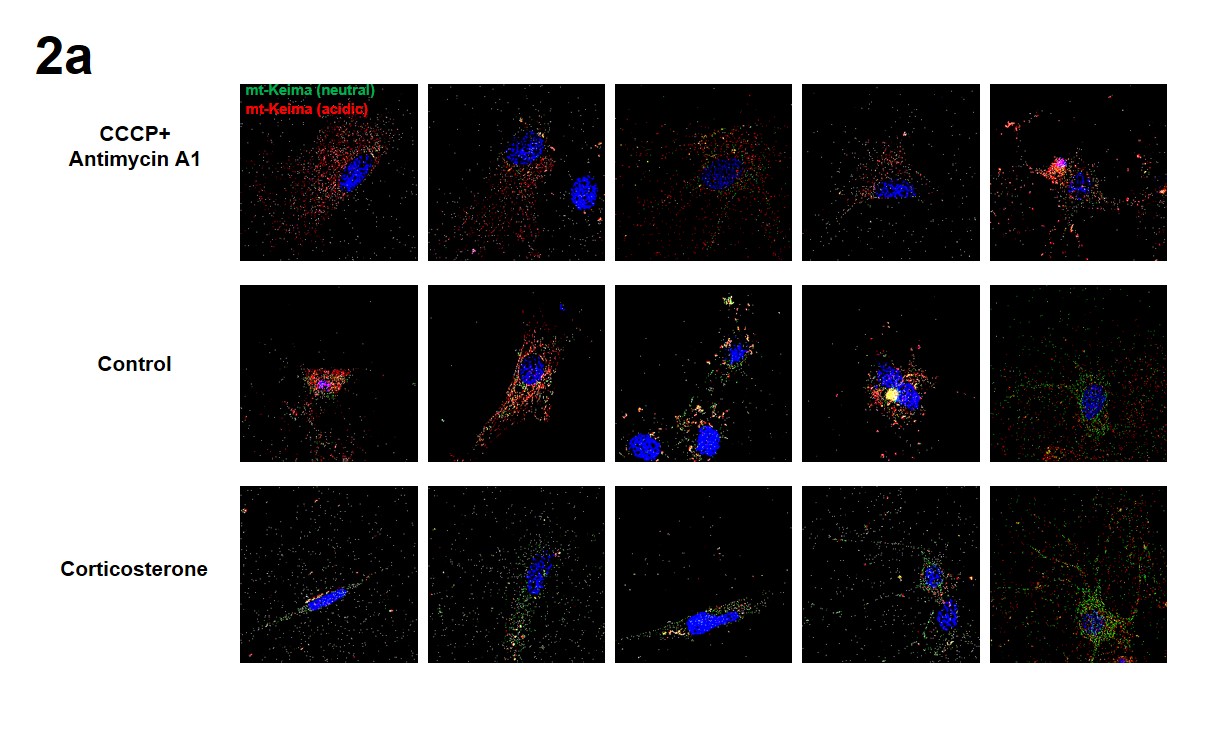

Supplement: Supplementary file 4 — Source Data [file 41467_2020_20679_MOESM4_ESM.zip › Fig 2/Fig 2a.jpg]

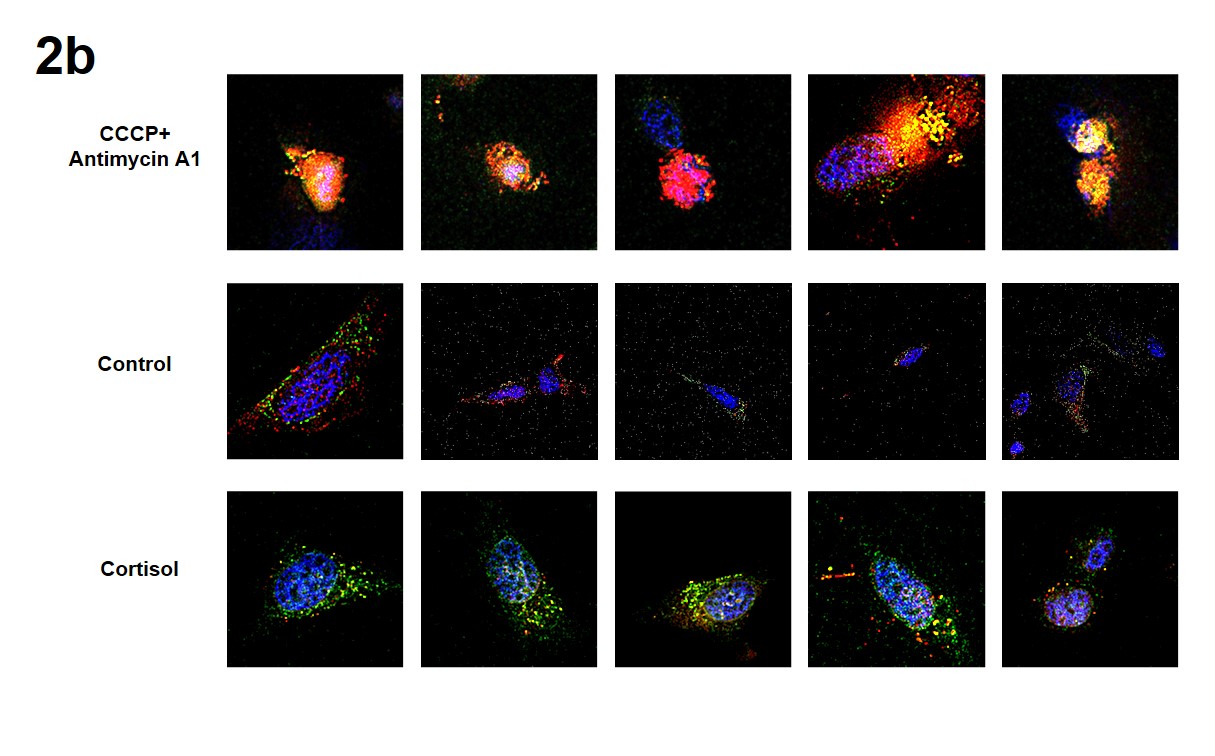

Supplement: Supplementary file 4 — Source Data [file 41467_2020_20679_MOESM4_ESM.zip › Fig 2/Fig 2b.jpg]

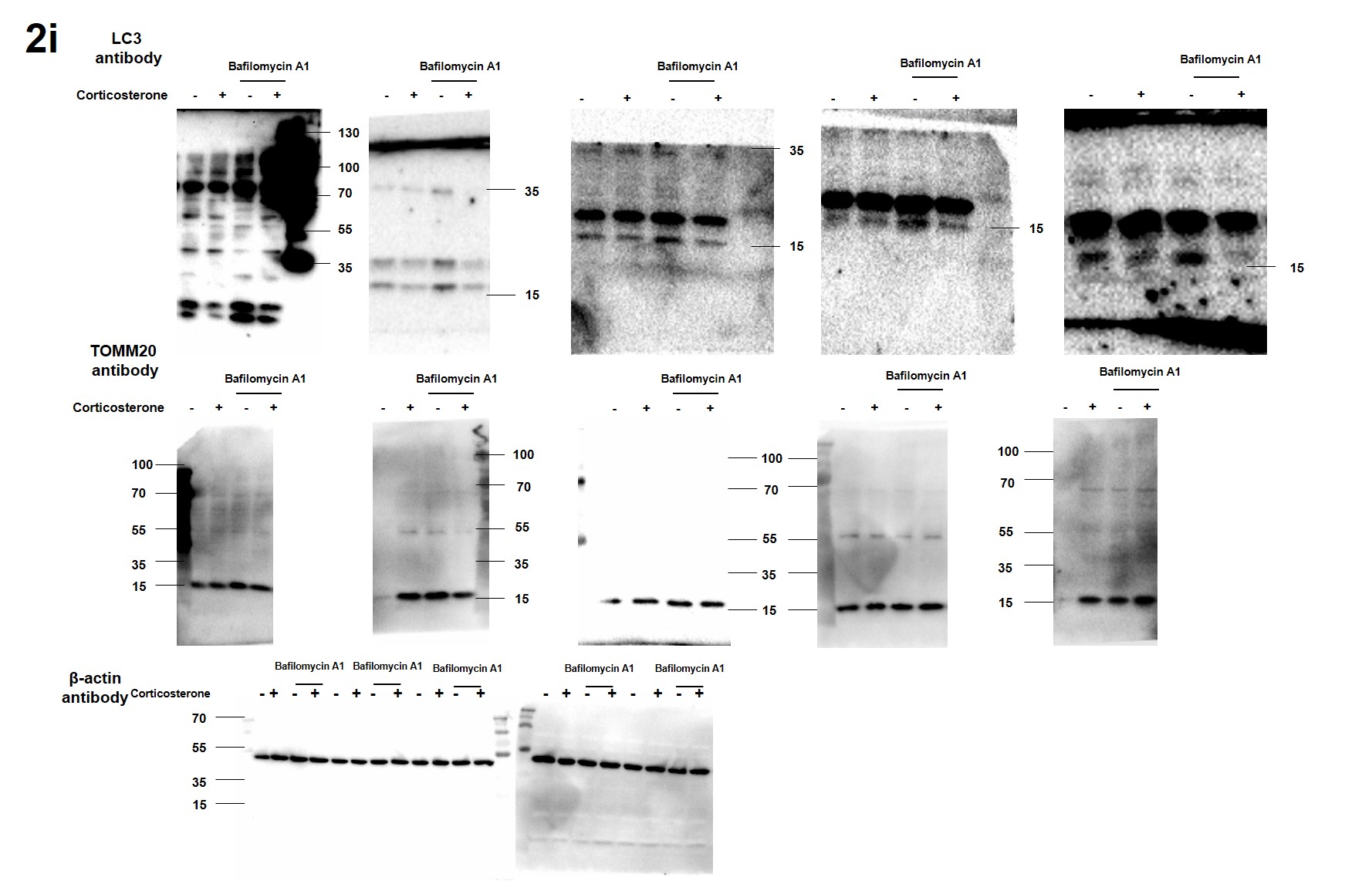

Supplement: Supplementary file 4 — Source Data [file 41467_2020_20679_MOESM4_ESM.zip › Fig 2/Fig 2i.jpg]

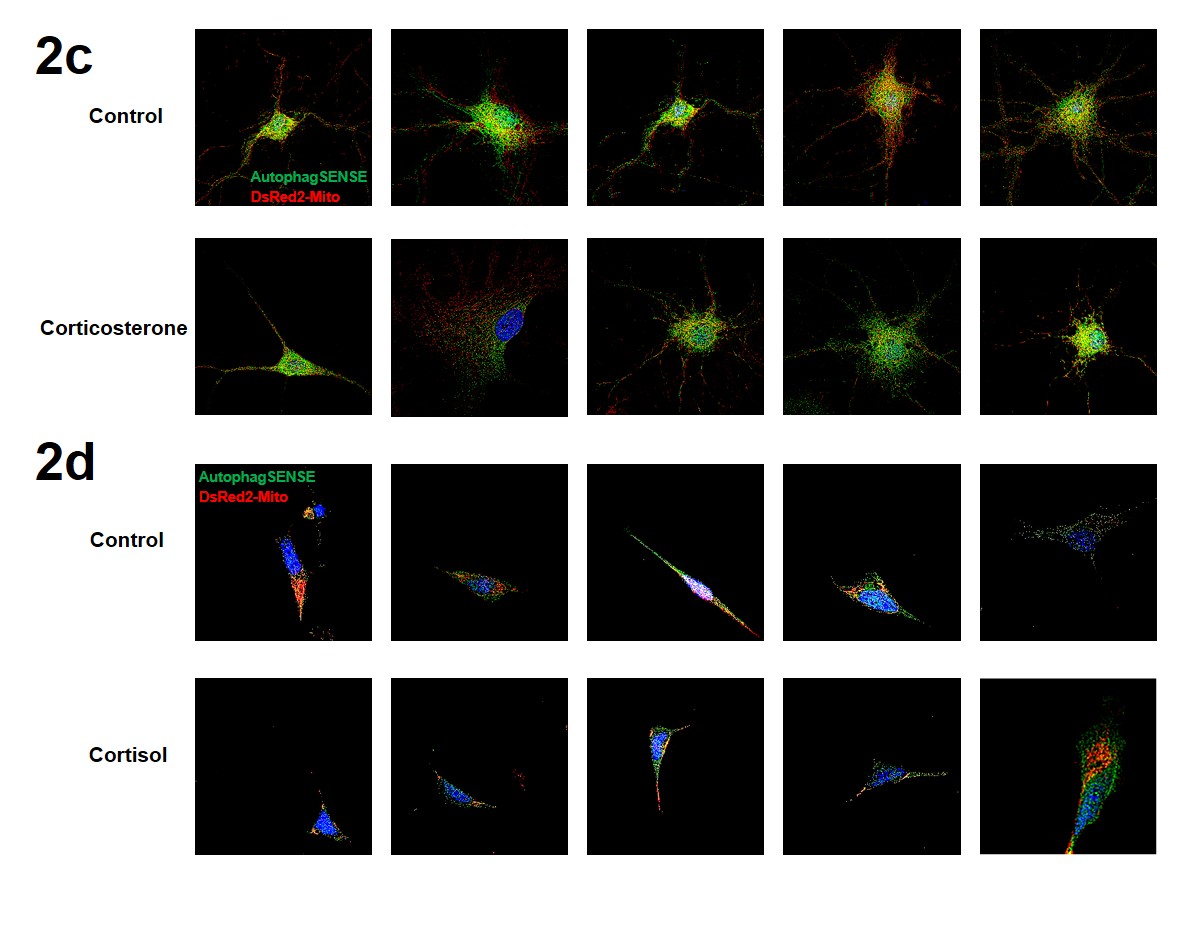

Supplement: Supplementary file 4 — Source Data [file 41467_2020_20679_MOESM4_ESM.zip › Fig 2/Figs 2c-2d.jpg]

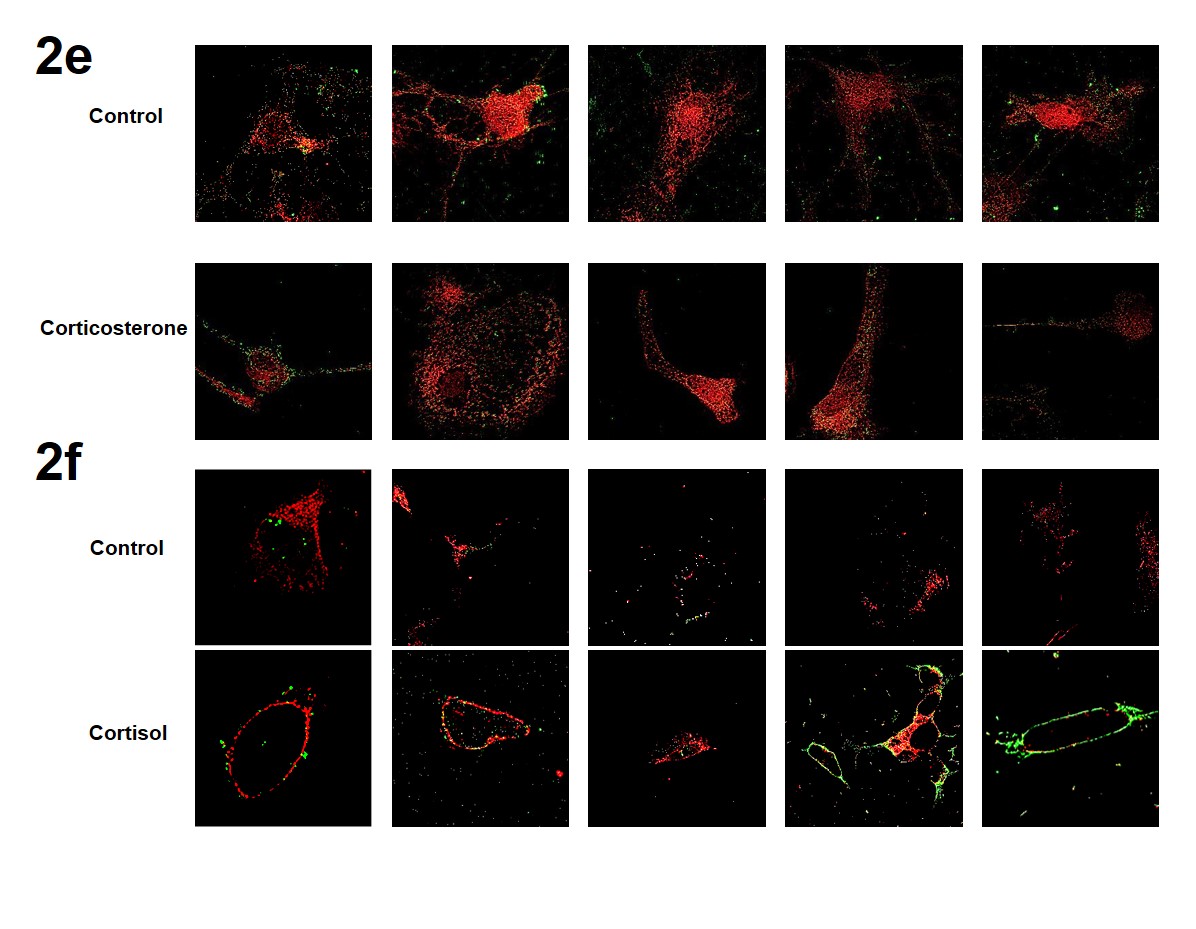

Supplement: Supplementary file 4 — Source Data [file 41467_2020_20679_MOESM4_ESM.zip › Fig 2/Figs 2e-2f.jpg]

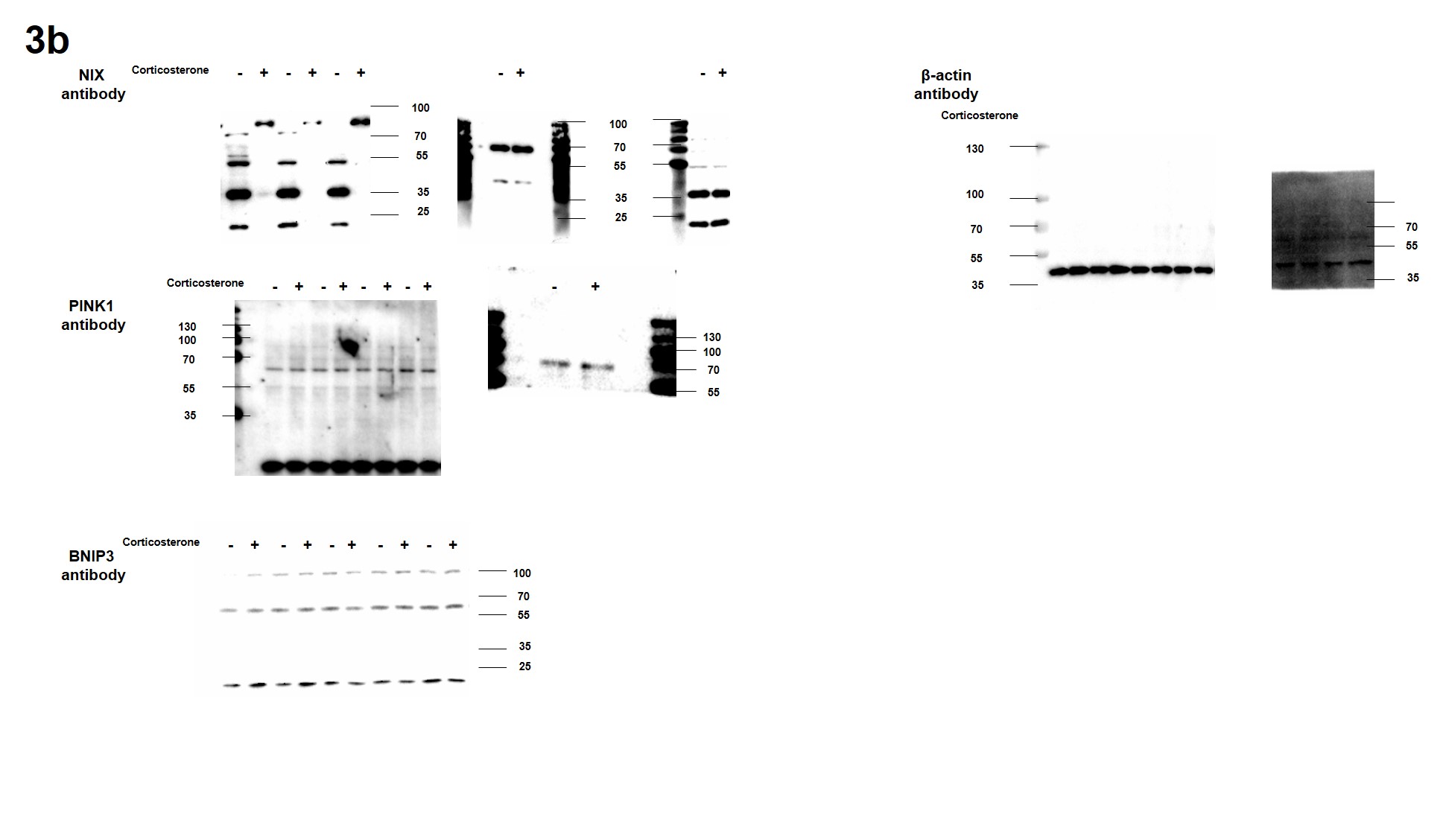

Supplement: Supplementary file 4 — Source Data [file 41467_2020_20679_MOESM4_ESM.zip › Fig 3/Fig 3b.jpg]

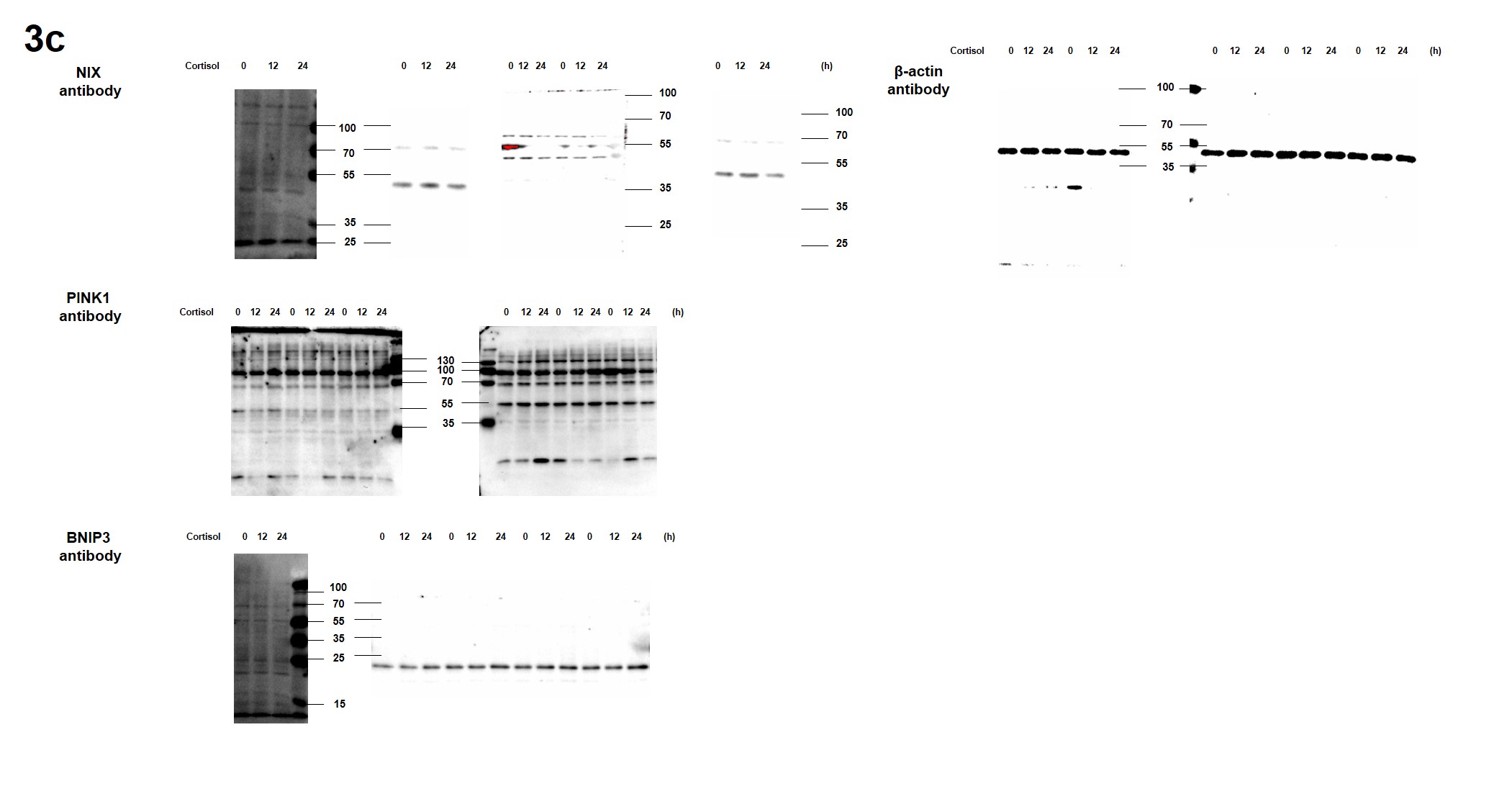

Supplement: Supplementary file 4 — Source Data [file 41467_2020_20679_MOESM4_ESM.zip › Fig 3/Fig 3c.jpg]

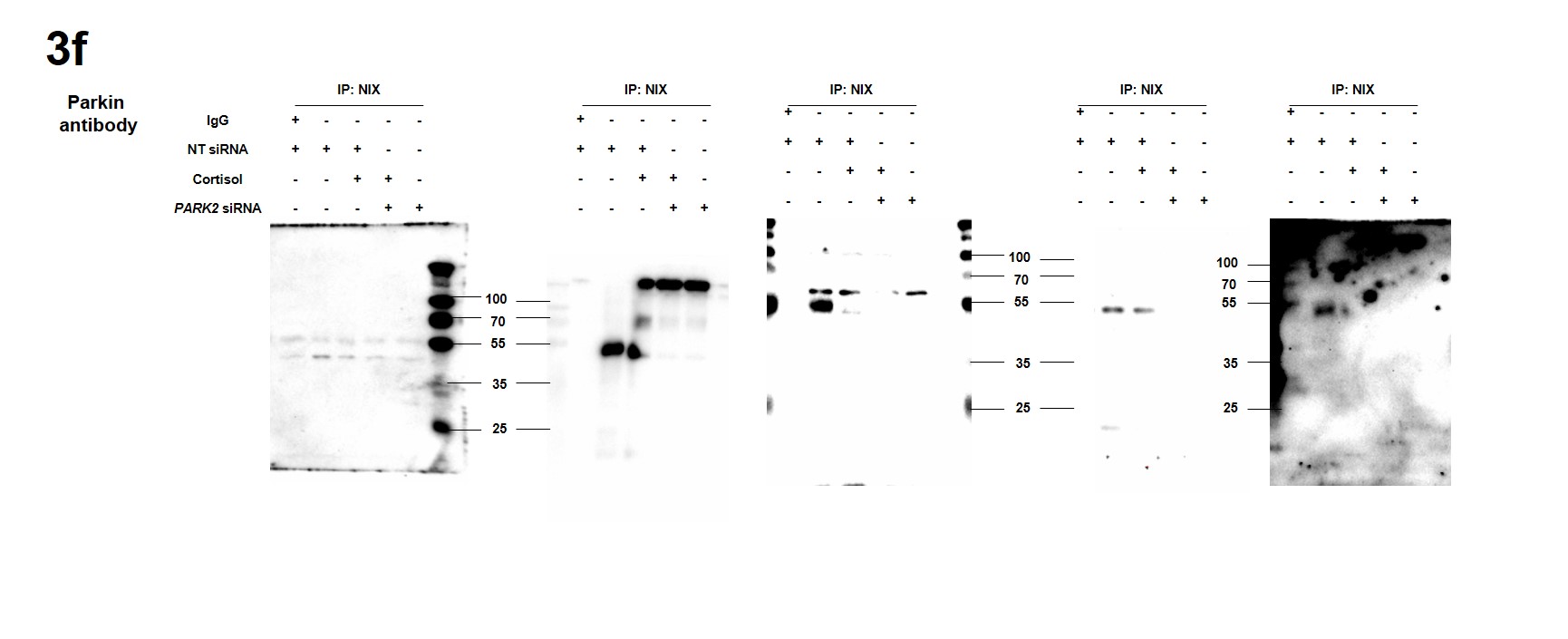

Supplement: Supplementary file 4 — Source Data [file 41467_2020_20679_MOESM4_ESM.zip › Fig 3/Fig 3f.jpg]

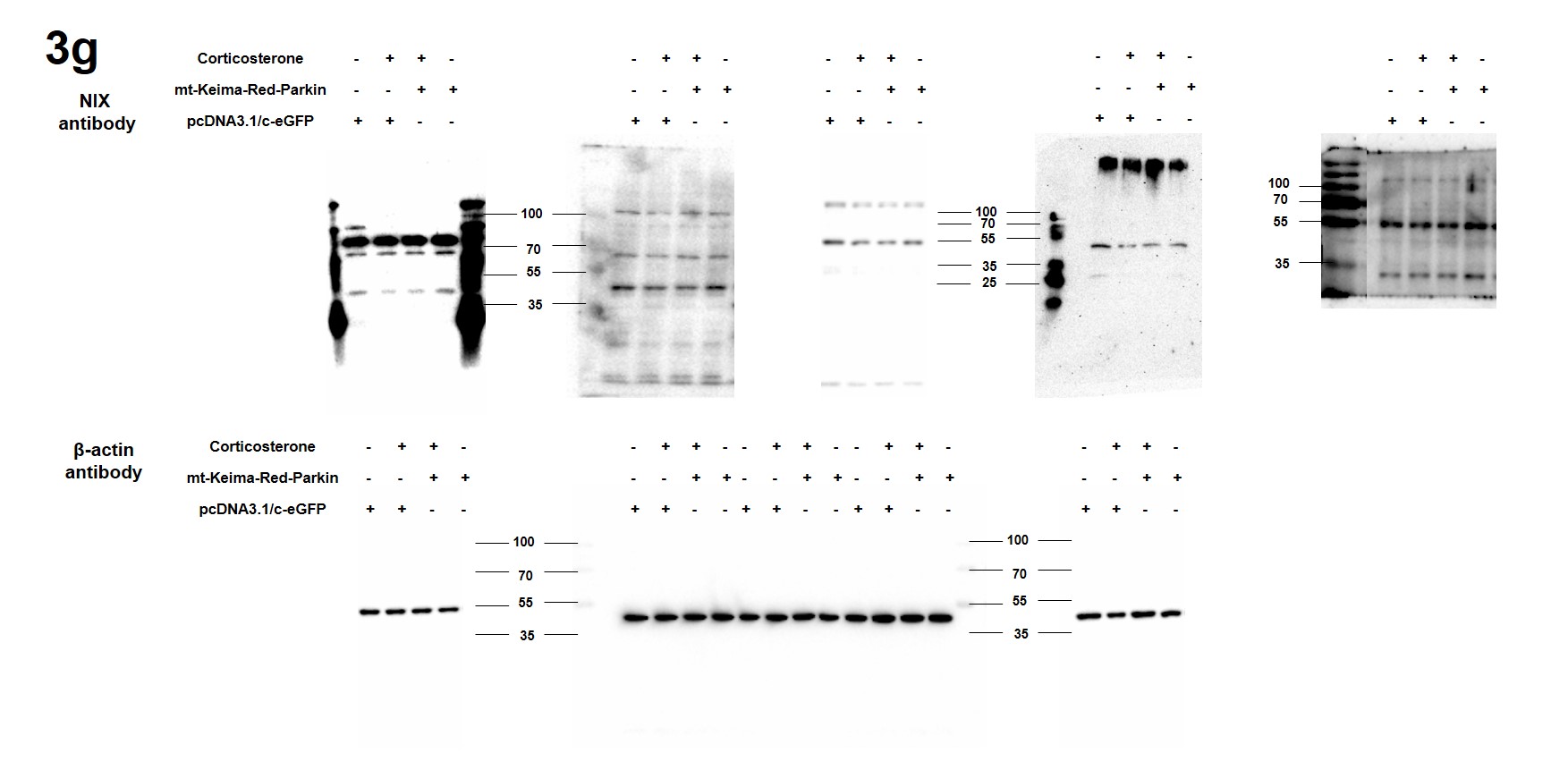

Supplement: Supplementary file 4 — Source Data [file 41467_2020_20679_MOESM4_ESM.zip › Fig 3/Fig 3g.jpg]

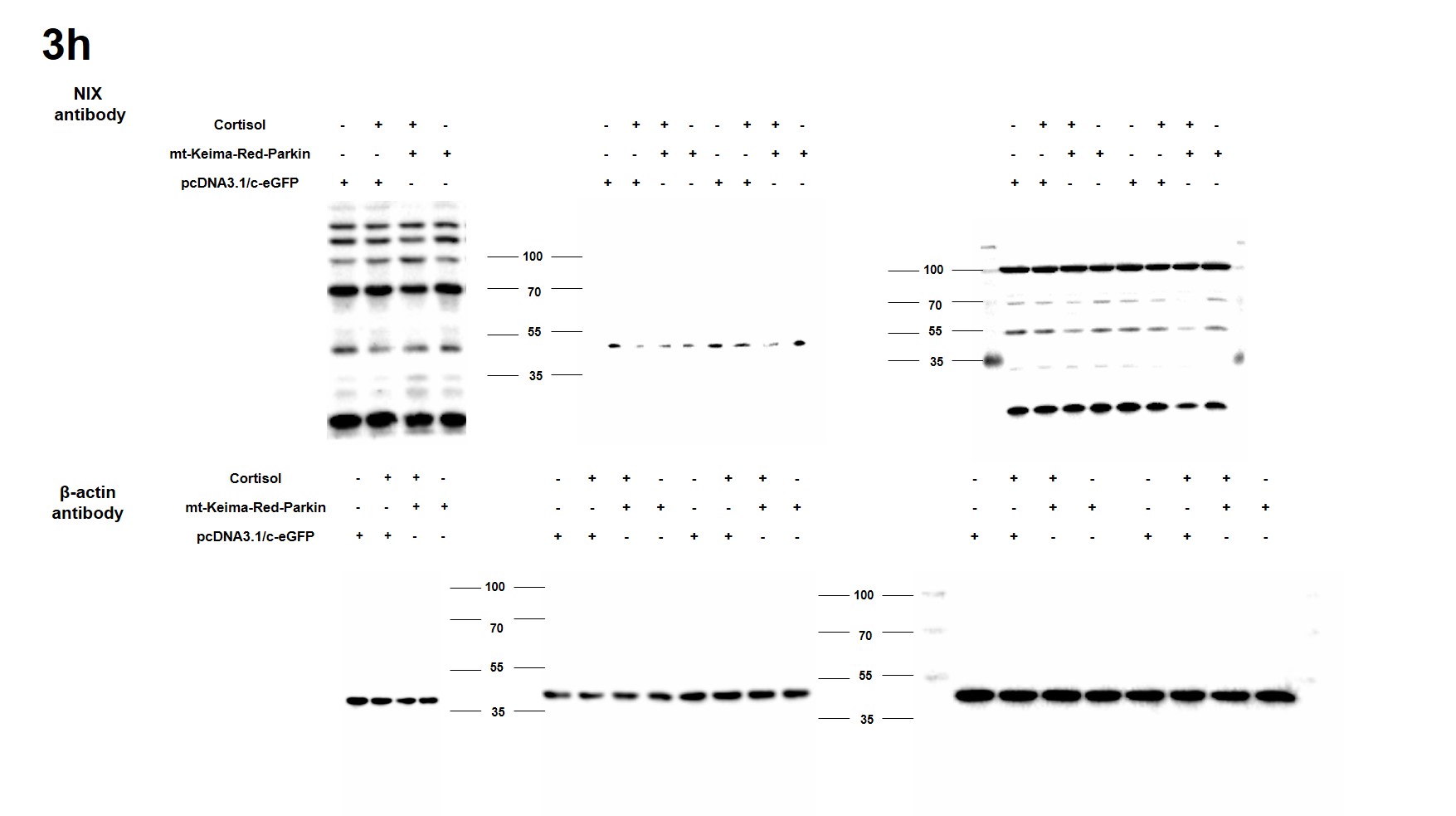

Supplement: Supplementary file 4 — Source Data [file 41467_2020_20679_MOESM4_ESM.zip › Fig 3/Fig 3h.jpg]

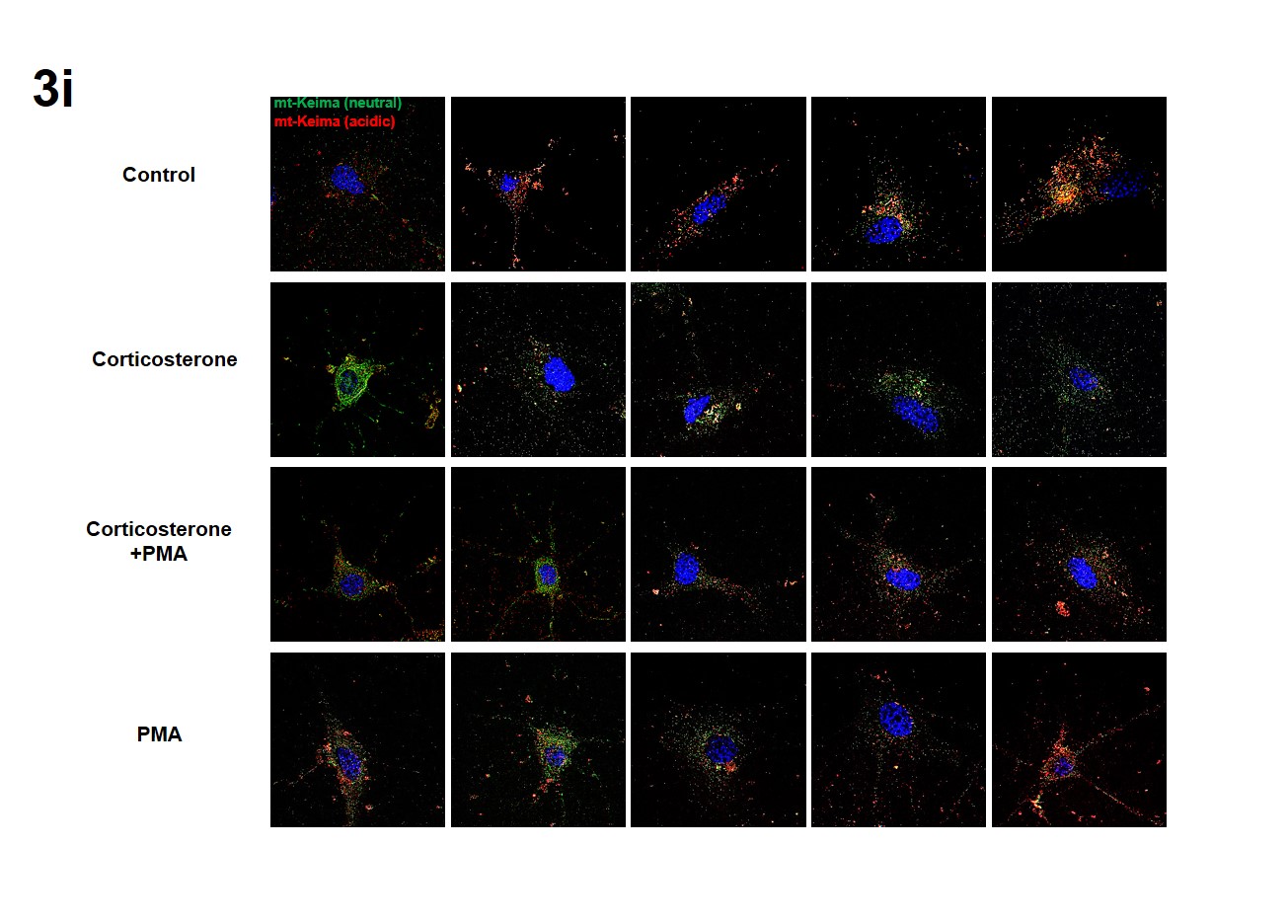

Supplement: Supplementary file 4 — Source Data [file 41467_2020_20679_MOESM4_ESM.zip › Fig 3/Fig 3i.jpg]

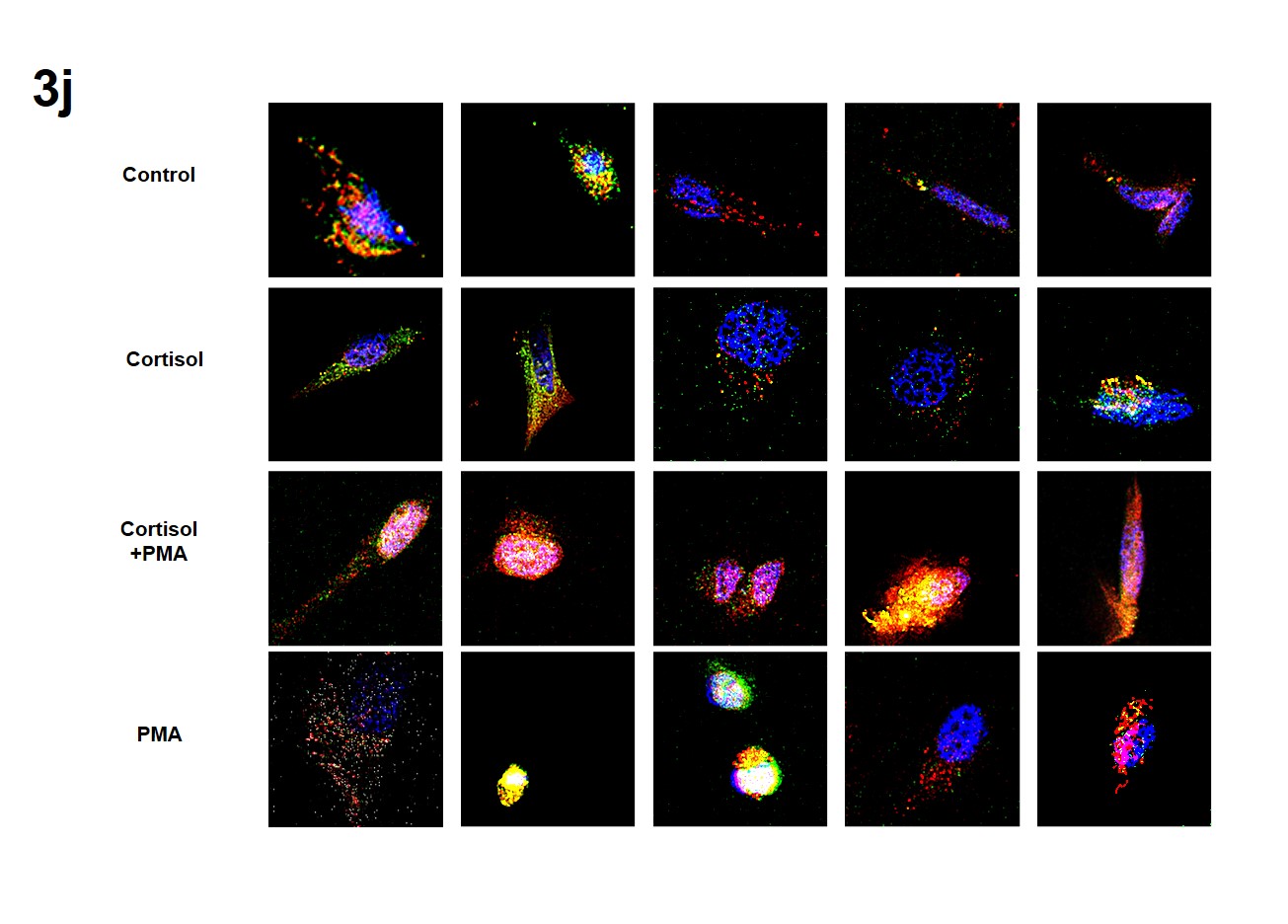

Supplement: Supplementary file 4 — Source Data [file 41467_2020_20679_MOESM4_ESM.zip › Fig 3/Fig 3j.jpg]

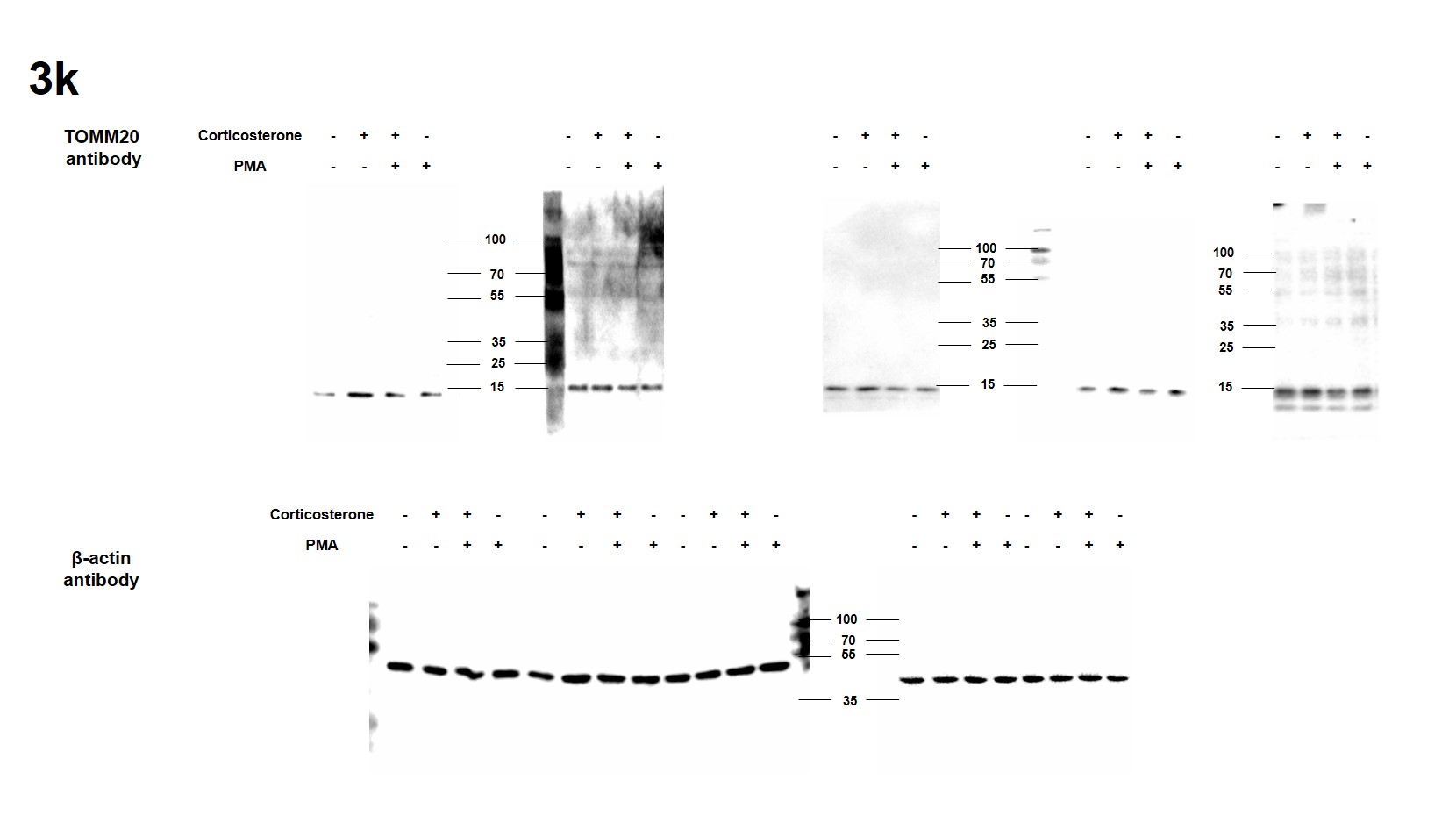

Supplement: Supplementary file 4 — Source Data [file 41467_2020_20679_MOESM4_ESM.zip › Fig 3/Fig 3k.jpg]

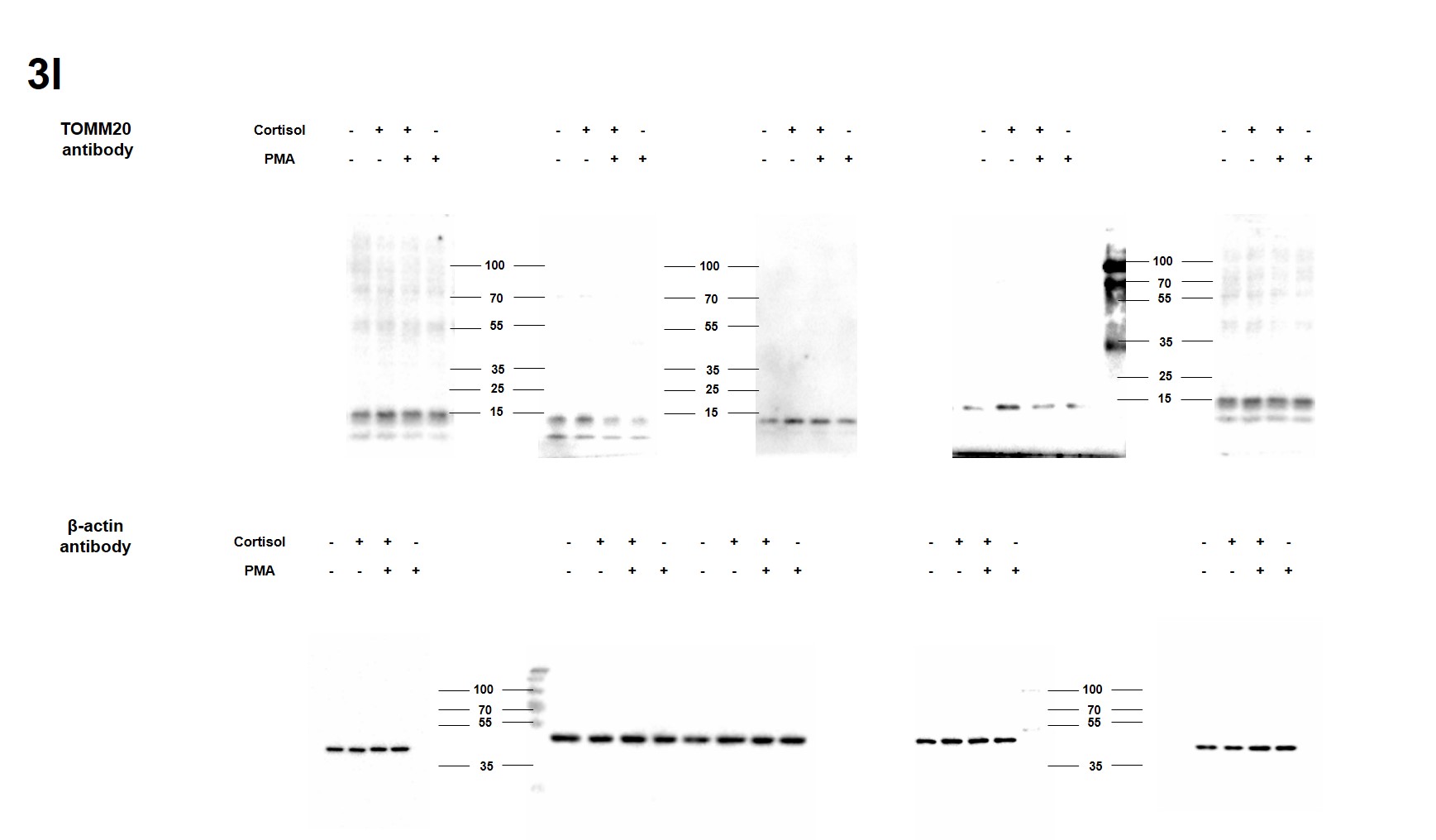

Supplement: Supplementary file 4 — Source Data [file 41467_2020_20679_MOESM4_ESM.zip › Fig 3/Fig 3l.jpg]

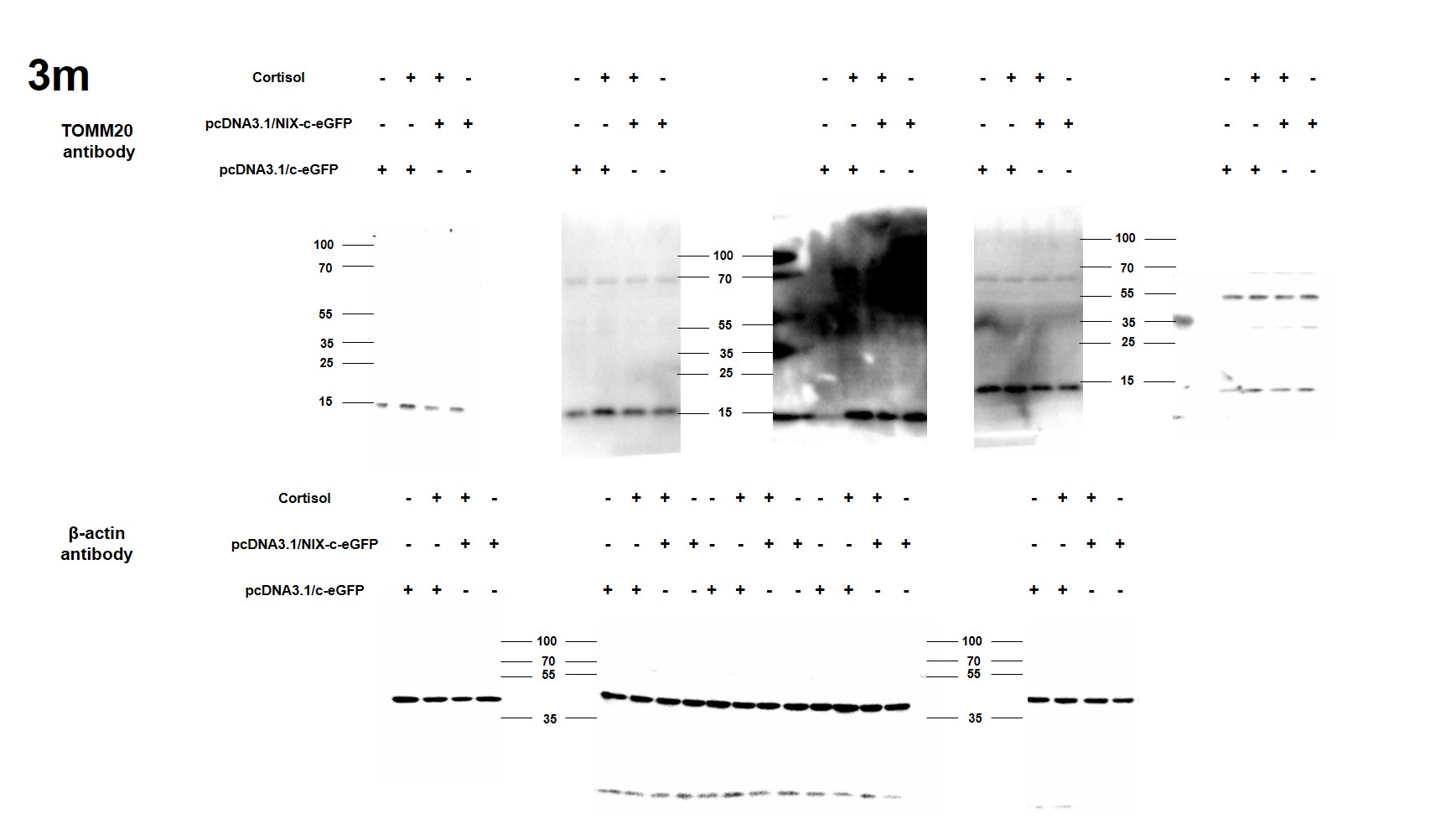

Supplement: Supplementary file 4 — Source Data [file 41467_2020_20679_MOESM4_ESM.zip › Fig 3/Fig 3m.jpg]

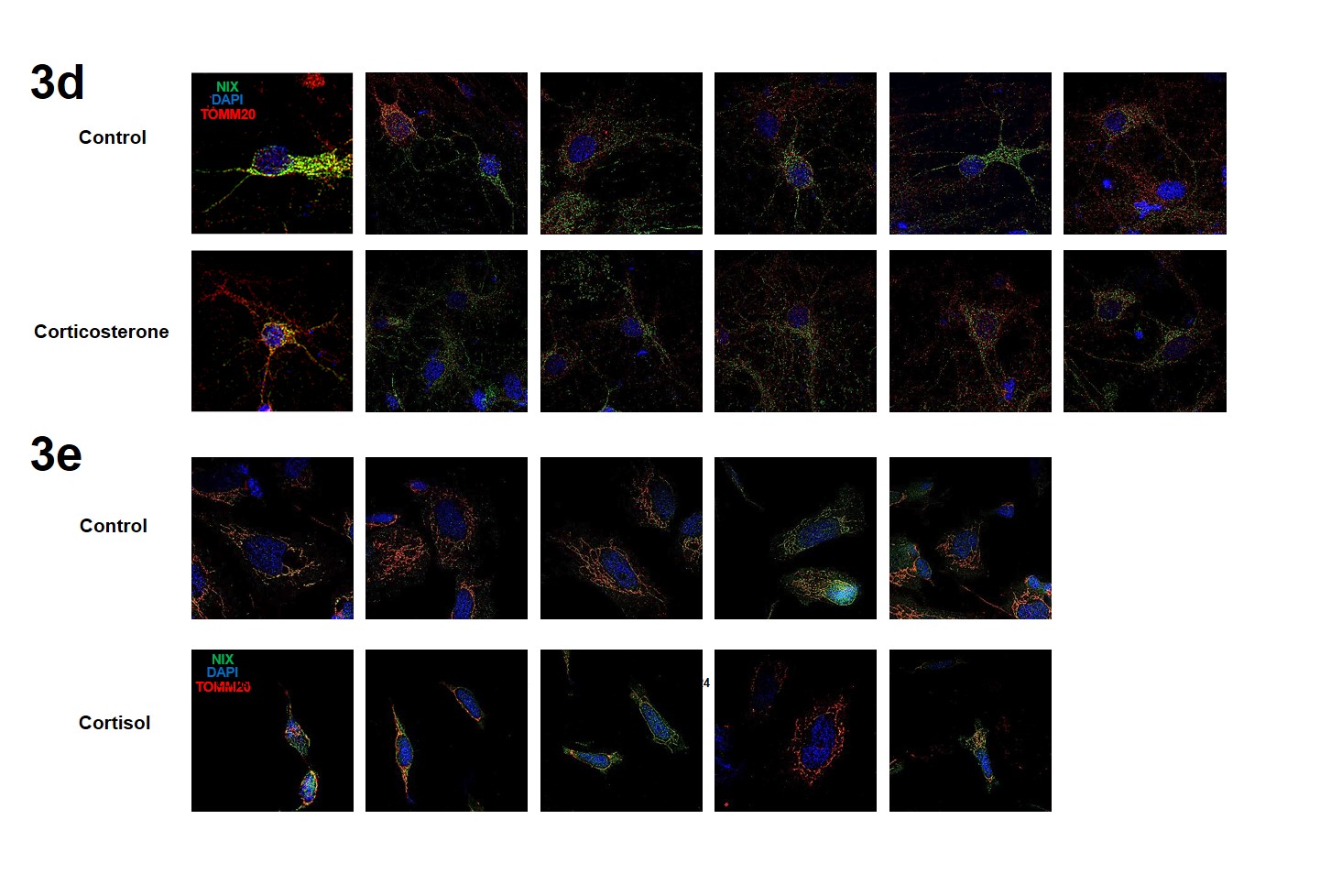

Supplement: Supplementary file 4 — Source Data [file 41467_2020_20679_MOESM4_ESM.zip › Fig 3/Figs 3d-3e.jpg]

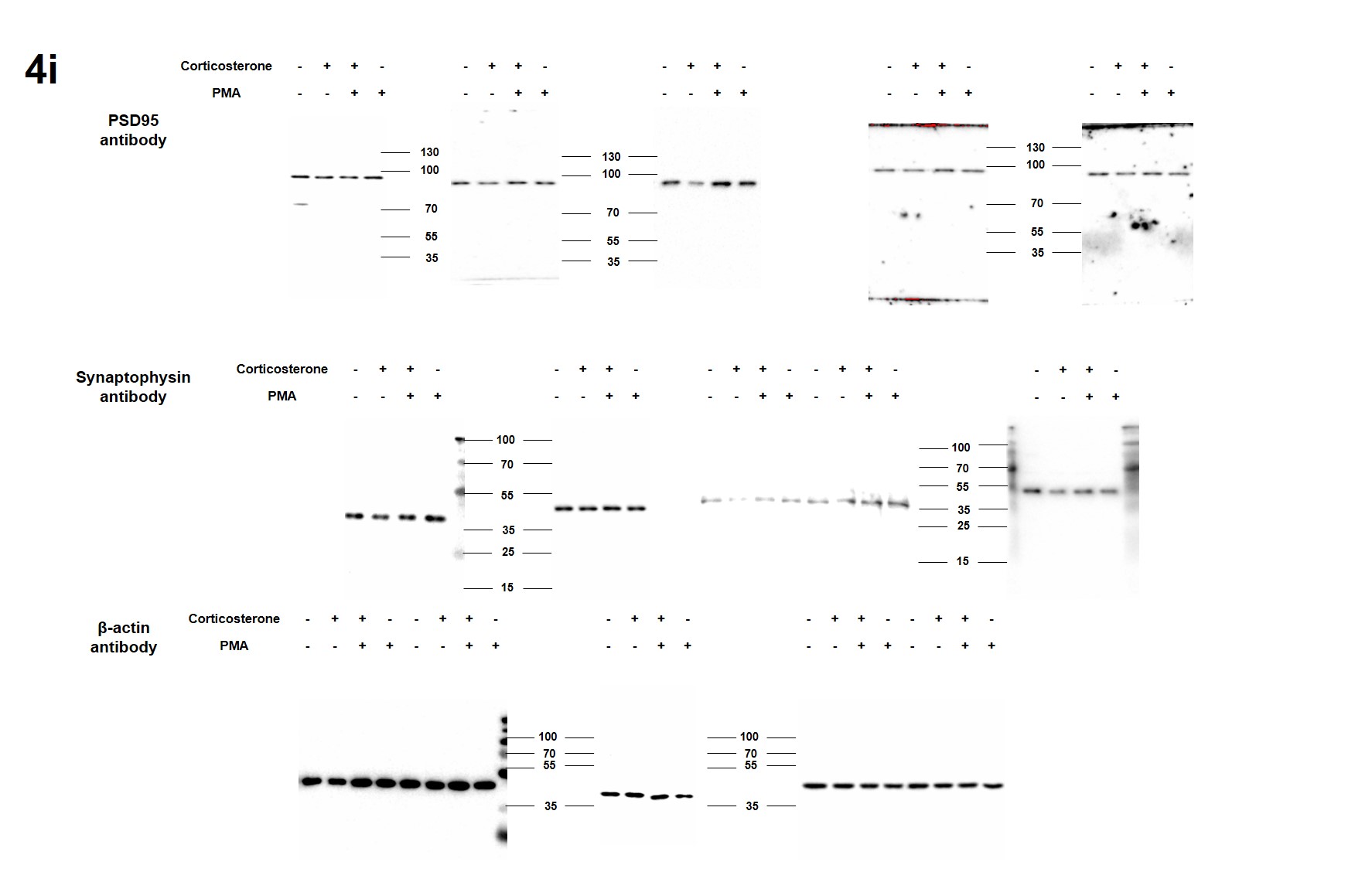

Supplement: Supplementary file 4 — Source Data [file 41467_2020_20679_MOESM4_ESM.zip › Fig 4/Fig 4i.jpg]

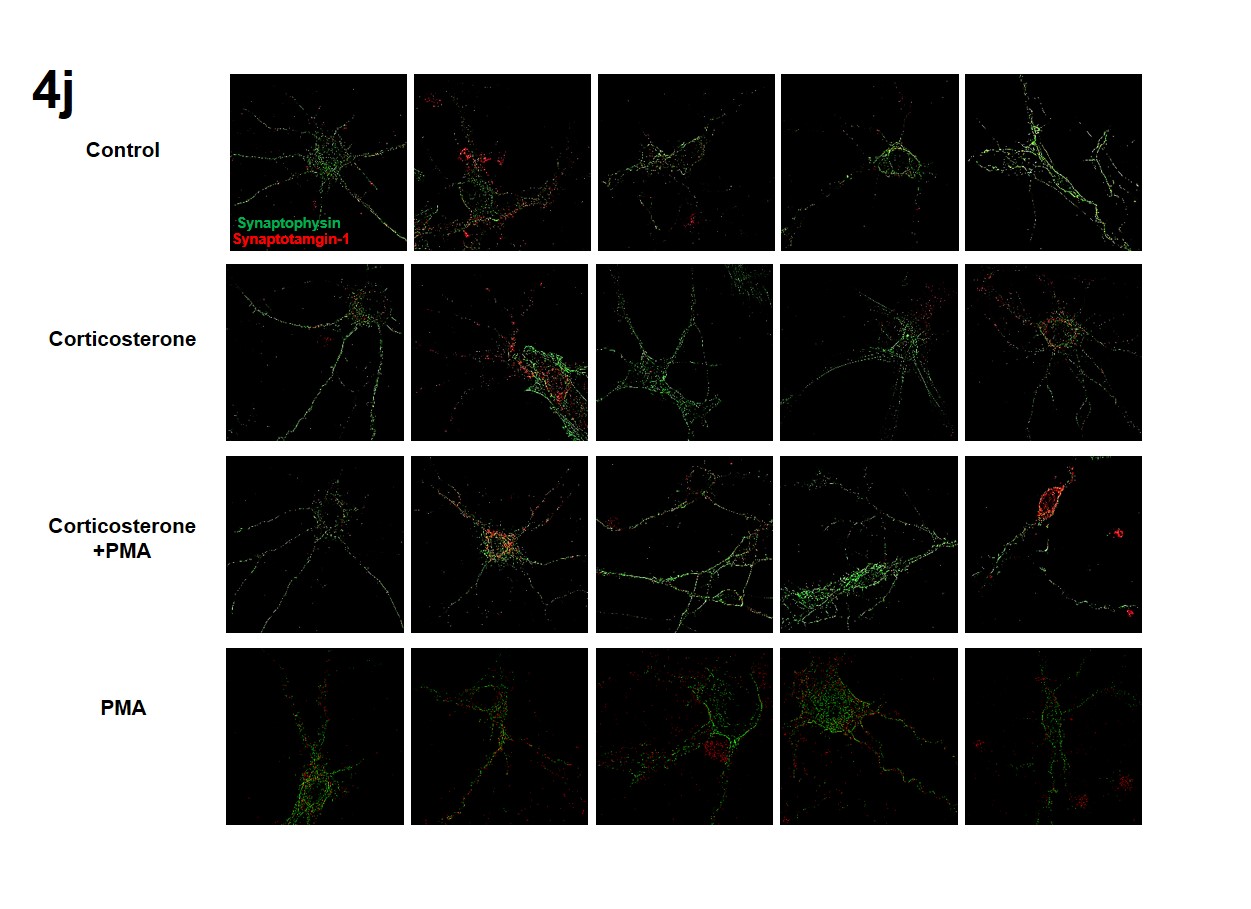

Supplement: Supplementary file 4 — Source Data [file 41467_2020_20679_MOESM4_ESM.zip › Fig 4/Fig 4j.jpg]

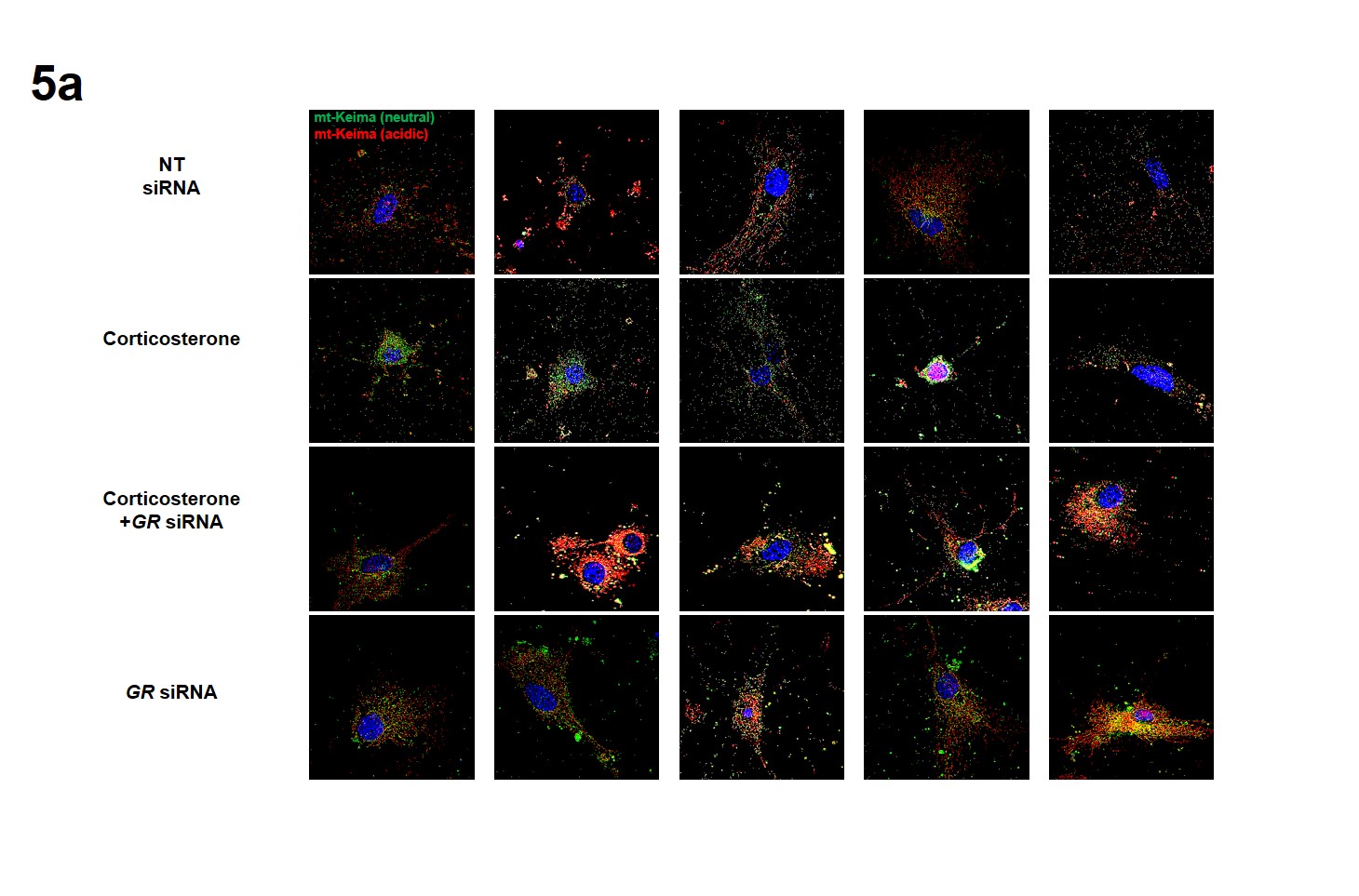

Supplement: Supplementary file 4 — Source Data [file 41467_2020_20679_MOESM4_ESM.zip › Fig 5/Fig 5a.jpg]

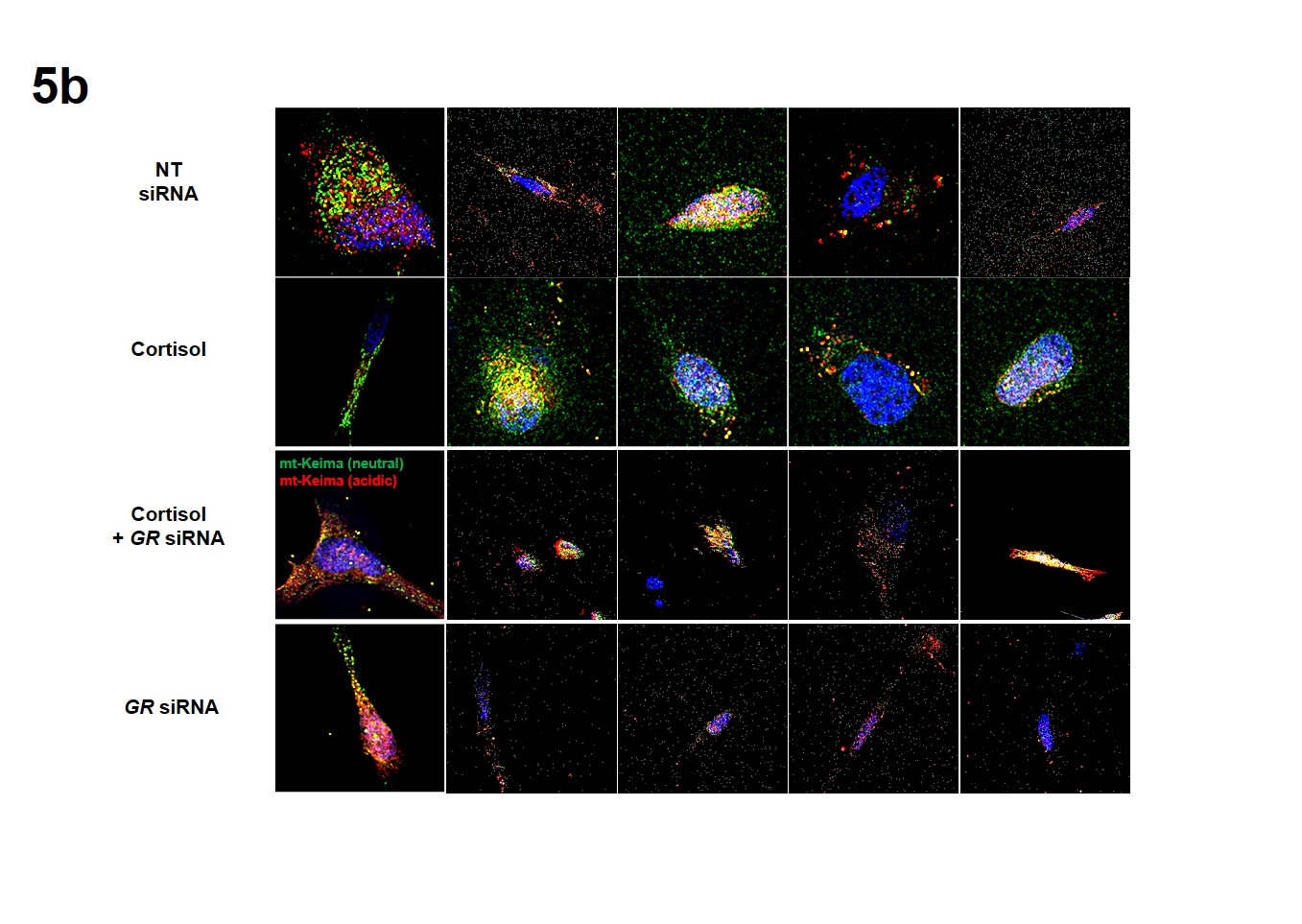

Supplement: Supplementary file 4 — Source Data [file 41467_2020_20679_MOESM4_ESM.zip › Fig 5/Fig 5b.jpg]

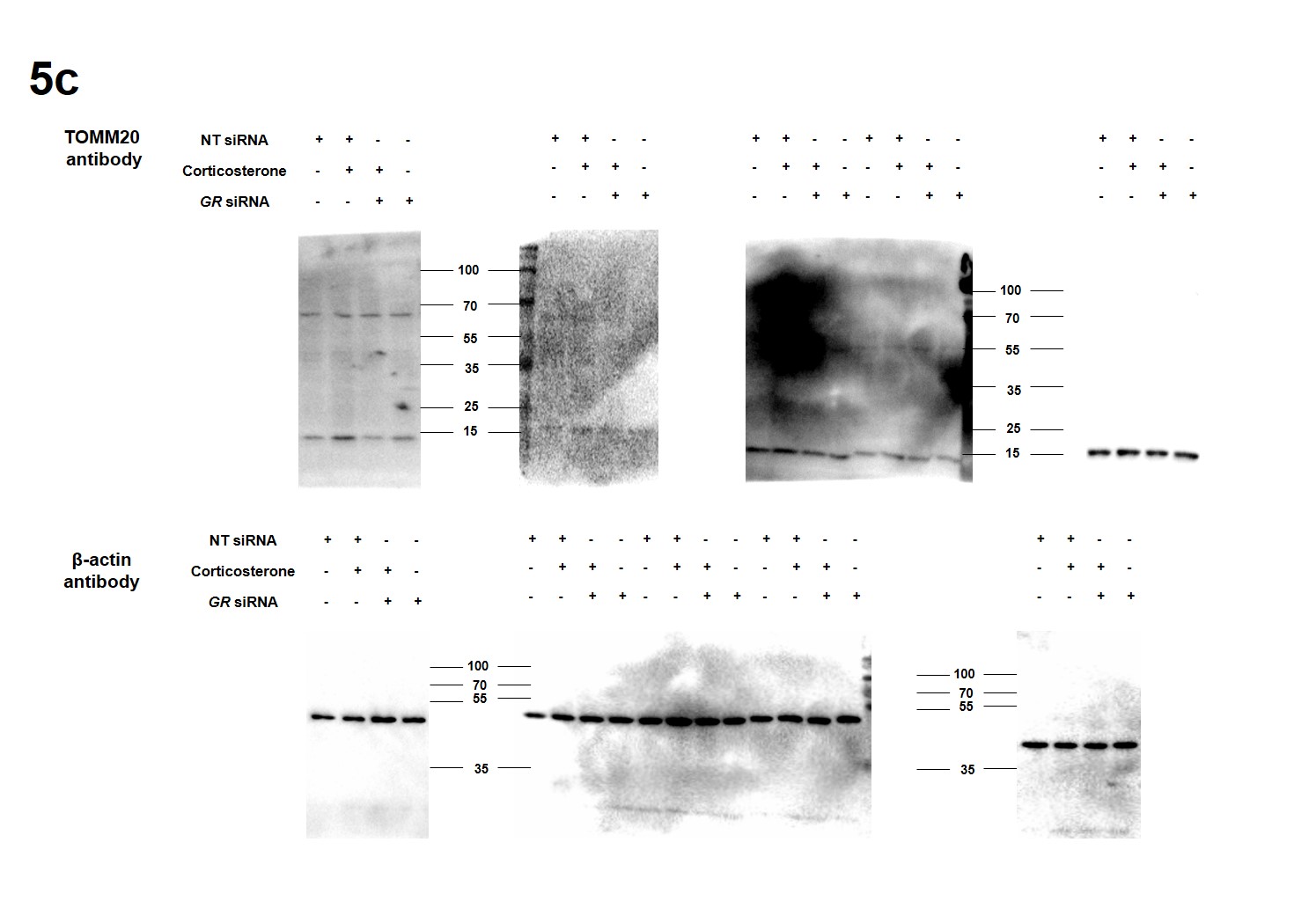

Supplement: Supplementary file 4 — Source Data [file 41467_2020_20679_MOESM4_ESM.zip › Fig 5/Fig 5c.jpg]

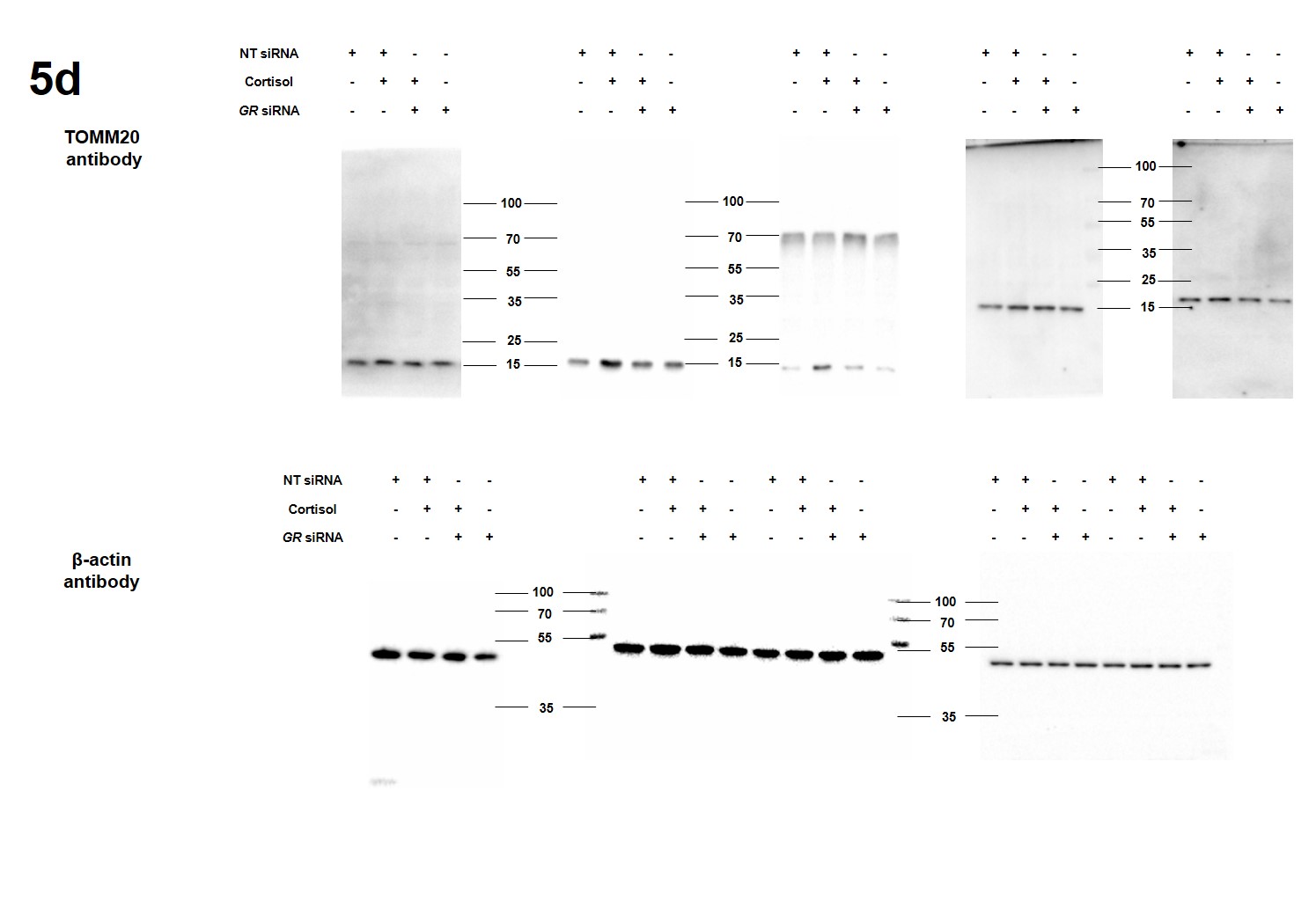

Supplement: Supplementary file 4 — Source Data [file 41467_2020_20679_MOESM4_ESM.zip › Fig 5/Fig 5d.jpg]

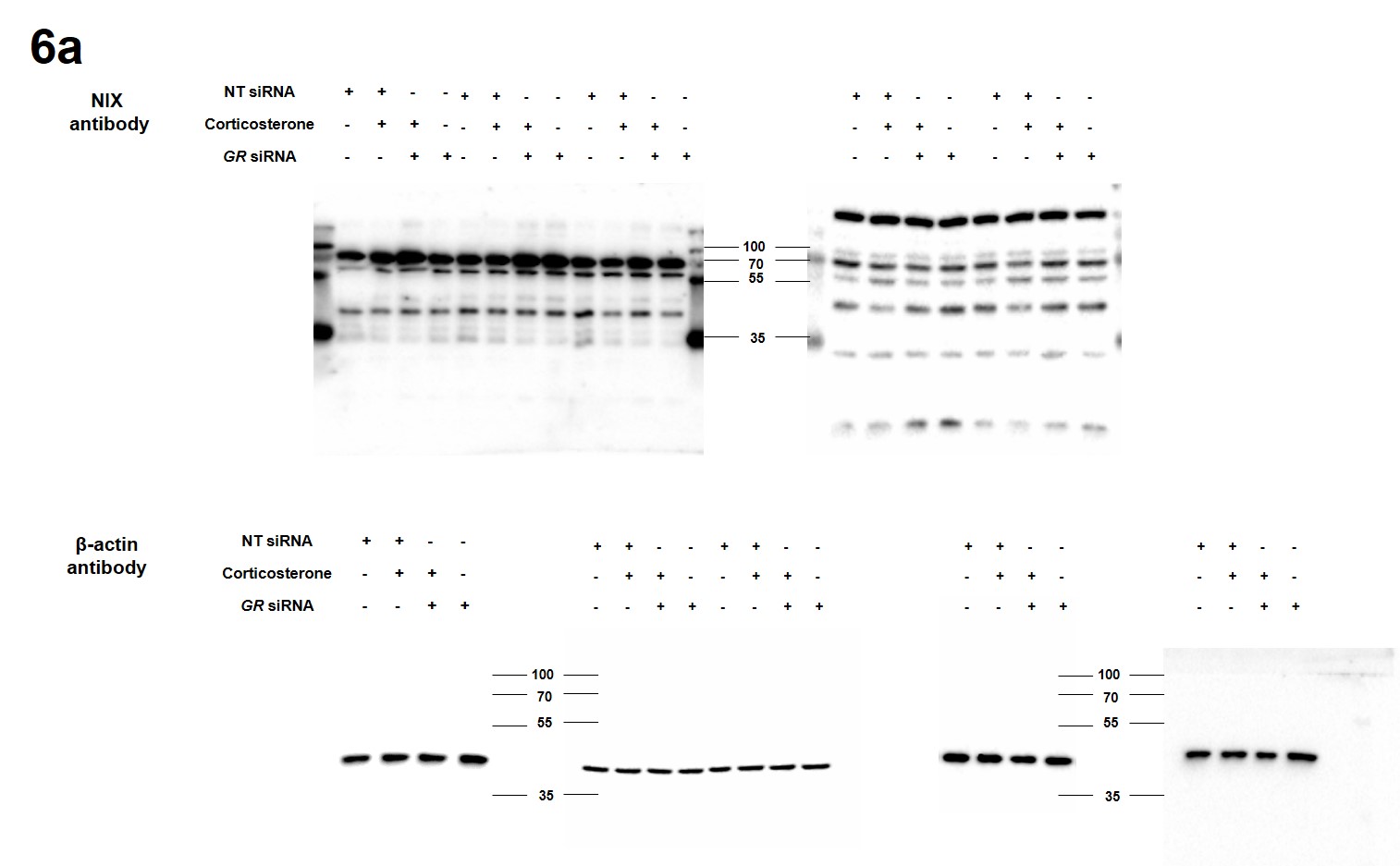

Supplement: Supplementary file 4 — Source Data [file 41467_2020_20679_MOESM4_ESM.zip › Fig 6/Fig 6a.jpg]

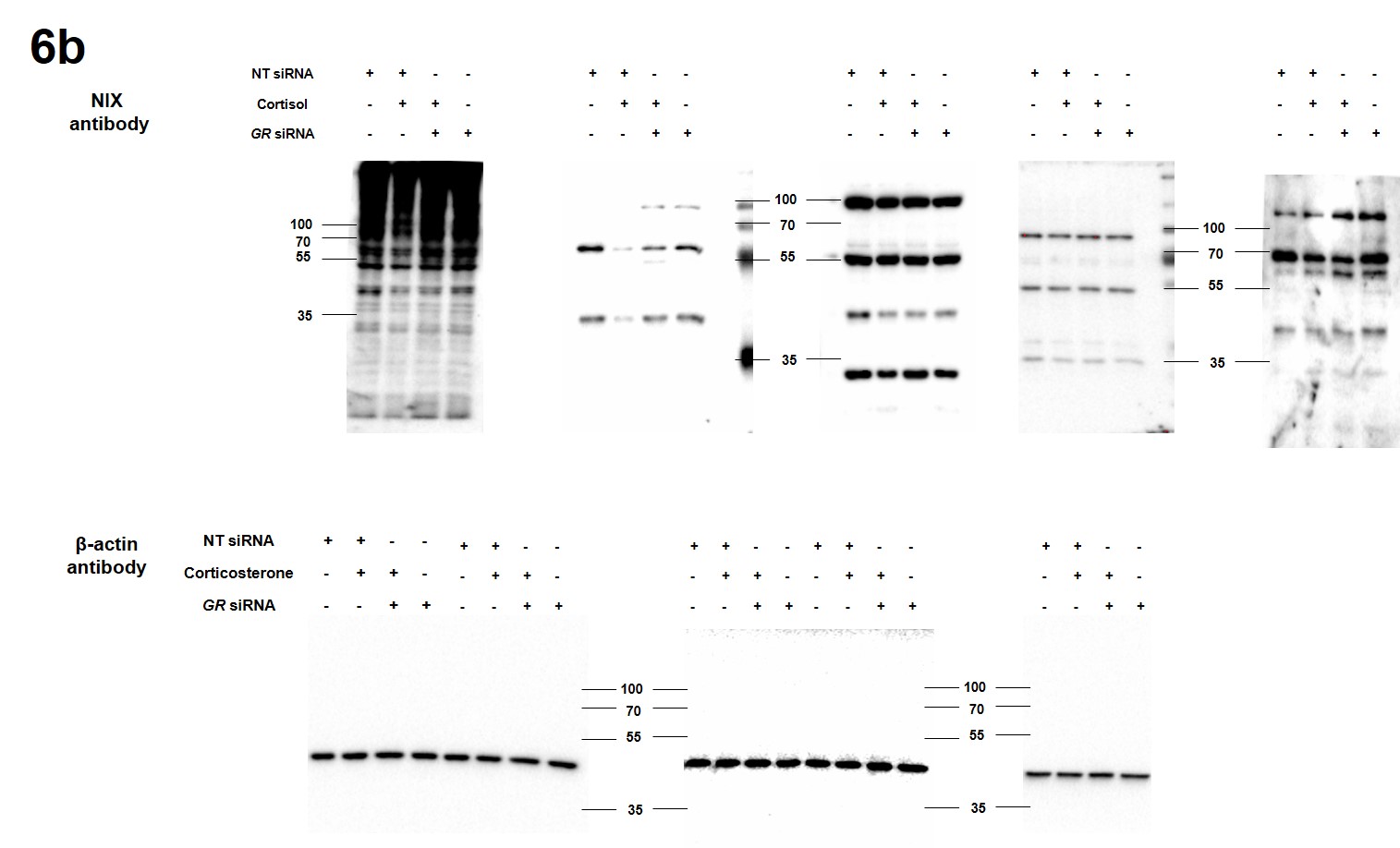

Supplement: Supplementary file 4 — Source Data [file 41467_2020_20679_MOESM4_ESM.zip › Fig 6/Fig 6b.jpg]

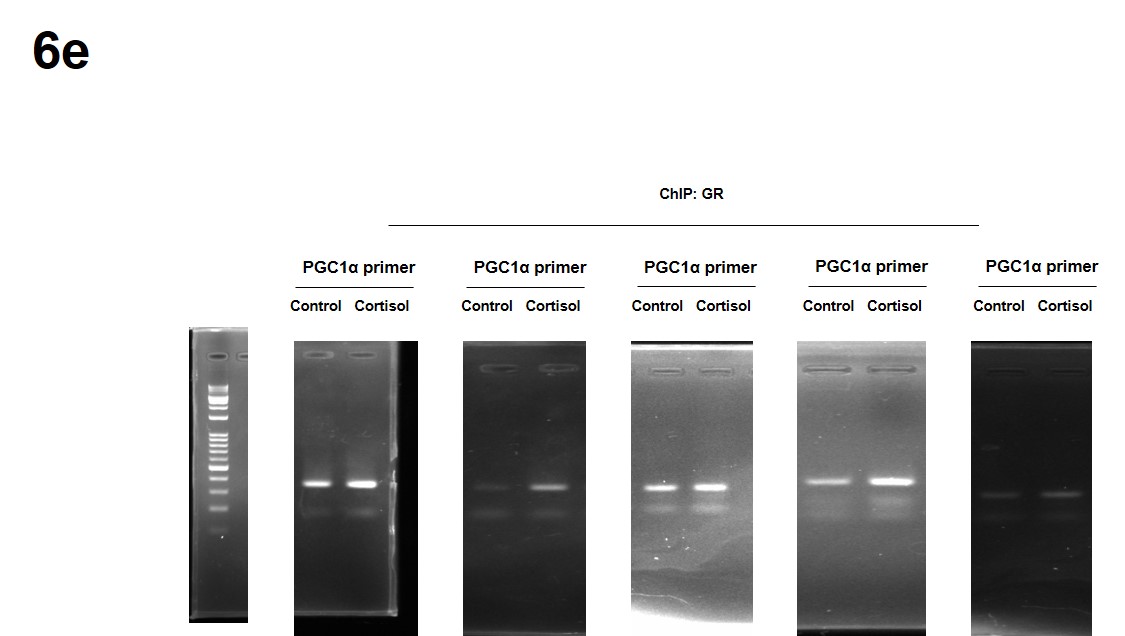

Supplement: Supplementary file 4 — Source Data [file 41467_2020_20679_MOESM4_ESM.zip › Fig 6/Fig 6e.jpg]

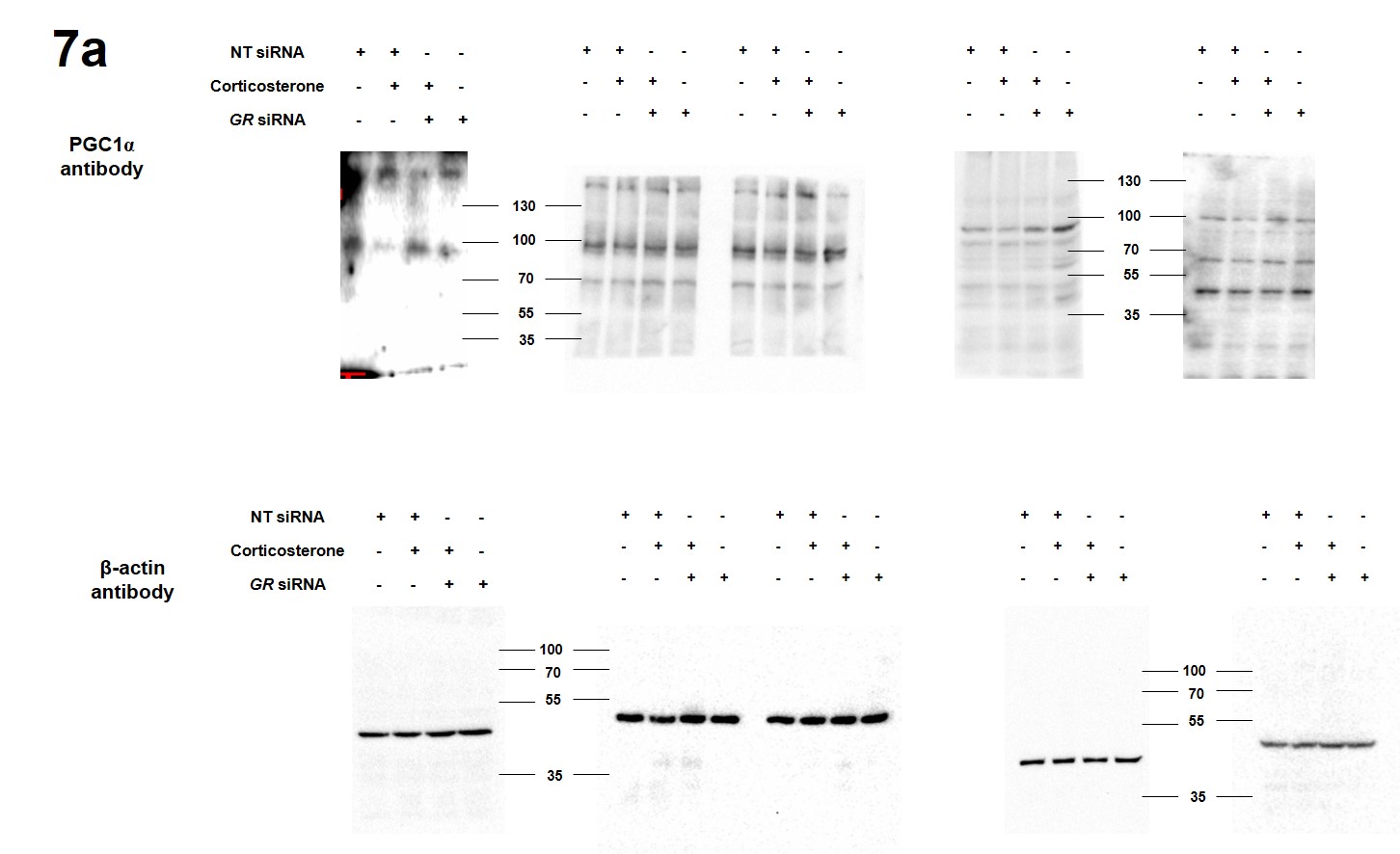

Supplement: Supplementary file 4 — Source Data [file 41467_2020_20679_MOESM4_ESM.zip › Fig 7/Fig 7a.jpg]

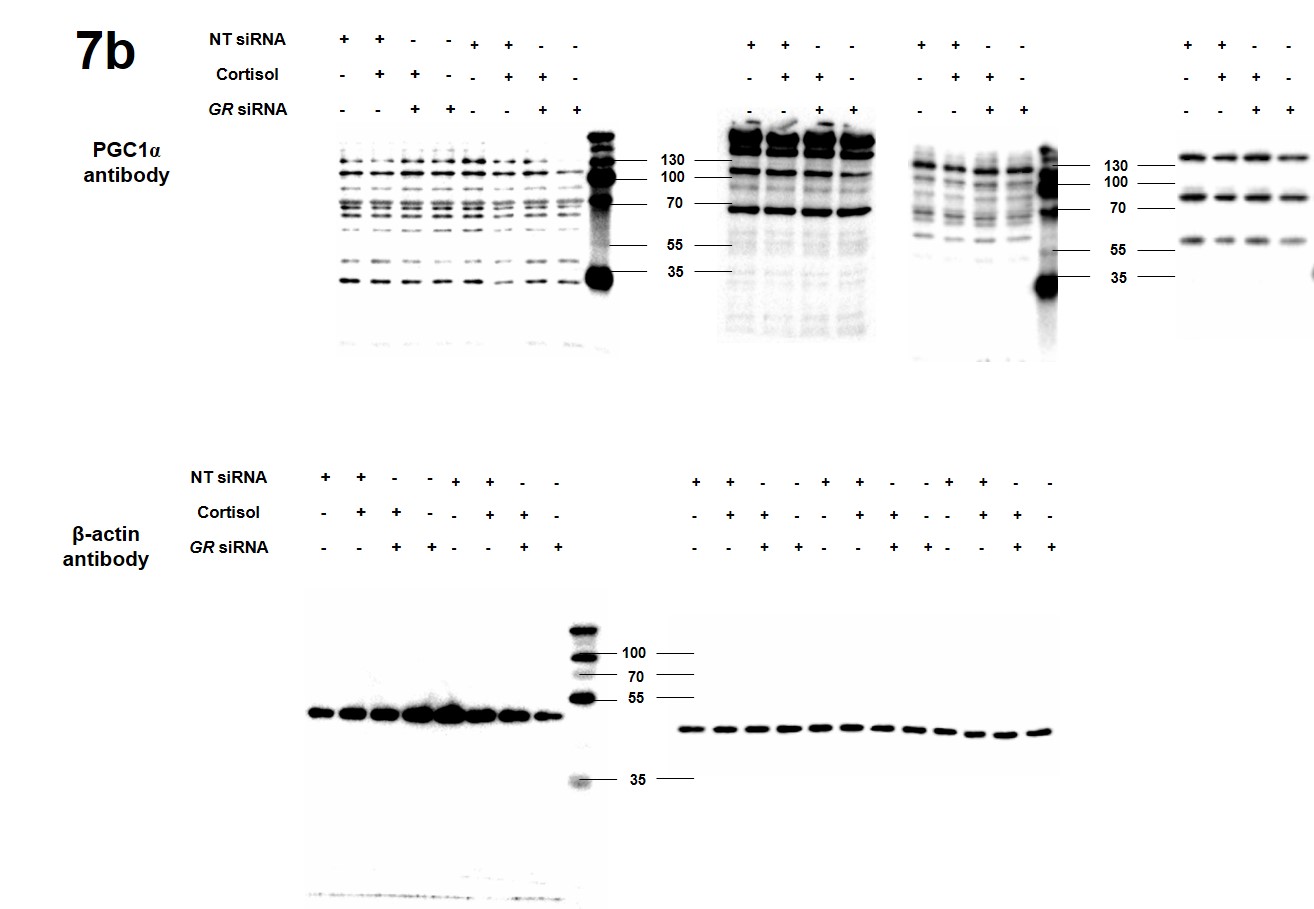

Supplement: Supplementary file 4 — Source Data [file 41467_2020_20679_MOESM4_ESM.zip › Fig 7/Fig 7b.jpg]

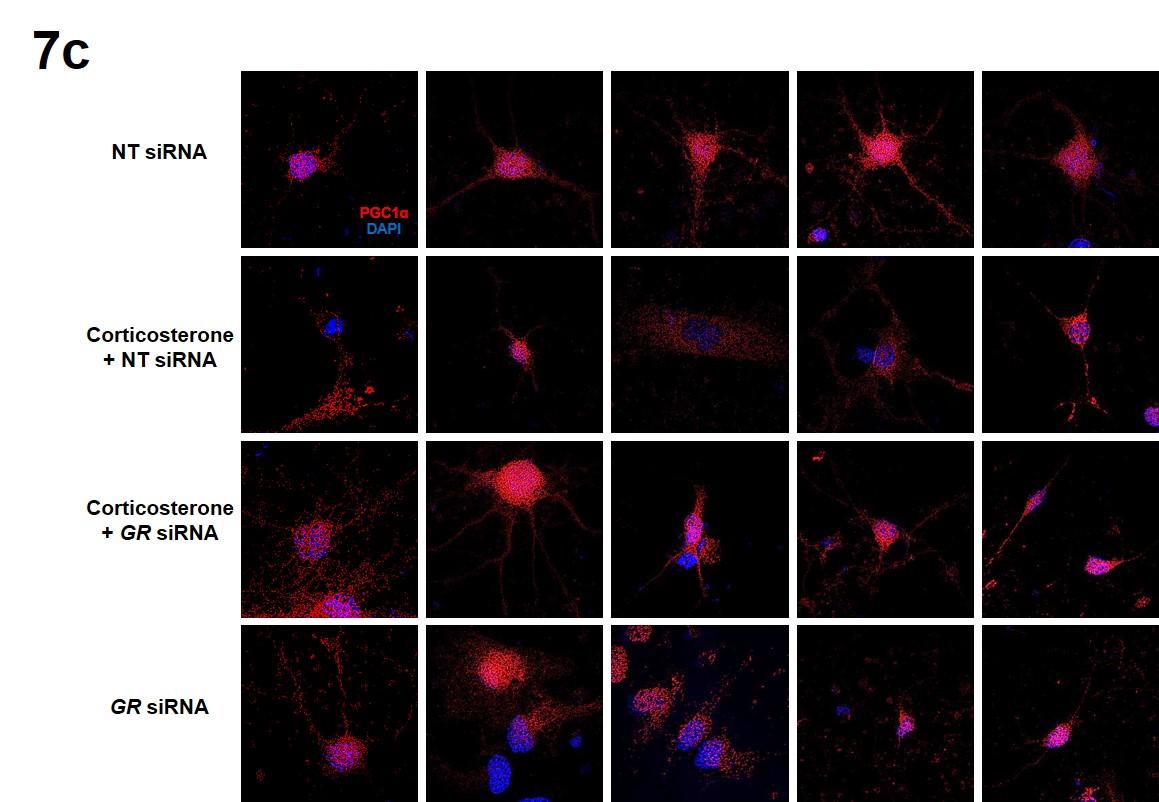

Supplement: Supplementary file 4 — Source Data [file 41467_2020_20679_MOESM4_ESM.zip › Fig 7/Fig 7c.jpg]

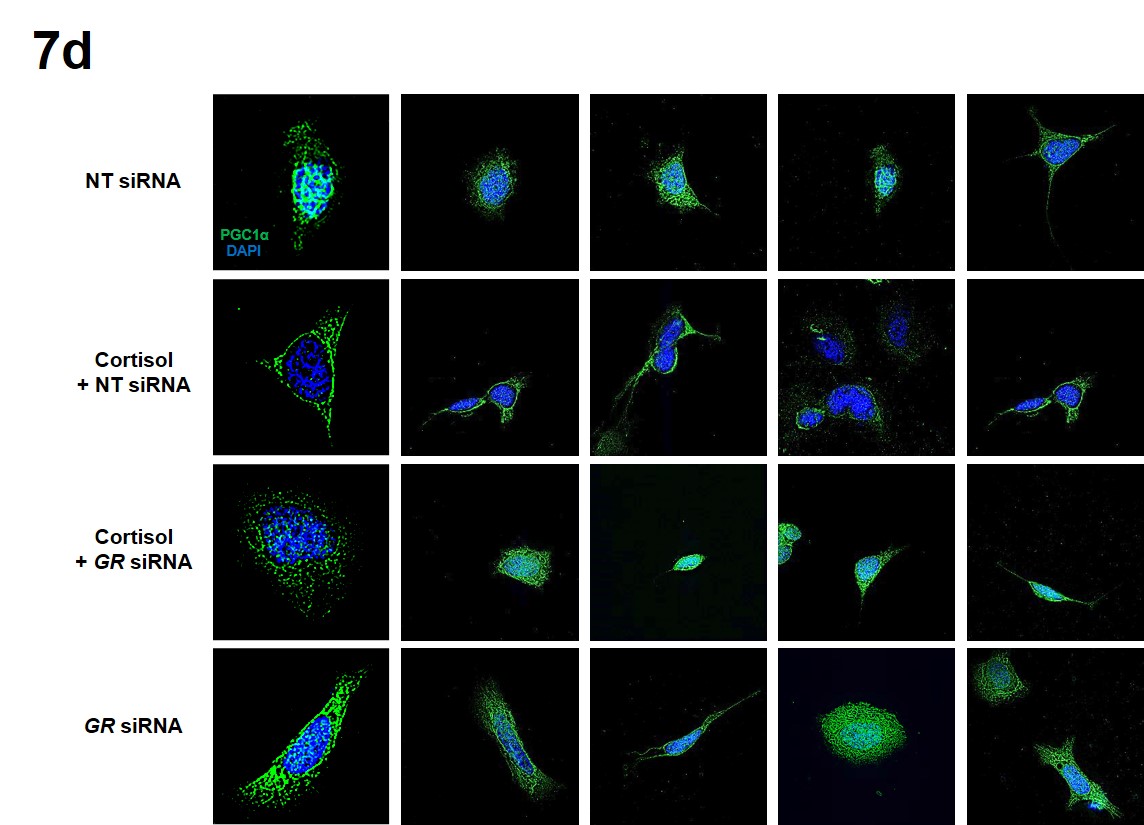

Supplement: Supplementary file 4 — Source Data [file 41467_2020_20679_MOESM4_ESM.zip › Fig 7/Fig 7d.jpg]

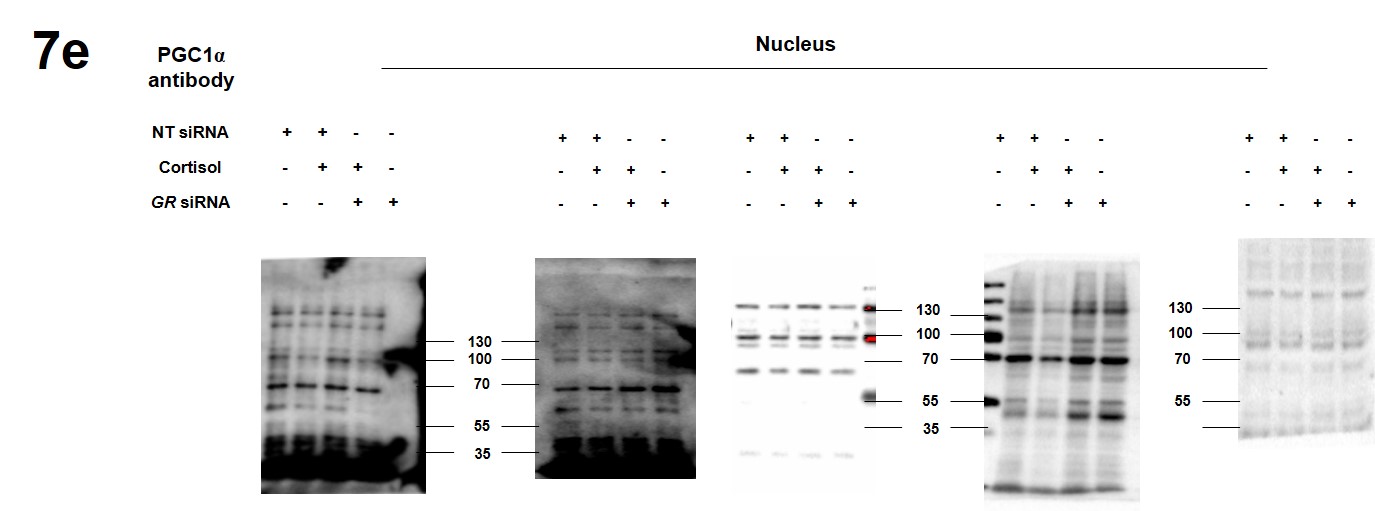

Supplement: Supplementary file 4 — Source Data [file 41467_2020_20679_MOESM4_ESM.zip › Fig 7/Fig 7e.jpg]

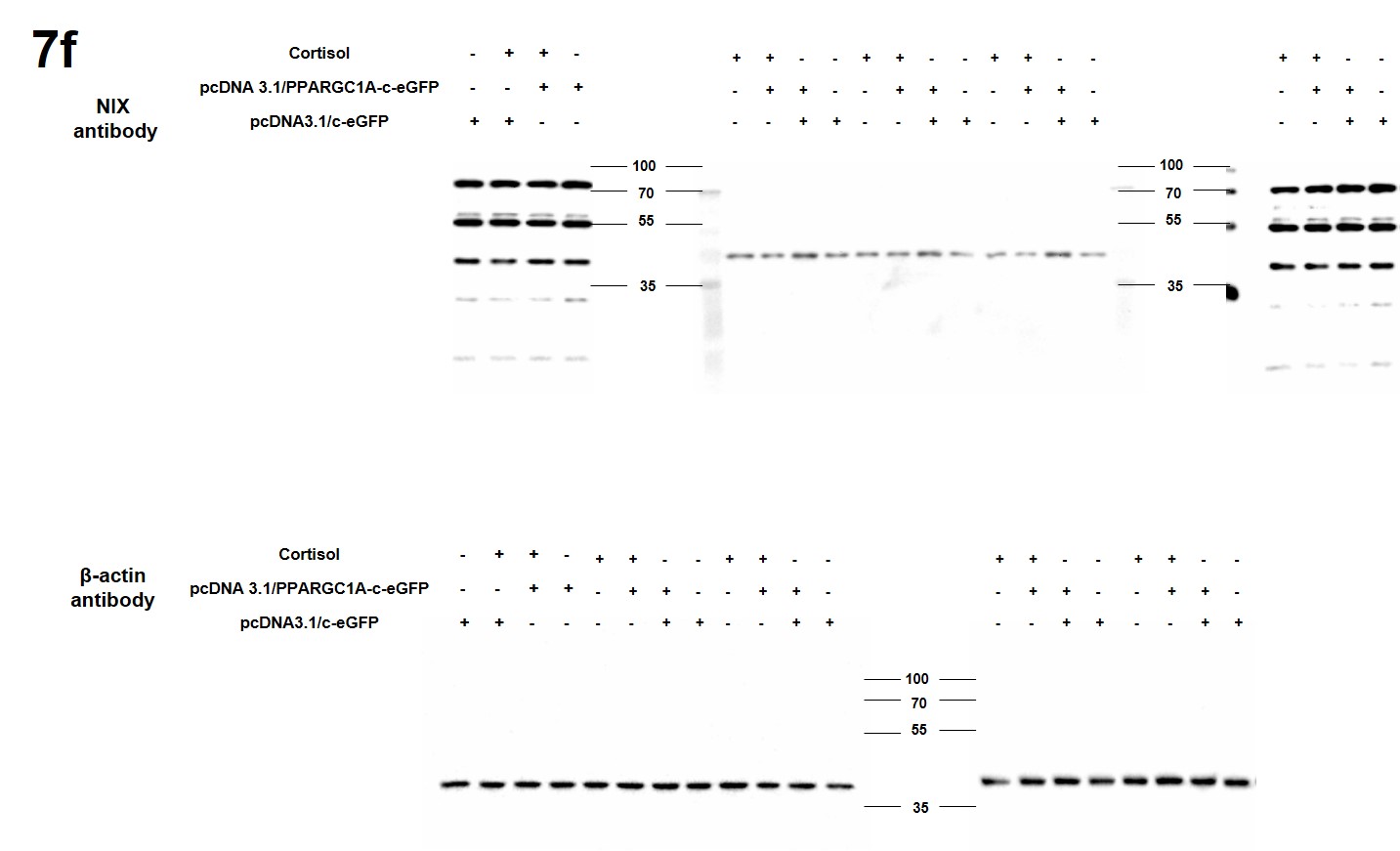

Supplement: Supplementary file 4 — Source Data [file 41467_2020_20679_MOESM4_ESM.zip › Fig 7/Fig 7f.jpg]

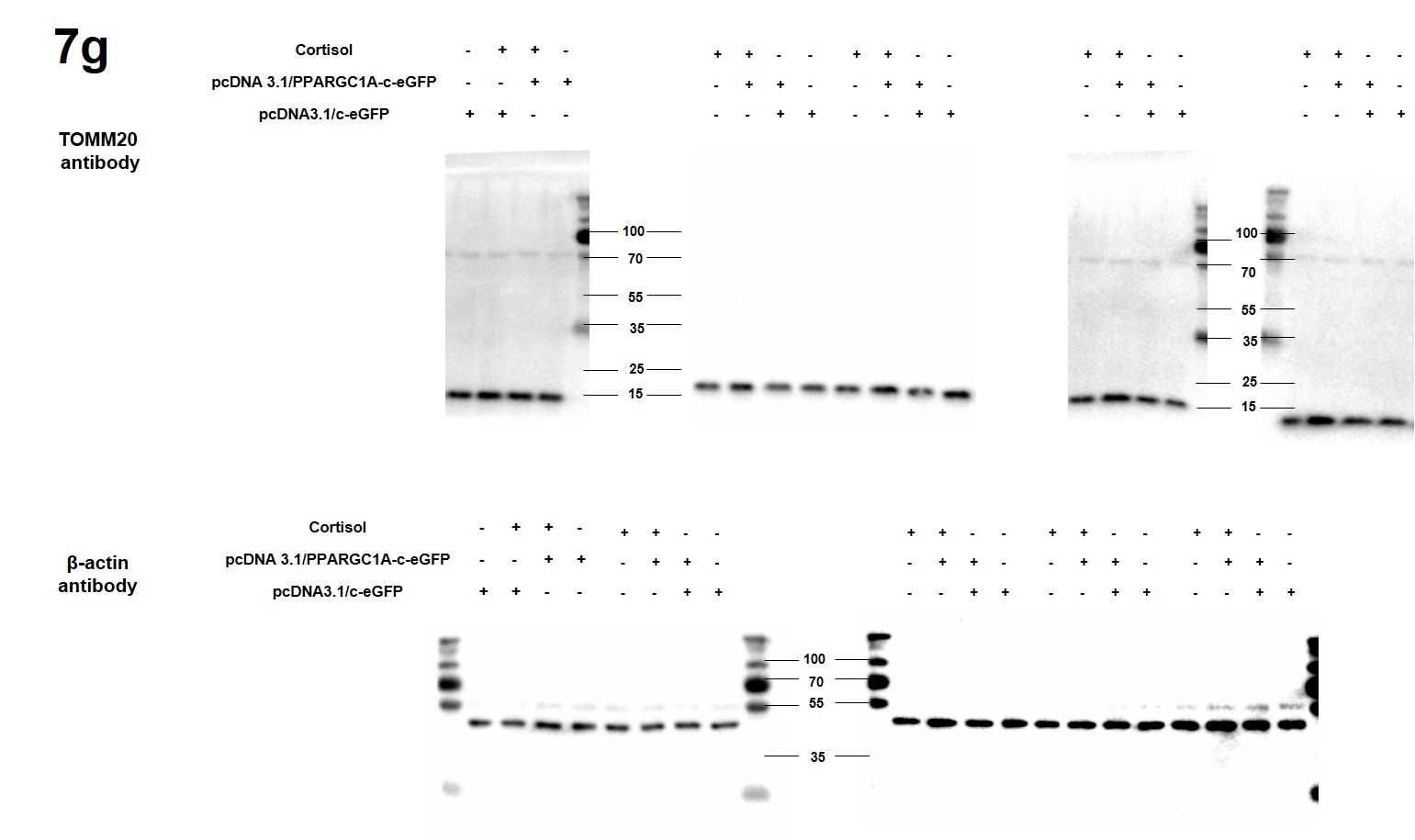

Supplement: Supplementary file 4 — Source Data [file 41467_2020_20679_MOESM4_ESM.zip › Fig 7/Fig 7g.jpg]

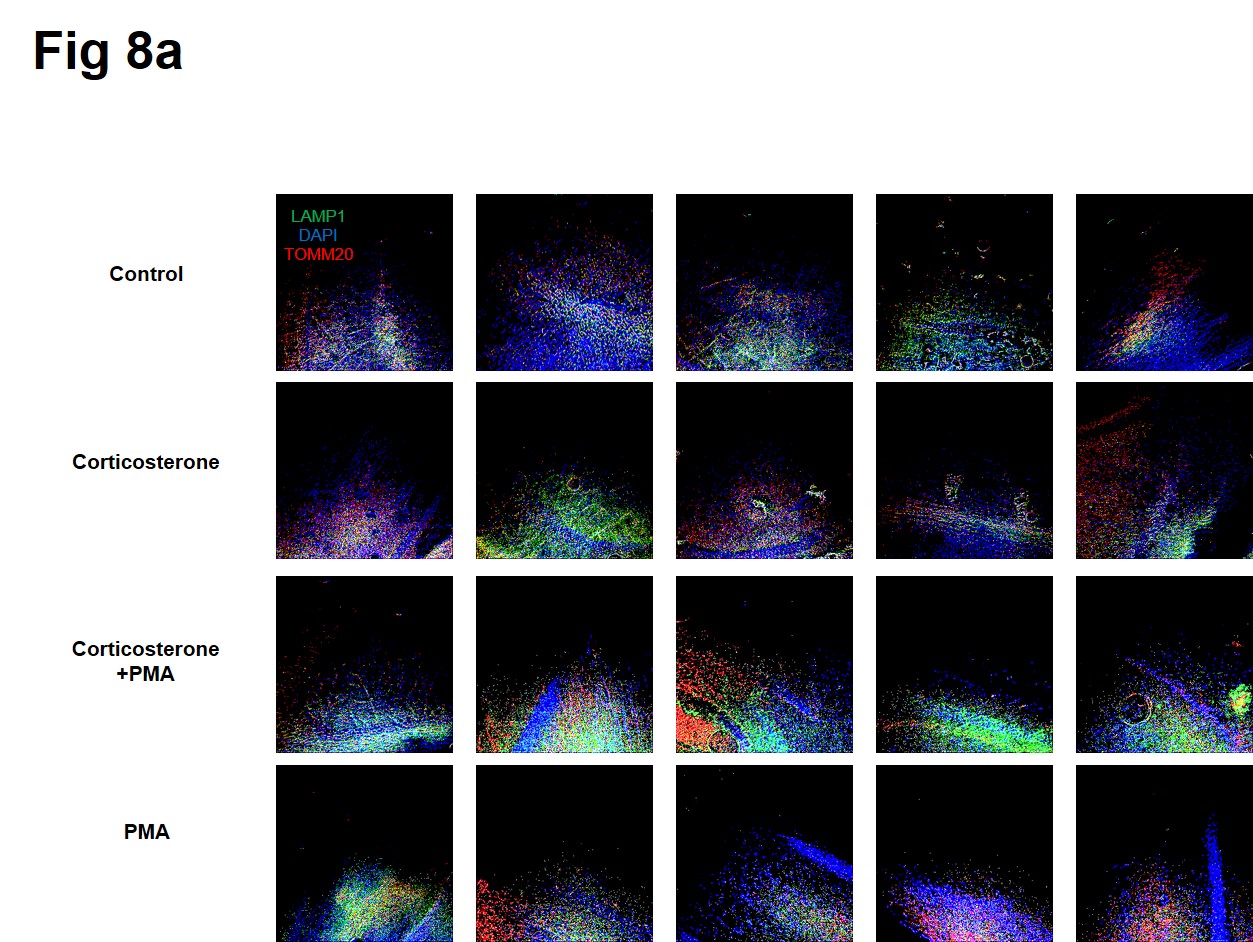

Supplement: Supplementary file 4 — Source Data [file 41467_2020_20679_MOESM4_ESM.zip › Fig 8/Fig 8a.jpg]

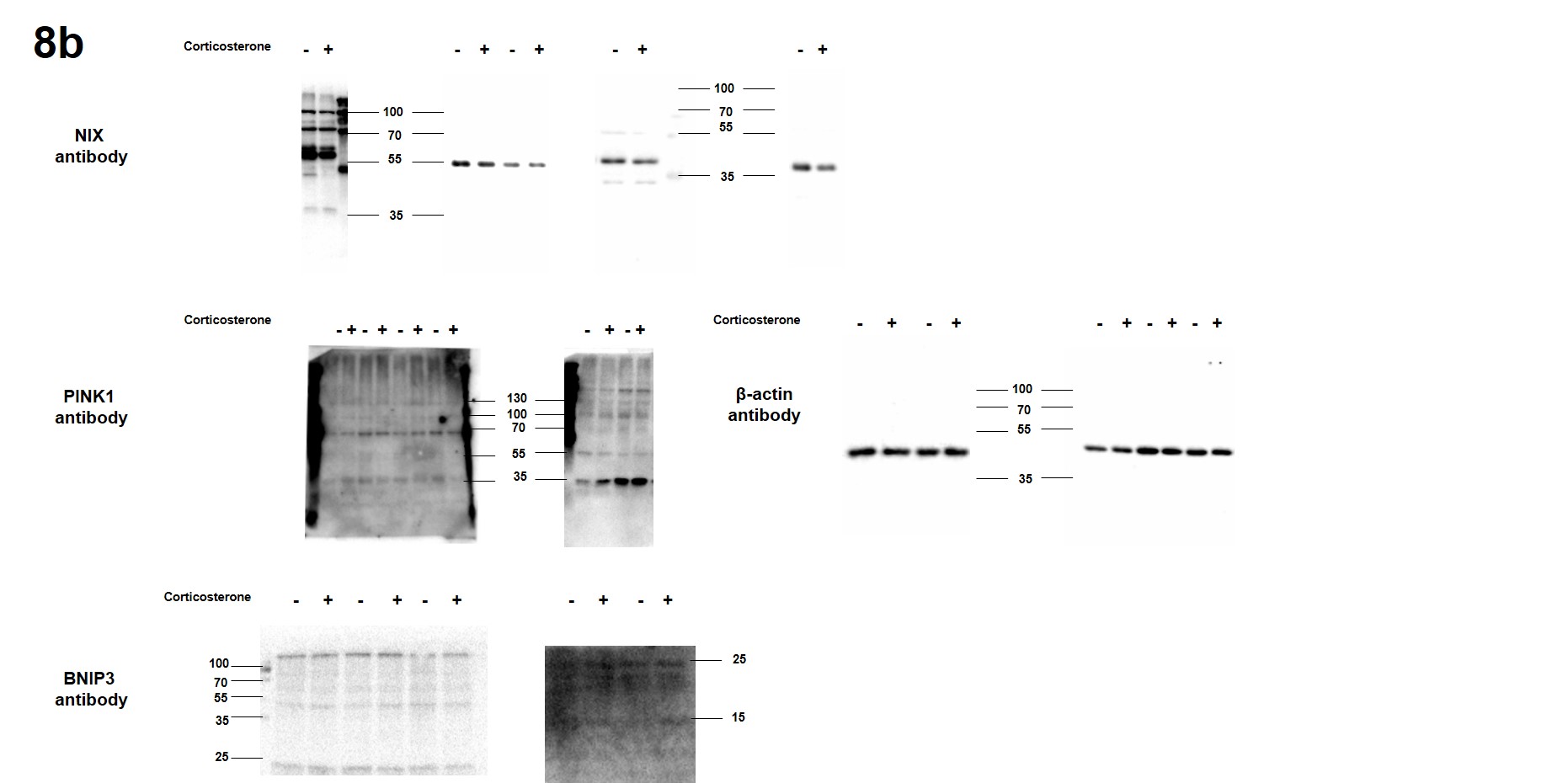

Supplement: Supplementary file 4 — Source Data [file 41467_2020_20679_MOESM4_ESM.zip › Fig 8/Fig 8b.jpg]

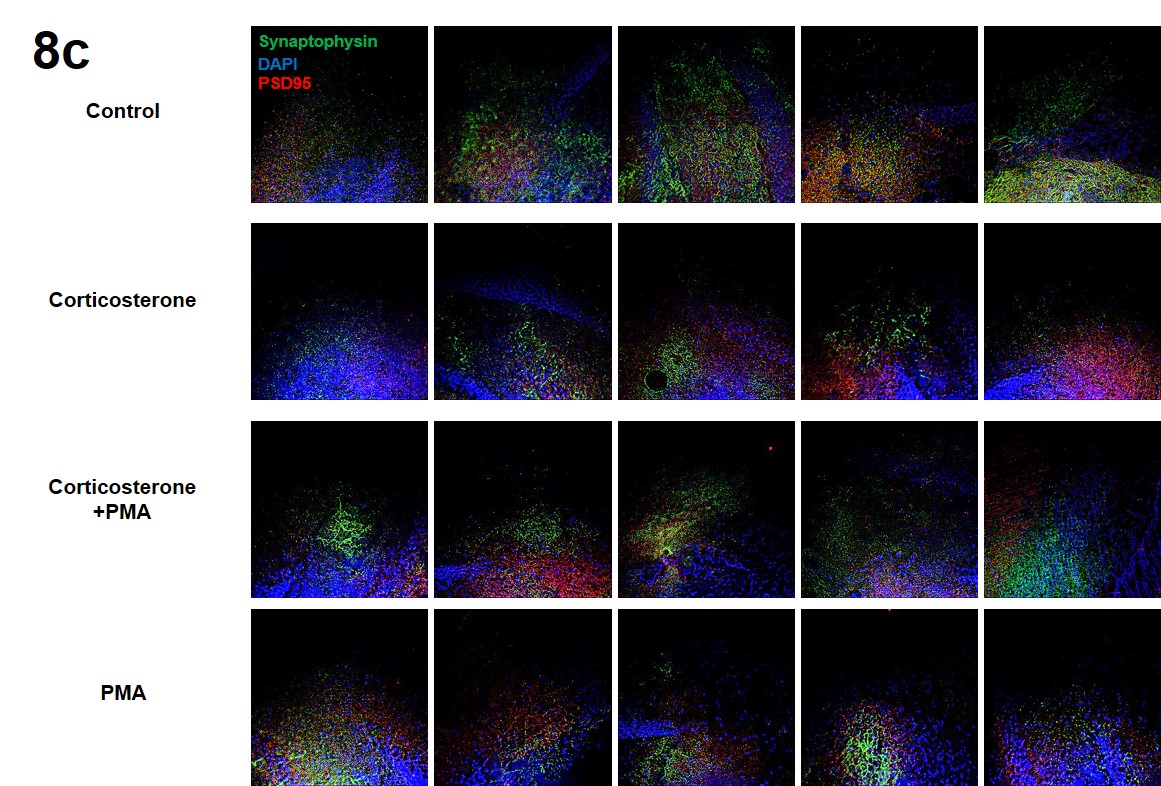

Supplement: Supplementary file 4 — Source Data [file 41467_2020_20679_MOESM4_ESM.zip › Fig 8/Fig 8c.jpg]

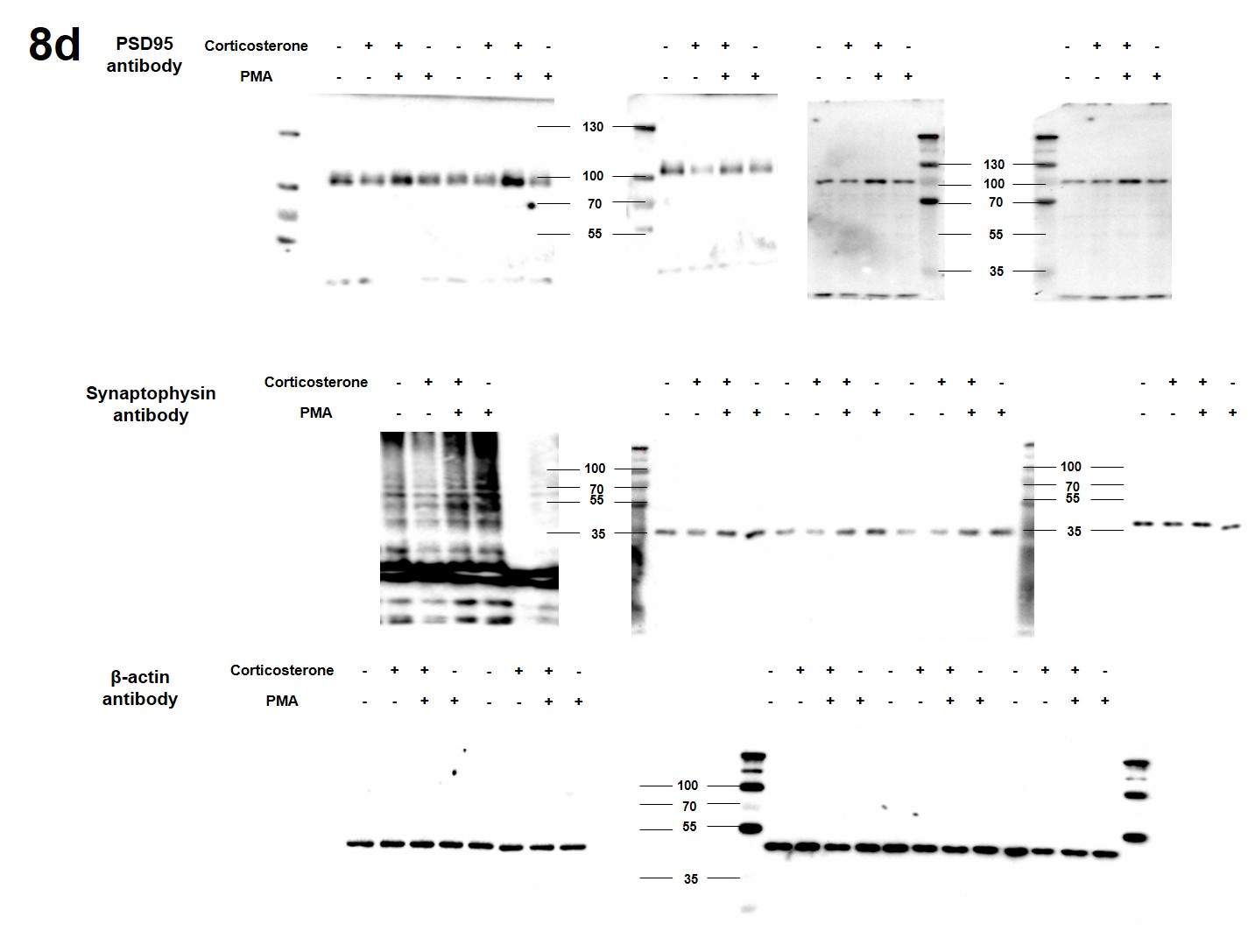

Supplement: Supplementary file 4 — Source Data [file 41467_2020_20679_MOESM4_ESM.zip › Fig 8/Fig 8d.jpg]

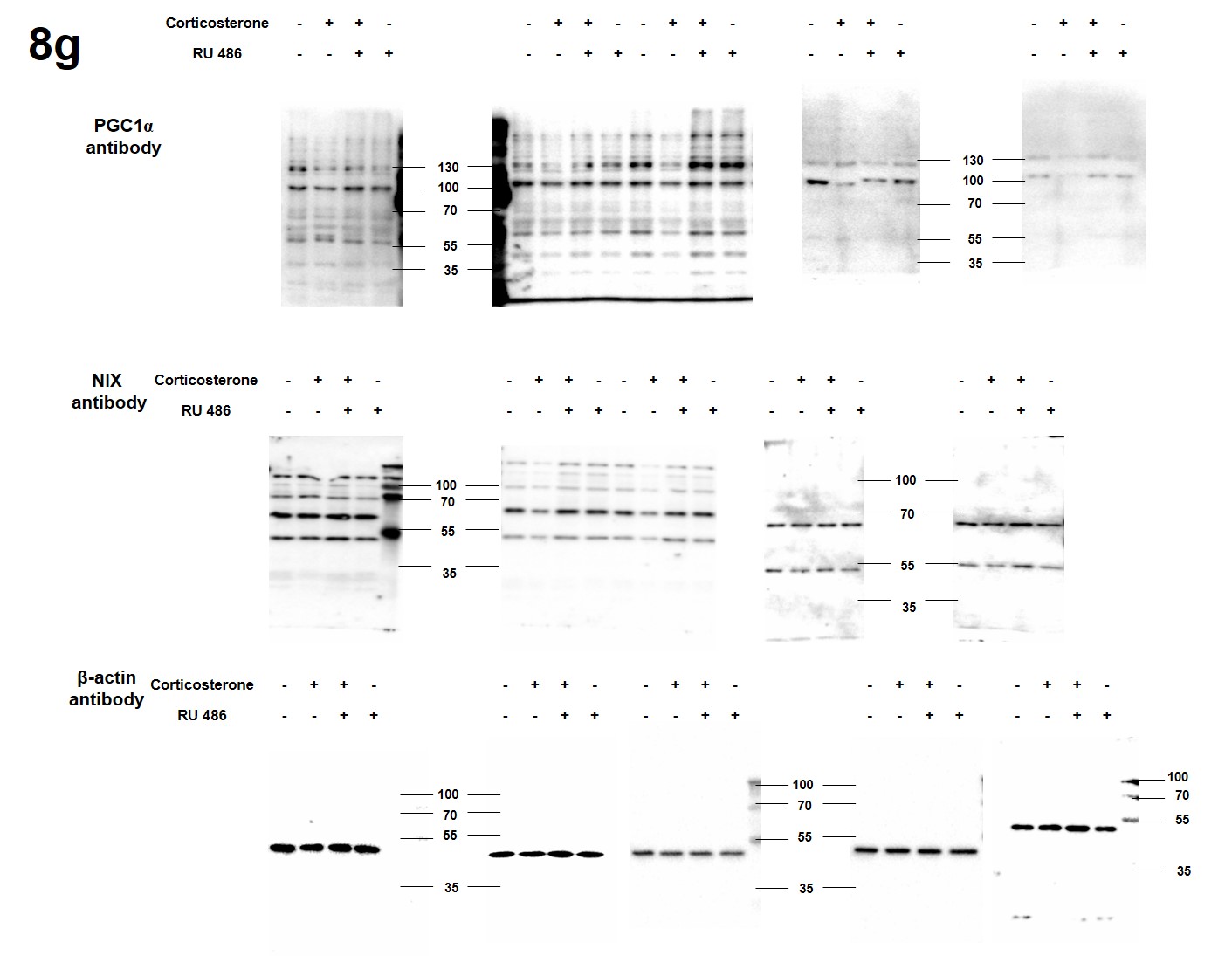

Supplement: Supplementary file 4 — Source Data [file 41467_2020_20679_MOESM4_ESM.zip › Fig 8/Fig 8g.jpg]

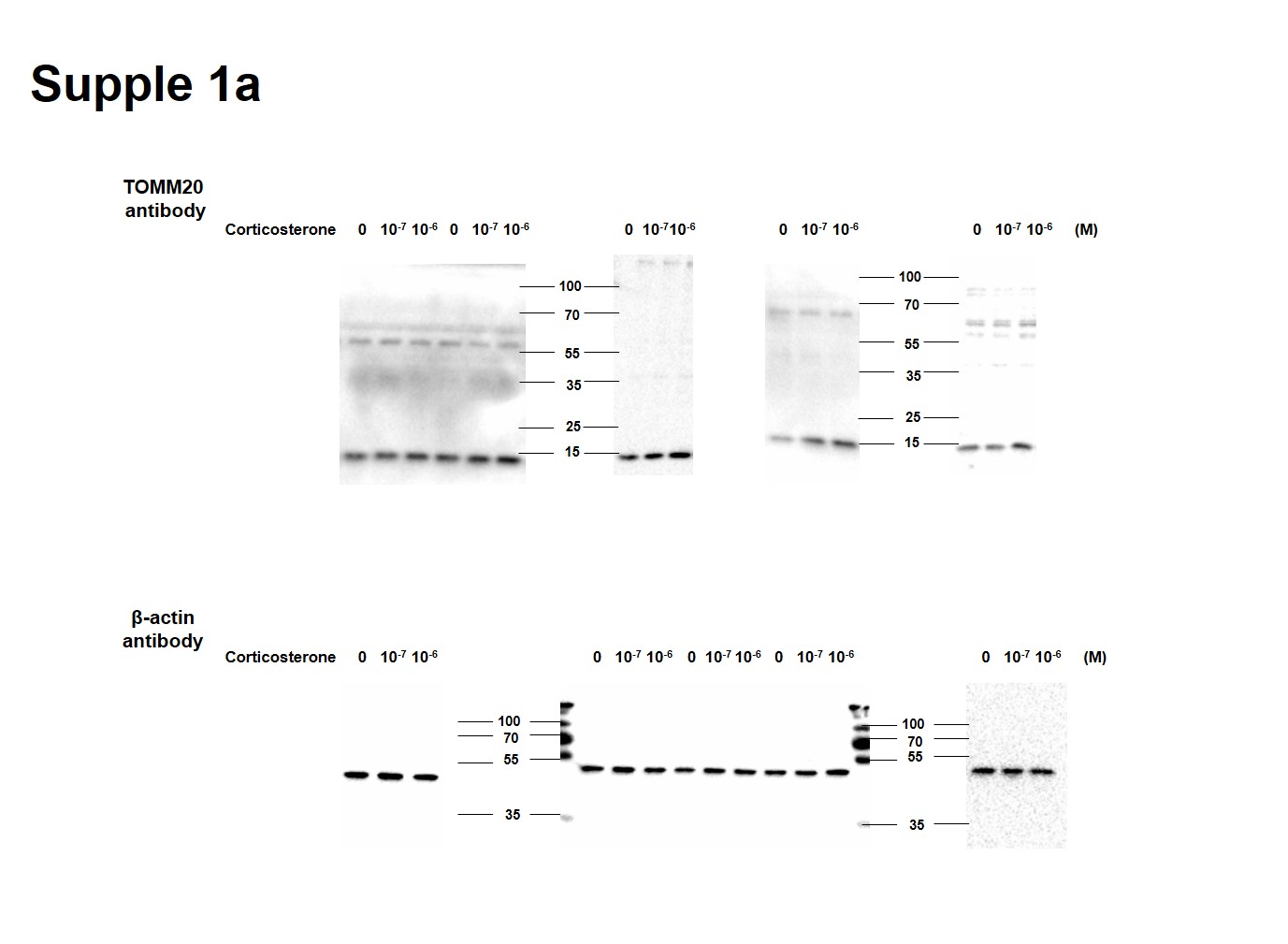

Supplement: Supplementary file 4 — Source Data [file 41467_2020_20679_MOESM4_ESM.zip › Supple Fig 1/Fig 1a.jpg]

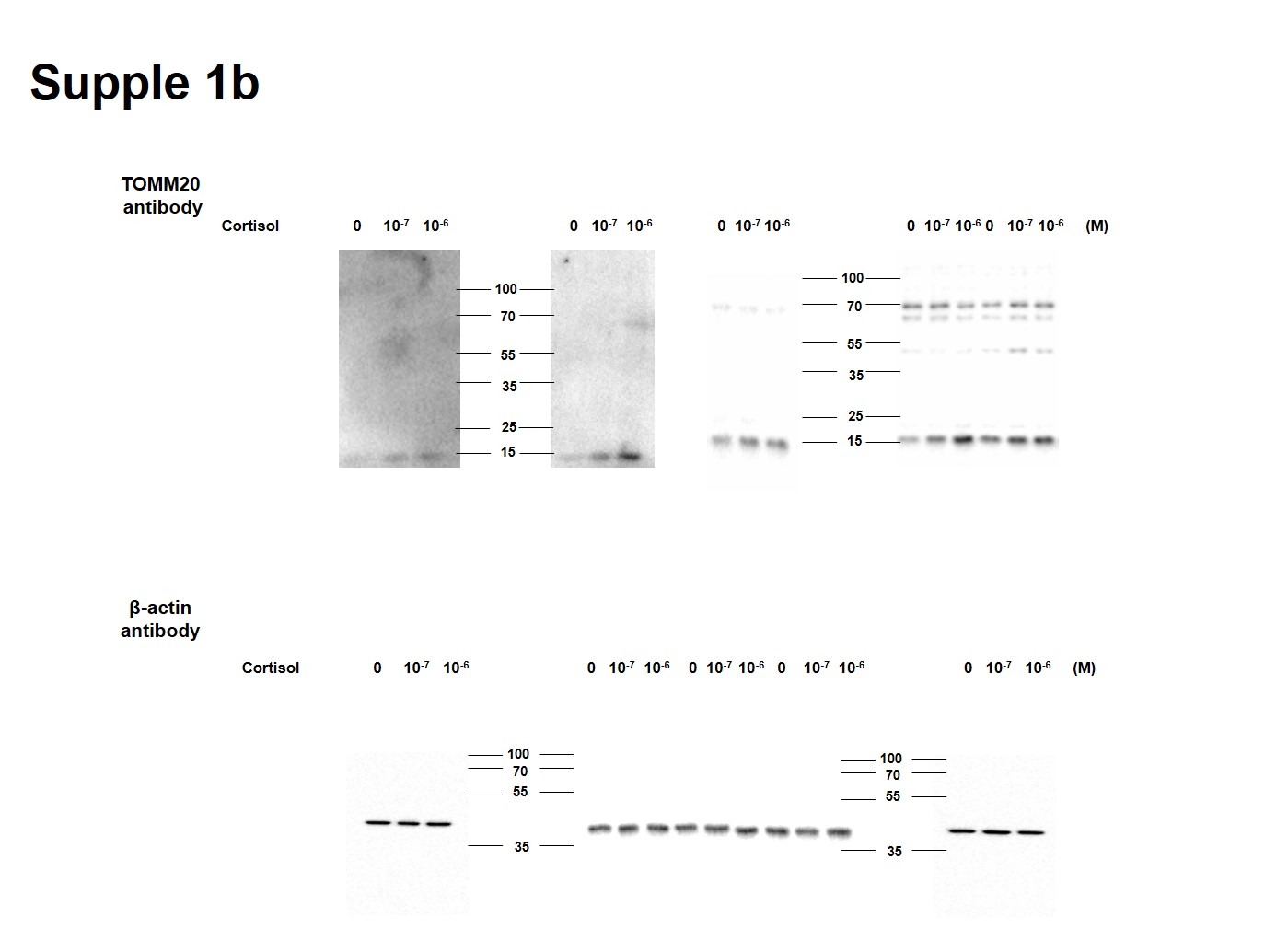

Supplement: Supplementary file 4 — Source Data [file 41467_2020_20679_MOESM4_ESM.zip › Supple Fig 1/Fig 1b.jpg]

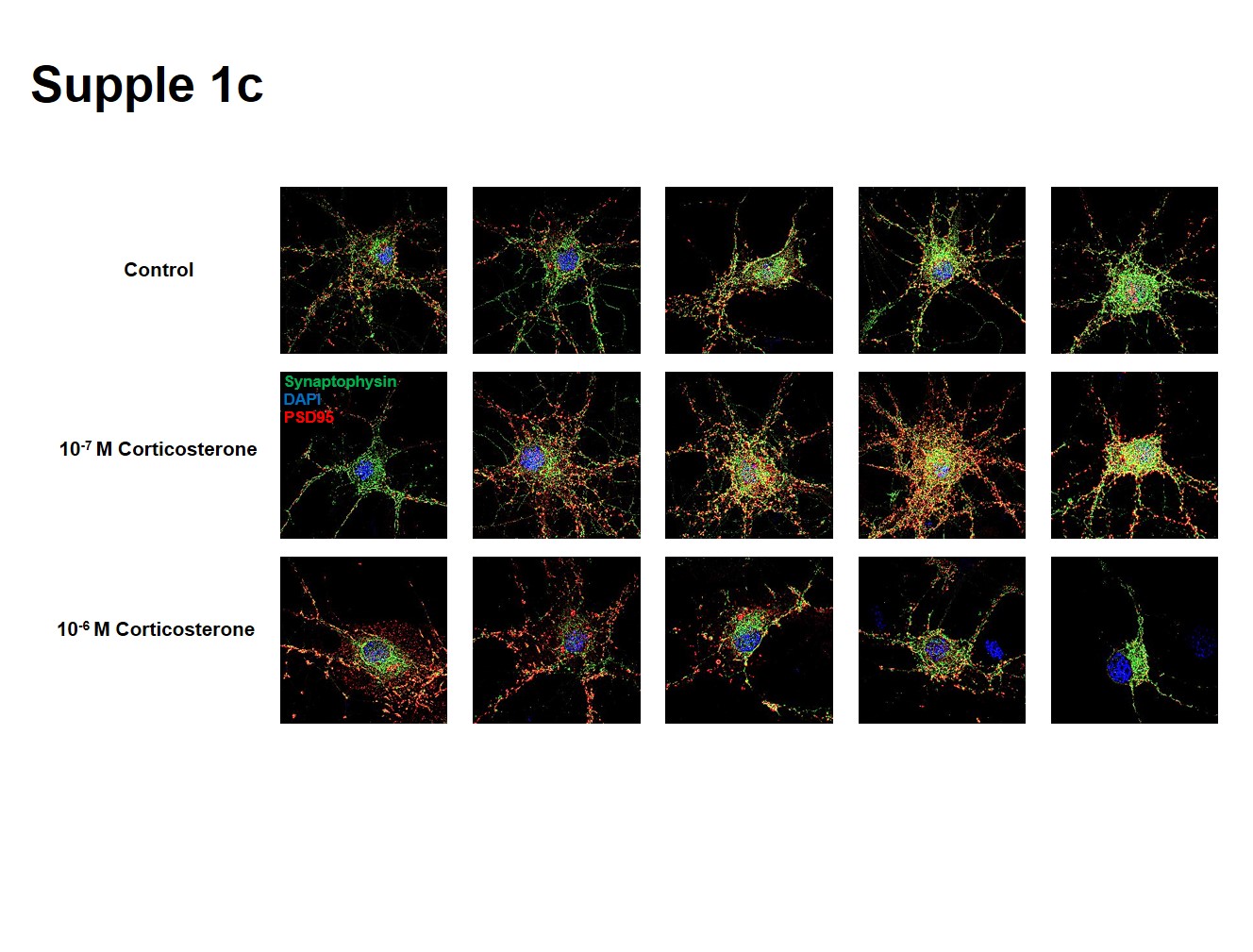

Supplement: Supplementary file 4 — Source Data [file 41467_2020_20679_MOESM4_ESM.zip › Supple Fig 1/Fig 1c.jpg]

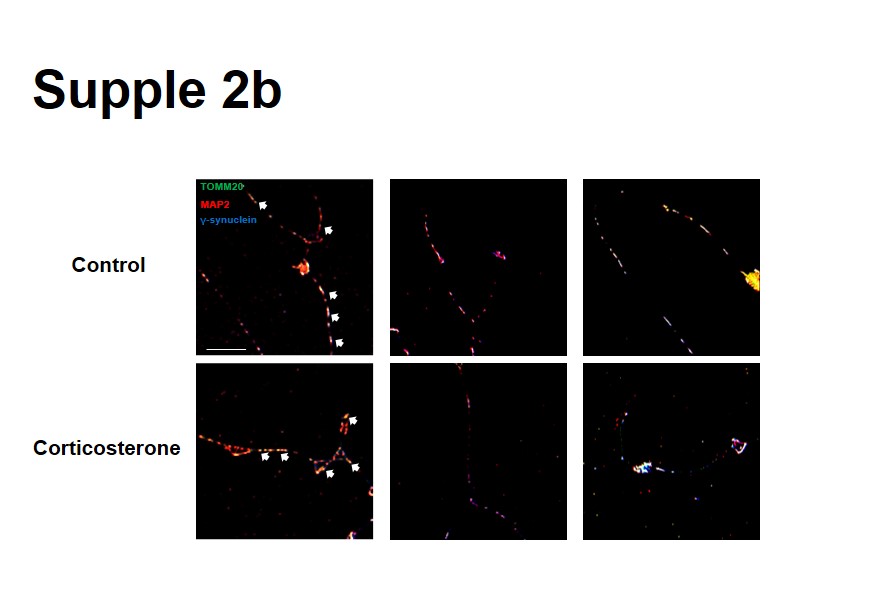

Supplement: Supplementary file 4 — Source Data [file 41467_2020_20679_MOESM4_ESM.zip › Supple Fig 2/Fig 2b.jpg]

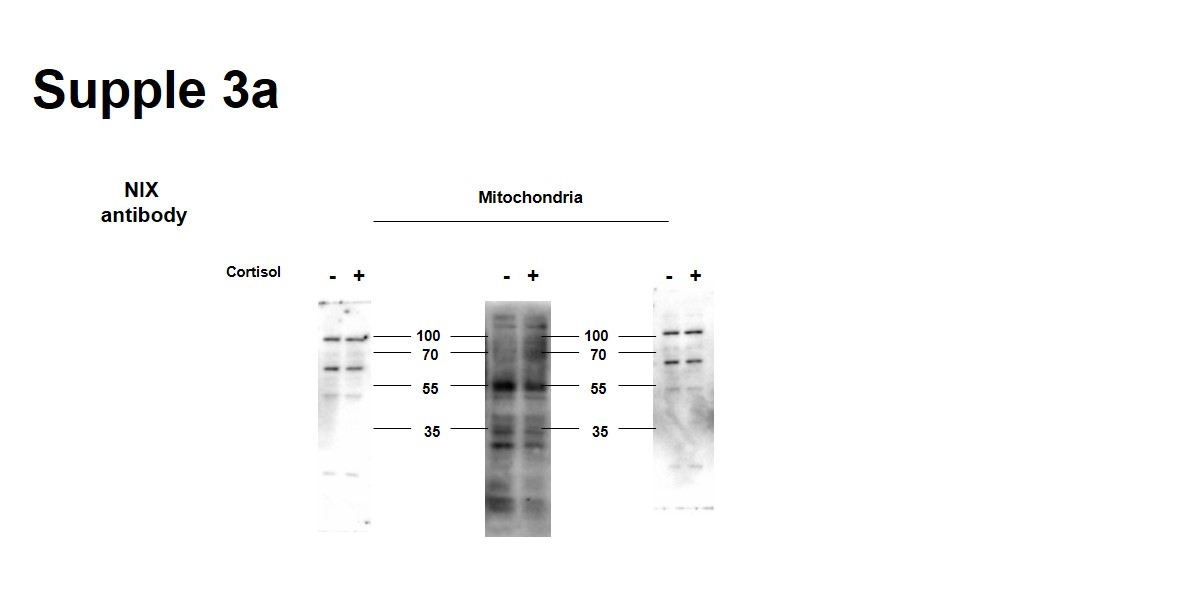

Supplement: Supplementary file 4 — Source Data [file 41467_2020_20679_MOESM4_ESM.zip › Supple Fig 3/Fig 3a.jpg]

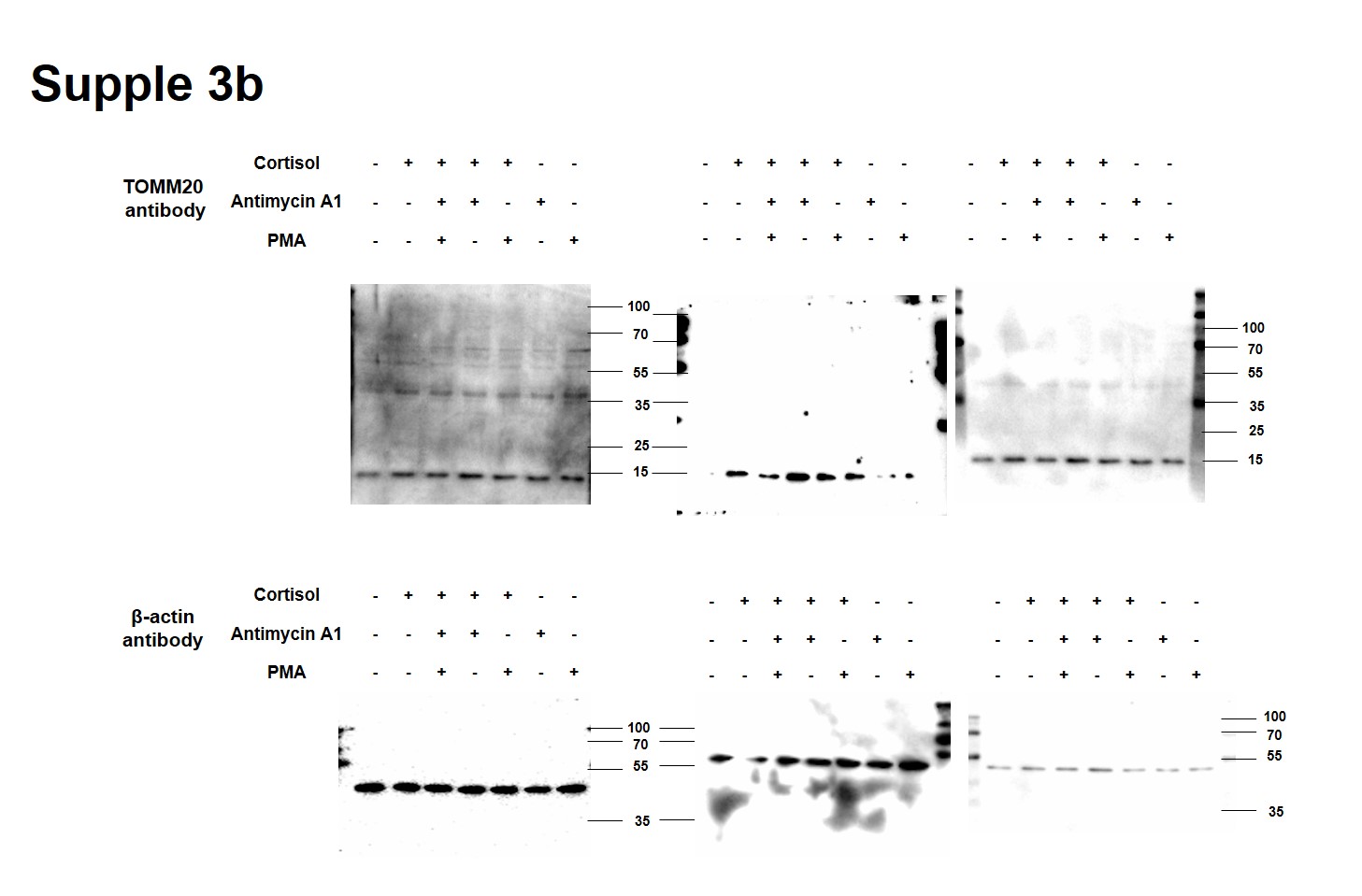

Supplement: Supplementary file 4 — Source Data [file 41467_2020_20679_MOESM4_ESM.zip › Supple Fig 3/Fig 3b.jpg]

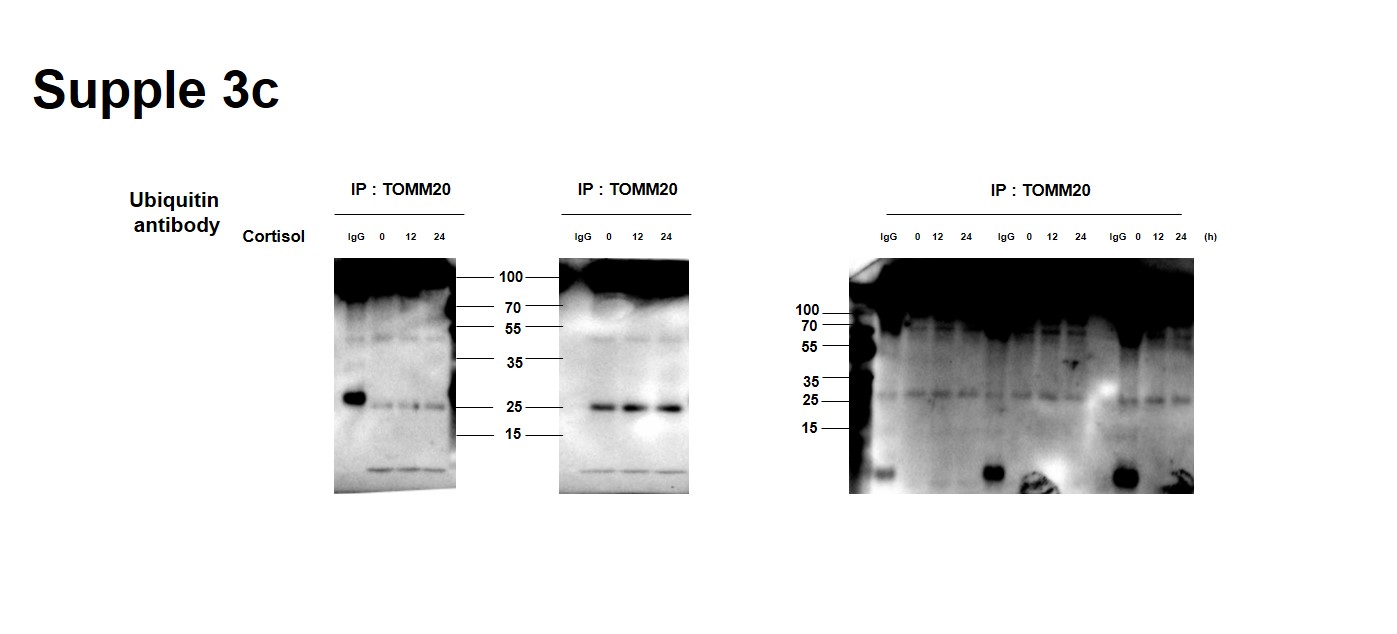

Supplement: Supplementary file 4 — Source Data [file 41467_2020_20679_MOESM4_ESM.zip › Supple Fig 3/Fig 3c.jpg]

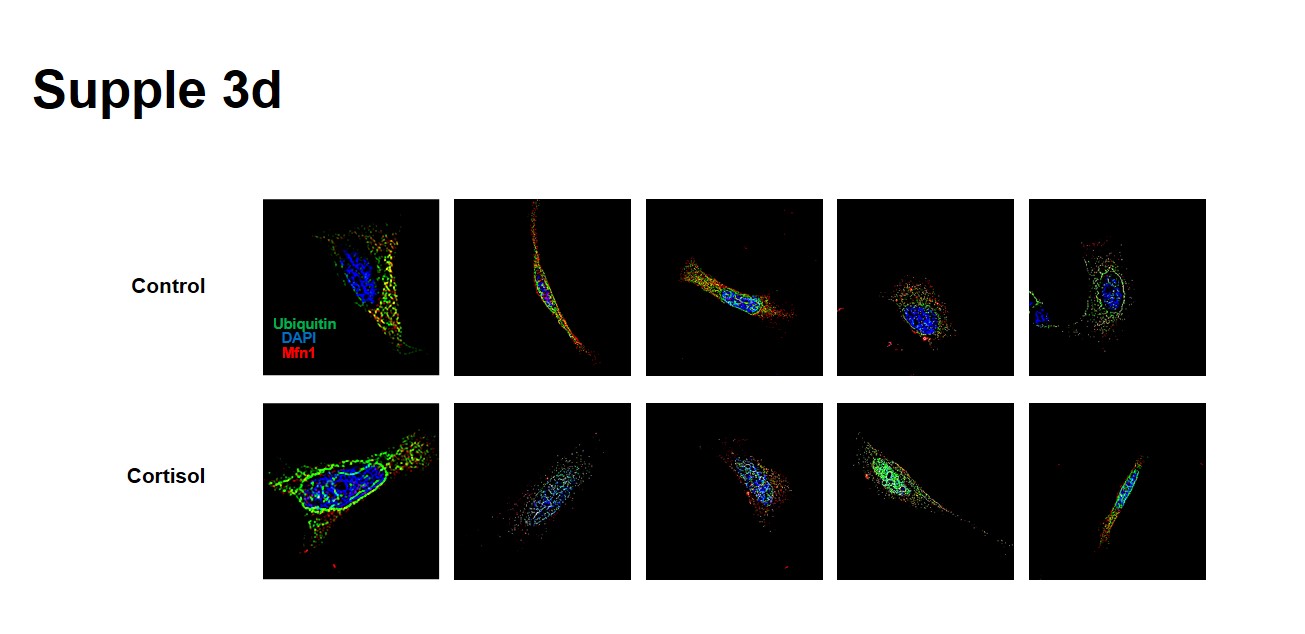

Supplement: Supplementary file 4 — Source Data [file 41467_2020_20679_MOESM4_ESM.zip › Supple Fig 3/Fig 3d.jpg]

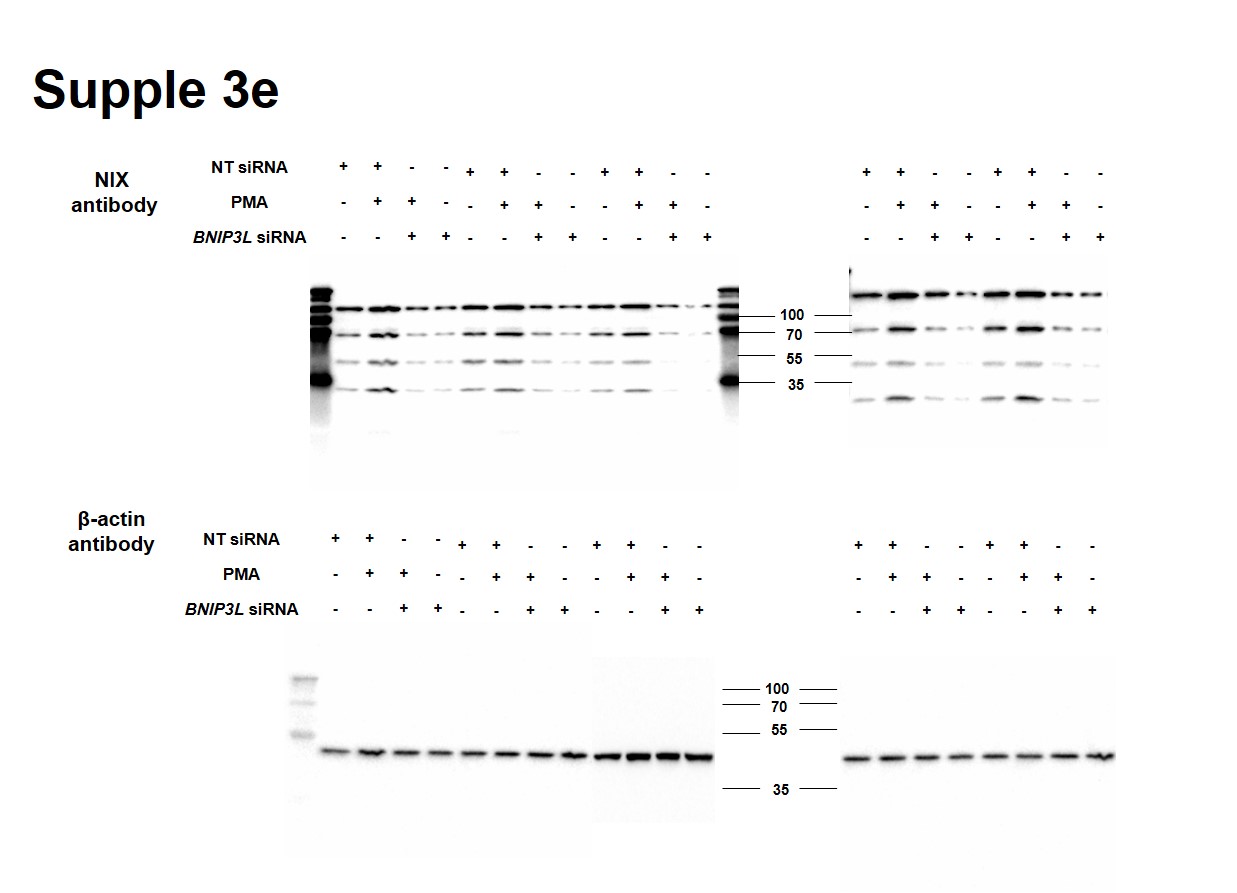

Supplement: Supplementary file 4 — Source Data [file 41467_2020_20679_MOESM4_ESM.zip › Supple Fig 3/Fig 3e.jpg]

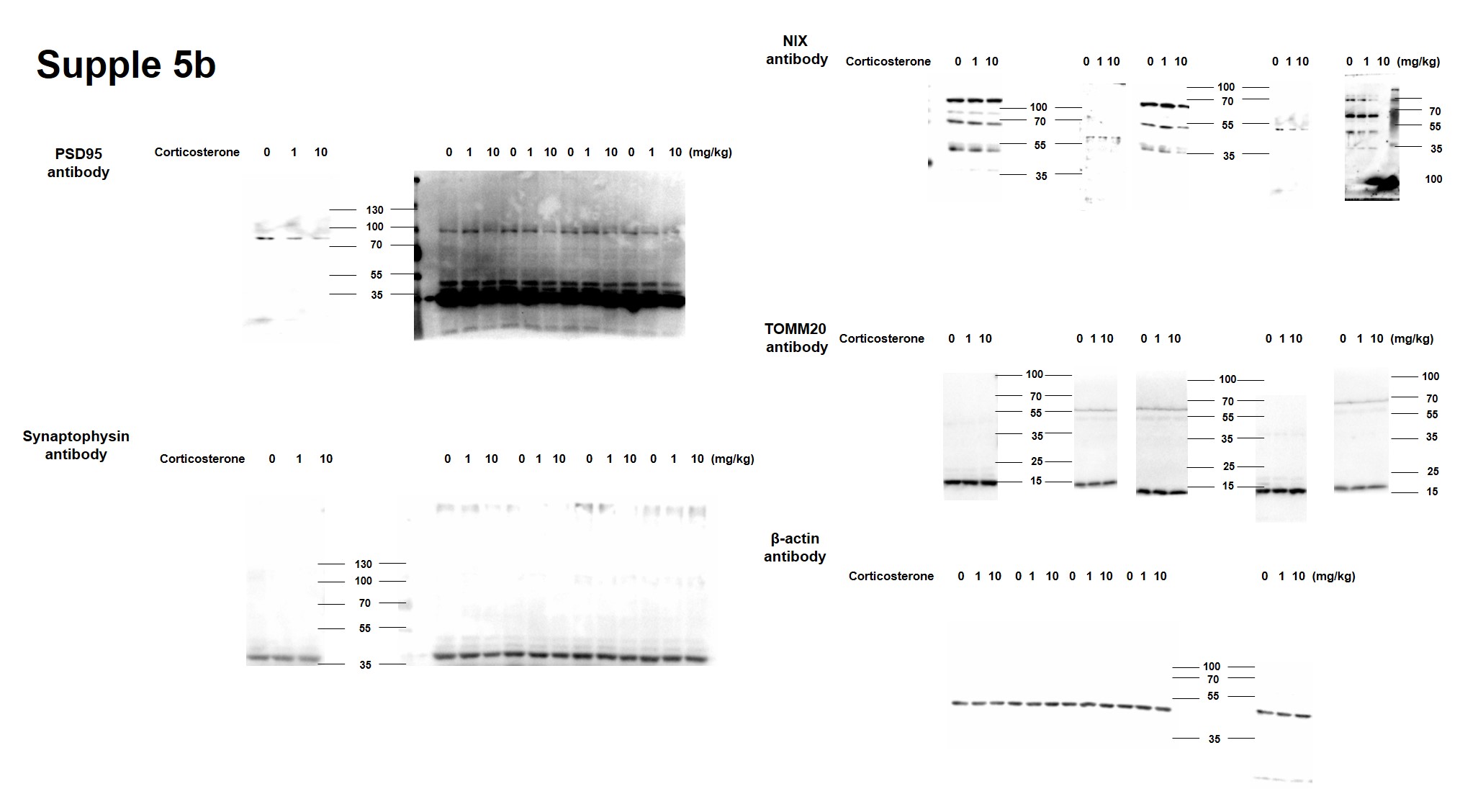

Supplement: Supplementary file 4 — Source Data [file 41467_2020_20679_MOESM4_ESM.zip › Supple Fig 5/Fig 5b.jpg]
